# New Genetic Loci Implicated in Cardiac Morphology and Function Using Three Dimensional Population Phenotyping

**DOI:** 10.1161/CIRCGEN.124.005116

**Published:** 2025-10-07

**Authors:** Chang Lu, Kathryn A. McGurk, Sean L. Zheng, Antonio de Marvao, Paolo Inglese, Wenjia Bai, James S. Ware, Declan P. O’Regan

**Affiliations:** 1 MRC Laboratory of Medical Sciences, Imperial College London, Hammersmith Hospital Campus; 2 National Heart and Lung Institute, Imperial College London, London, United Kingdom; 3 Department of Computing, Imperial College London, London, United Kingdom; 4 Department of Brain Sciences, Imperial College London, London, United Kingdom; 5 Program in Medical and Population Genetics, The Broad Institute of MIT & Harvard, Cambridge, MA; 6 Department of Cardiology, Imperial College Healthcare NHS Trust; 7 Royal Brompton & Harefield Hospitals, Guy’s and St. Thomas’ NHS Foundation Trust; 8 Department of Women and Children’s Health; 9 British Heart Foundation Centre of Research Excellence, School of Cardiovascular and Metabolic Medicine and Sciences, King’s College London, London, United Kingdom

**Keywords:** left ventricle, genome wide association study, three dimensional, hypertrophy, wall thickness

## Abstract

**Background:**

Cardiac remodeling occurs in the mature heart and is a cascade of adaptations in response to stress that are primed in early life. A key question remains as to the processes that regulate the geometry and motion of the heart, and how it adapts to stress.

**Methods:**

We performed spatially-resolved phenotyping using machine learning-based analysis of cardiac magnetic resonance imaging in 47,549 UK Biobank participants. We analyzed 16 left ventricular spatial phenotypes, including regional myocardial wall thickness and systolic strain in both circumferential and radial directions. In up to 40,058 participants genetic associations across the allele frequency spectrum were assessed using genome-wide association studies (GWAS) with imputed genotypes participants, and exome-wide association studies (ExWAS) and gene-based burden tests using whole-exome sequencing data. We integrated transcriptomic data from the GTEx project and used pathway enrichment analyses to further interpret the biological relevance of identified loci. To investigate causal relationships, we conducted Mendelian randomization analyses to evaluate the effects of blood pressure on regional cardiac traits, and the effects of these traits on cardiomyopathy risk.

**Results:**

We found 42 loci associated with cardiac structure and contractility, many of which reveal patterns of spatial organization in the heart. Whole-exome sequencing revealed three additional variants not captured by GWAS, including a missense variant in *CSRP3* (minor allele frequency 0.5%). The majority of newly discovered loci are found in cardiomyopathy-associated genes suggesting they regulate spatially-distinct patterns of remodelling in the left ventricle in an adult population. Our causal analysis also found regional modulation of blood pressure on cardiac wall thickness and strain.

**Conclusion:**

These findings provide a comprehensive description of the pathways that orchestrate heart development and cardiac remodelling. These data highlight the role that cardiomyopathy-associated genes have on the regulation of spatial adaptations in those without known disease.

## Non-standard Abbreviations and Acronyms

CMR: cardiac magnetic resonance imaging
HCM: hypertrophic cardiomyopathy
DCM: dilated cardiomyopathy
TWAS: transcriptome-wide association study
ExWAS: variant level exome-wide association study

## Introduction

Diverse cardiovascular cell types coordinate to form spatially organised structures of the human heart.^1^ Interactions between biophysical mechanics, morphogenesis and cell fate during heart development shape its three dimensional structure.^2^ Cardiac remodelling occurs in the mature heart and is associated with the development and progression of ventricular dysfunction which is manifest by changes in geometry and contractility that are regulated by mechanical, cellular and genetic factors.^3^ Remodelling complicates many cardiovascular disorders and has an effect across diverse cell types and anatomic domains. During cardiac development exposure to diverse stimuli prime gene expression profiles that drive phenotypic and functional adaptations in later life.^4^ Cardiomyopathies in particular are characterised by diverse morphofunctional phenotypes that relate to the additive effect of common and rare variants on the sensitivity to environmental stimuli.^5,6^ While several candidate genes have been implicated in global structural phenotypes through genome-wide association studies (GWASs),^7,8^ the genetic architecture of cardiac geometry and contractile function, as well as associations with cardiomyopathic states, remain poorly characterised.

**Figure 1:**
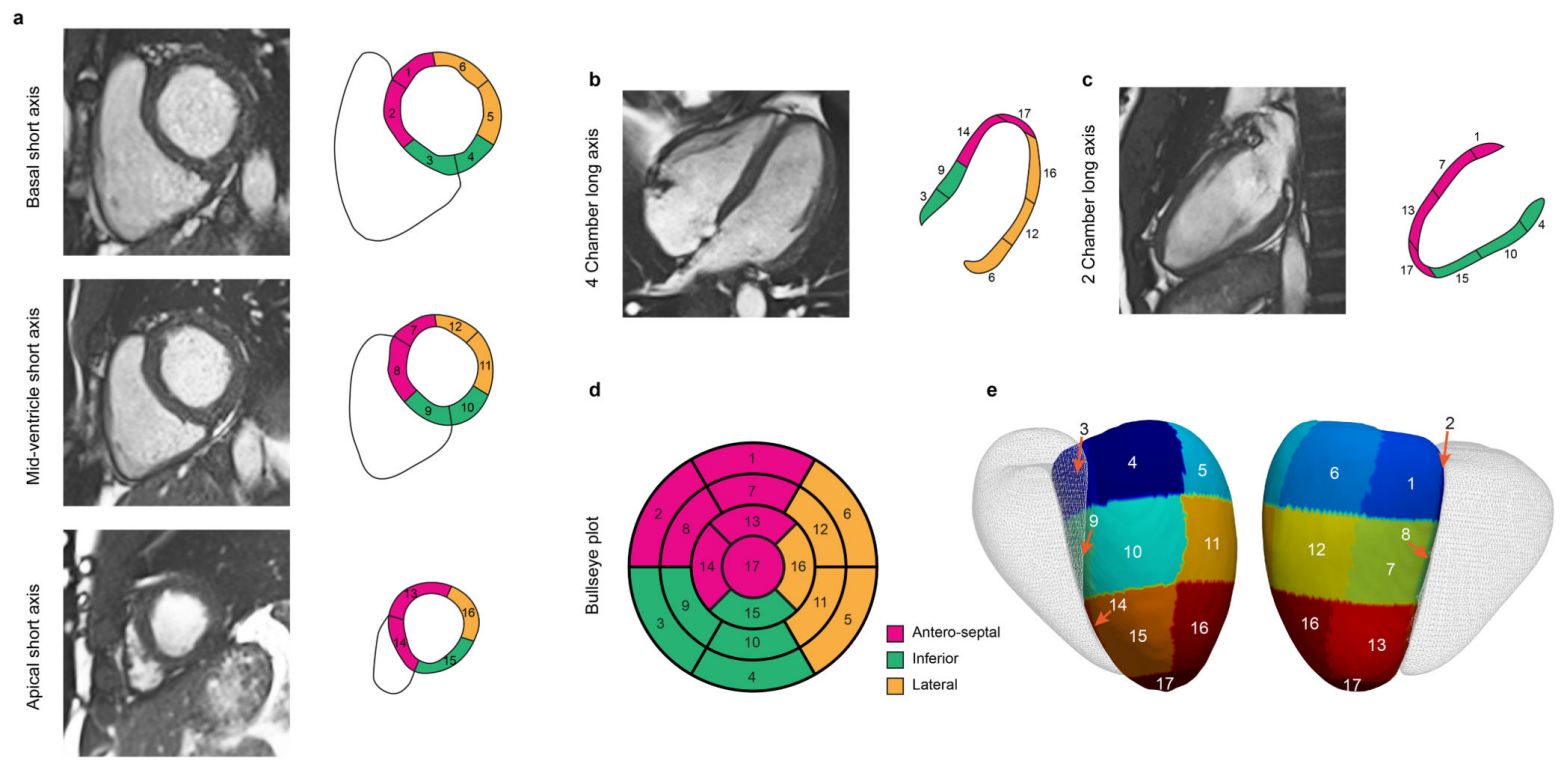
Cardiac image analysis. Cardiac magnetic resonance (CMR) cine imaging was performed in short and long axis planes. A fully convolutional neural network was used to segment the left ventricular myocardium and determine regional wall thickness. Motion tracking was performed using image registration to map myocardial deformation between frames and calculate regional Eulerian strain. The left ventricle was divided into 17 segments using the American Heart Association (AHA) model. Short axis (**a**), four chamber long axis (**b**) and two chamber long axis (**c**) CMR imaging with corresponding segments. Segments are grouped by anatomic location and numbered sequentially allowing characteristics to be represented on bullseye (**d**) and three dimensional (**e**) models of the left ventricle (right ventricle shown in outline).

The structure and function of the heart can be assessed through detailed mapping of wall thickness throughout the left ventricle as well quantifying regional deformation to assess myocardial contractility. Here we use data from over 40,000 participants in UK Biobank with cardiac magnetic resonance imaging (CMR) and apply deep-learning computer vision techniques to enable spatially-resolved phenotyping of left ventricular geometry and motion (Figure 1).^9,10^ This provides a three-dimensional time-resolved atlas of the heart to perform genetic association studies across the allele frequency spectrum. We report the effect of environmental and genetic factors on the molecular orchestration of cardiac shape, motion and remodelling in adults. This approach shows newly associated loci only discovered through precision cardiac phenotyping, describes the regional pattern of genetic variants associated with cardiomyopathy, and reveals conserved pathways that regulate multi-organ development.

## Methods

Full methods are available in the Supplemental Material. The code used in the study is available on GitHub (https://github.com/ImperialCollegeLondon/spatial_cardiac_GWAS). Genetic analysis were performed on the UK Biobank Research Analysis Platform (RAP). All raw and derived data in this study are available to approved researchers from UK Biobank (https://biobank.ndph.ox.ac.uk/), accessed under application number 40616. The National Research Ethics Service approved the UK Biobank study (11/NW/0382). All participants gave written informed consent.

## Results

### Study overview

We analysed CMR data from up to 47,549 participants of UK Biobank. Deep learning segmentation and motion tracking was used to assess cardiac structure and motion throughout the left ventricle. Data are summarised by anatomical region using the American Heart Association (AHA) 17 segment model (excluding the apex). For each segment mean wall thickness (WT) at end diastole, mean peak circumferential myocardial strain (strain^circ^) and mean peak radial myocardial strain (strain^rad^) were estimated on short-axis imaging using validated pipelines.^9,11^ In addition, conventional global measures of left ventricular mass and volume were derived.

We assessed patterns of spatial correlation of phenotypes across the left ventricle using biophysical and haemodynamic co-variates (Supplementary Table 1). Phenome wide association studies (PheWAS) were performed on these traits after known confounders had been adjusted for. Genome wide association studies (GWAS), variant-level exome-wide association studies (ExWAS), and gene-level exome-wide burden tests were performed on up to 40,058 healthy individuals of white British ancestry without cardiomyopathy for 54 spatial and global traits. We performed Mendelian randomisation (MR) to analyse potential causal associations between haemodynamic factors and regional wall thickness. To understand the relationship between genetic factors regulating spatial physiology and the patho-physiology of cardiac remodelling we performed genetic correlation and causal association analysis with hypertrophic (HCM) and dilated (DCM) cardiomyopathies.

### Spatial correlations in left ventricular traits

**Figure 2:**
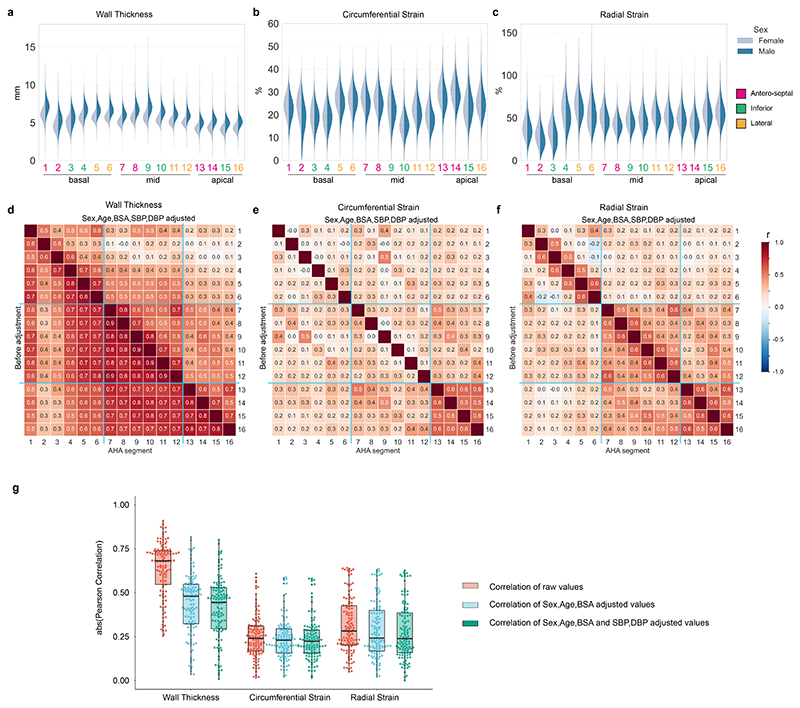
Correlations between spatial left ventricle (LV) traits and association to known predictors. (**a-c**) Distribution of regional traits in up to 47,549 participants by sex are shown with violin plots. Scott’s rule was used to determine the smoothing bandwidth for the kernel estimation. Inner dotted lines show the data quartiles. (**d-f**) Pearson’s correlation among the 16 regional wall thickness (WT), strain^circ^, and strain^rad^ before (lower diagonal) and after (upper diagonal) adjustment by the indicated confounding factors. The correlation coefficients are shown as heatmaps. The color indicates a correlation efficient from -1 to 1 (blue to red). The basal, mid-cavity and apical segments are separated by light blue lines. (**g**) Distribution of regional trait Pearson’s correlations before and after adjustment by the indicated confounding factors. The boxes show the quartiles of the dataset, and all values are overlaid as dots.

To understand potential drivers of regional phenotypic variation in the heart we assessed spatial correlations in wall thickness and strain across the left ventricle. There was a strong correlation of unadjusted wall thickness between segments, apart from in the basal septum which showed a moderate correlation (Figure 2a to d). Sex, body surface area (BSA), and blood pressure were significantly associated with wall thickness globally (Supplementary Figure 1). After adjustment for these parameters the spatial correlations in wall thickness were reduced to low or moderate (Figure 2g) suggesting other unmeasured factors contribute to variation in hypertrophy.

**Figure 3:**
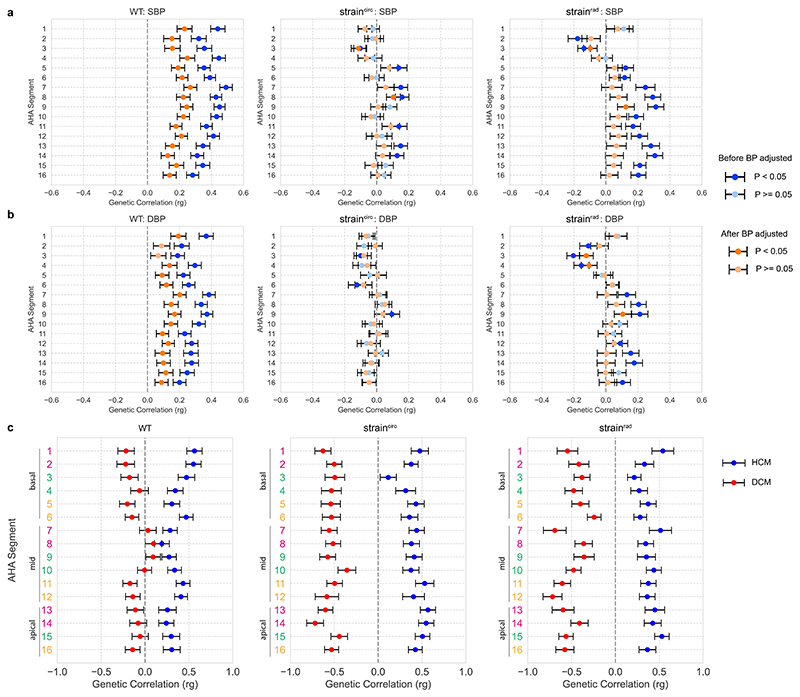
Genetic correlation (*r_g_*) between spatial left ventricular traits, blood pressure and cardiomyopathies. The *r_g_* between spatial traits and both systolic blood pressure (SBP) (**a**) and diastolic blood pressure (DBP) (**b**) were calculated before (blue) and after (orange) adjustment for BP at the imaging visit. GWAS of spatial left ventricular traits was performed on up to 40,058 UK Biobank participants of white British ancestry. Summary statistics of SBP and DBP came from a GWAS on up to 801,644 individuals.^13^ Two-sided *P* values were estimated using linkage disequilibrium score regression. Significant correlations (*P* < 0.05) are shown in darker colors. Center values are the estimated *r_g_* and error bars indicate standard error. (**c**) *r_g_* between BP-adjusted spatial traits and hypertrophic cardiomyopathy (HCM) (blue) and dilated cardiomyopathy (DCM) (red). Summary statistics of HCM came from GWAS of 1,733 cases and 6,628 controls,^15^ and DCM from GWAS of 5,521 cases and 397,323 controls.^16^

Genetic correlation (r_g_) between complex traits estimates the proportion of variance that two traits share due to genetics, providing useful etiological insights.^12^ Blood pressure has a significant r_g_ with mid-ventricular wall thickness.^8^ Given the high phenotypic correlation between both systolic (SBP) and diastolic blood pressure (DBP) with wall thickness, we compared r_g_ of blood pressure with SBP and DBP-adjusted wall thickness (Supplementary Data File 1).^13^ We found that r_g_ remained significantly associated with SBP (*P* < 0.003, [r_g_, 0.13 − 0.27]) for wall thickness across the left ventricle, and with DBP (*P* < 0.04, [r_g_, 0.09 − 0.20] (Figure 3a and b) for all regions except the basal septum, suggesting that there are shared genetic factors that regulate blood pressure and regional wall thickness independent of a hypertrophic stimulus-response. There is also a significant degree of r_g_ between HCM and BP-adjusted spatial wall thickness (*P* < 0.024, [r_g_, 0.19 − 0.56]) (Figure 3c).

We observed weaker spatial correlations between segments for myocardial strain (Figure 2e,f), demonstrating more regional independence in systolic function which would not be captured through global parameters. In contrast to wall thickness, known co-variates (sex, age, BSA, BP) have different patterns of correlation with regional strain (Supplementary Figure 1). For instance, BP is associated with decreased strain^rad^ in the basal septal wall but increased strain^rad^ in mid-ventricular and apical segments (Supplementary Figure 2), and also has opposing directions of genetic correlation (Figure 3a,b). This heterogeneity may reflect anatomic gradients in biomechanical function related to myofibre architecture in the left ventricle.^14^ GREML analysis shows that myocardial strain has lower heritability (*h*^2^, 0.06 _−_ 0.24) than wall thickness (*h*^2^, 0.14 _−_ 0.30), and more unaccounted variance from known co-variates and common SNPs combined (Supplementary Table 2, Supplementary Data File 2). Myocardial strain also has a significant genetic correlation with both HCM and DCM across multiple left ventricular segments (Figure 3c), highlighting shared mechanisms regulating myocyte performance in health and disease.^15,16^

Phenome-wide association studies of spatial traits show both global and anatomically localised patterns of association with 1,840 phenotypes (Supplementary Figures 3 to 5, Supplementary Data File 3). For instance, hypertension is associated with left ventricular hypertrophy and decreased strain in the basal septum. Heart failure shows widespread impairment of strain while coronary atherosclerosis was associated with regional changes in both strain^circ^ and strain^rad^. Spatial associations in structure and function were also related to conduction disorders and arrhythmias, as well as cardiometabolic diseases. Genetic variants are known to be associated with cancer therapy–induced cardiomyopathy and we found that chemotherapy was associated with regionally reduced strain without hypertrophy.^17^

### GWAS of spatial cardiac traits

**Figure 4:**
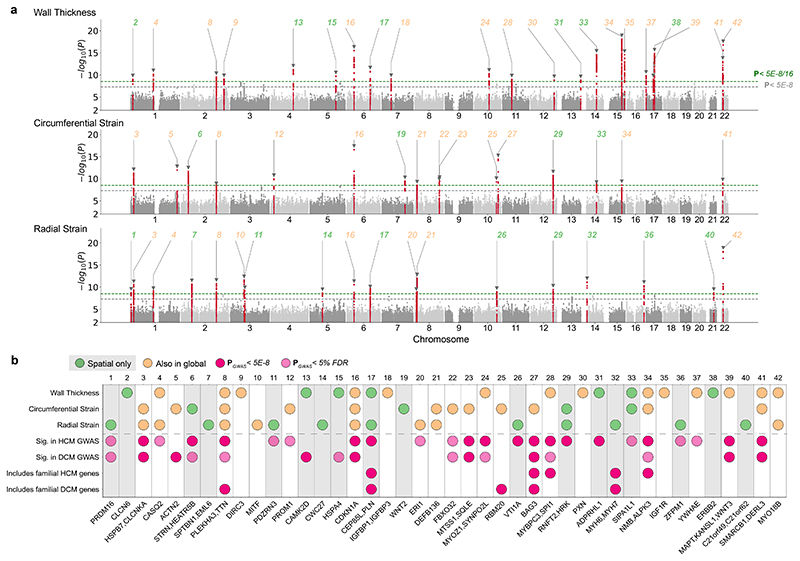
GWAS of spatially resolved wall thickness and contractility. (**a**) Manhattan plots for spatially resolved analysis on three left ventricular traits, namely wall thickness, strain^circ^ and strain^rad^. The plots show variant-based two-sided minimum *P* values from 16 GWAS of left ventricular AHA segments for the indicated traits for up to 40,186 Europeans in UK Biobank. In the merged spatial analysis, each locus was defined at above multiple-hypothesis adjusted genome-wide significance threshold of 3.125 x 10^−9^ (green dotted line) and labelled sequentially by chromosome location across all loci. The conventional genome-wide significance at 5 x 10^-8^ is shown as a grey dotted line. Spatial-only loci are labelled in bold green and those that were found significant in global traits by conventional genome-wide significance were labelled orange. Locus 8, 28, 29, and 39 contain more than one independent lead SNP (LD r^2^ < 0.1). (**b**) Locus look up in cardiomyopathy genes. The loci not significant in the corresponding globally averaged trait are highlighted with a grey background. Significance is defined as having at least one SNP in the corresponding locus window that reached conventional genome-wide significance (dark pink) or 5% FDR (light pink) for the indicated cardiac disease. Locus naming was performed primarily by gene prioritisation considering FUMA and prior gene association with Mendelian HCM or DCM. See Supplementary Table 3 for list of locus genes. Supplementary Figures 7 to 9 show beta values of lead SNPs in the spatial GWAS loci by individual segments.

We investigated which common genetic factors underlie regional structural and functional traits in the left ventricle. We used a conservative significance level (*P* < 3.125 x 10^−9^) based on the conventional genome-wide significance threshold (5 x 10^−8^) to adjust for the multiple tests that were performed for each trait spatially. At this threshold, 42 genomic loci were discovered for the three image-derived spatial traits comprising 21 loci for wall thickness, 16 for strain^circ^ and 18 for strain^rad^ (Figure 4a and Supplementary Table 3). Gene prioritisation (Supplementary Data File 4) was performed by considering the nearest gene, and tissue specific annotations by expression quantitative trait loci (eQTL) and chromatin interactions (CI) in the left ventricle, aorta, and other artery-related tissues using FUMA.^18^ The majority (>90%) of genes or gene products were annotated by location and the left ventricle specific eQTL (Supplementary Figure 6). Mendelian cardiac genes in the GWAS loci were mapped using the Cardiac Genotype-to-Phenotype (CardiacG2P) database (Supplementary Table 4).^19^ Eighteen out of the 42 loci identified with spatial phenotyping would not have been discovered through GWAS of conventional global traits of mass and volume (including global mean/maximum wall thickness, global mean strain^circ^ and strain^rad^, left ventricular mass and ejection fraction) (Figure 4a). Among the 18 loci we found 11 that overlapped with either a hypertrophic or dilated cardiomyopathy locus (Figure 4b).^15,16^ Specifically, these include 6 loci (*STRN, CAMK2D, PLN, VTI1A, TBX3, ADPRHL1*) that reached a threshold (*P* < 5 x 10^−8^) in HCM or DCM GWAS, and an additional 5 loci (*PRDM16, PDZRN3, HSPA4, SIPA1L1, ZFPM1*) that reached a 5% FDR cutoff. One additional locus was mapped to the sarcomeric gene *MYH6*, which encodes the alpha heavy chain subunit of cardiac myosin and is linked to an electrocardiogram GWAS^20^ and congenital heart disease (ClinGen, CCID:008375). Two genes in these loci were linked to Mendelian cardiomyopathies, including *PLN* and *MYH7*, where *MYH7* was mapped through artery aorta eQTL (Supplementary Table 4). The remaining 6 spatial-only loci, that have not yet been found to be directly associated with HCM or DCM, contained genes or gene products related to myocardial wall stress and vascular smooth muscle contractility (*CLCN6*^21,22^), cardiomyocyte differentiation and cardiac development (*SPTBN1*^23^, *WNT2*^24^) and heart failure (*STARD3*^25^). The spatial loci lead SNPs show non-uniform patterns of spatial association to left ventricular wall thickness and contractility (Supplementary Figures 7 to 9). Together, these show that a spatially-resolved GWAS provides additional power to identify genomic variants that are disease relevant.

### Transcriptome-wide association study (TWAS) for gene prioritisation

**Figure 5:**
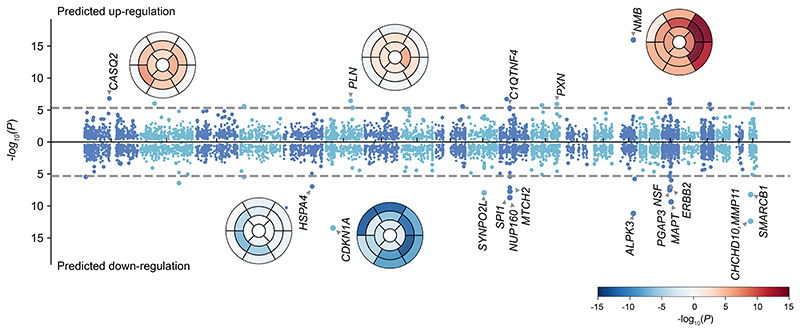
Predicted regulatory effects of GWAS variants on expression. Regulatory effects were calculated using GWAS summary statistics and GTEx v8 eQTL MASH-R model for the “heart left ventricle”. 10,498 genes were tested. Manhattan plot shows the minimum *P* value for segmental wall thickness, with chromosomes coloured by alternate dark and light blue. Bullseye plots were colored with red (positive effect of predicted gene expression on wall thickness) and blue (negative effect of predicted gene expression on wall thickness). eTWAS plots for strain^circ^, strain^rad^ are shown in Supplementary Figure 10.

Cis-eQTL variants significantly associated with protein expression in left ventricular tissue (GTEx v8)^26^ were mapped in 17 of the 42 GWAS loci. We therefore performed eQTL transcriptome wide association analysis (TWAS) to assess the potential downstream regulatory effects of GWAS variants on expression. TWAS was performed on regional GWAS summary statistics using the MetaXcan framework^27^ and the GTEx v.8 eQTL MASHR-M models (http://predictdb.org/).^26^ We used Bonferroni correction for the number of genes tested to identify significant gene associations. TWAS results are reported for regional wall thickness (Figure 5), strain^circ^ and strain^rad^ (Supplementary Figure 10).

We found common variants associated with increased wall thickness and strain rates correspond to significant down-regulation of expression for *CDKN1A, SYNPO2L, ALPK3, MAPT, MMP11* and *SMARCB1*, and to significant up-regulation for *CLCNKA, CASQ2, HSPB7, NMB*. We also observed regional patterns of significance for known global loci with examples (*CASQ2, CDKN1A, NMB, ALPK3*) shown as bullseye plots (Figure 5).

### Variant level exome-wide association study (ExWAS)

**Figure 6:**
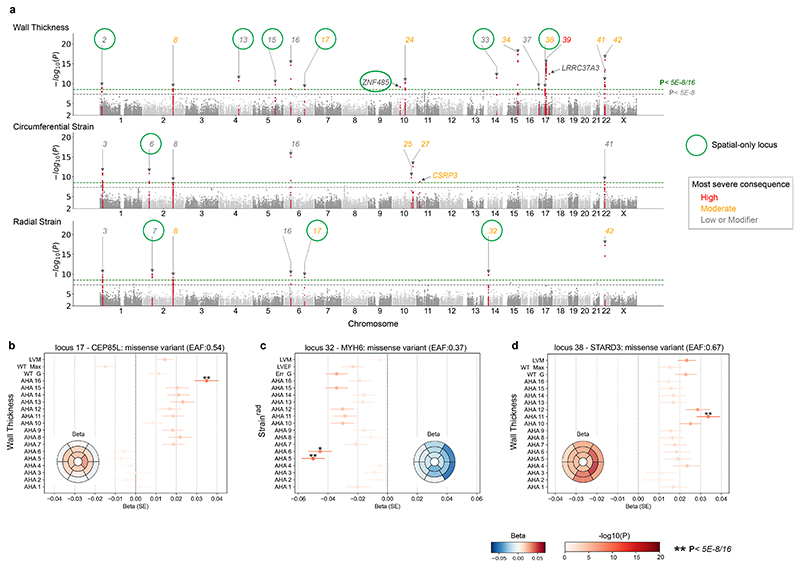
Exome-wide variant-level association study (ExWAS) on spatially resolved wall thickness and contractility. (**a**) Manhattan plot of ExWAS for spatial traits. The smallest *P* values across 16 segments were plotted for wall thickness, strain^circ^ and strain^rad^. Locus definitions were taken from the GWAS analysis. Three exome variants that reached significance but not mapped to the 42 GWAS loci are labeled with the gene names. The loci are coloured by the most severe consequence of the variants. Spatial only loci from the GWAS analysis are circled in green. (**b**) Forest plots to show the association beta and *P* value for selected variants on spatial wall thickness, global mean wall thickness (WT G), maximum wall thickness (WT Max) and left ventricular mass (LVM). Forest plots are coloured by log-scaled *P* values and significant ones are labeled with asterisks. The insets are bullseye plots of beta values. Direction of beta values are specific to the effect alleles. The variants plotted are (in GRCh38): chr6:118566140:T:C (CEP85L), chr14:23392602:A:G (MYH6), chr17:39657827:G:A (STARD3), from left to right.

Using the whole exome sequencing (WES) data, we tested variant-level association for those not included in the imputed genomes, and for rare variants with ≥7.8 x 10^-5^ minor allele frequency (MAF) in the CMR cohort. After quality control, 1,006,431 exome variants were included, 73.8% had a MAF < 0.001 and about 25% had an Ensembl impact level of high or moderate (Supplementary Figure 11). At the conservative threshold of *P* < 3.125 x 10^-9^, 655 significant genotype-to-spatial trait associations from 154 exome variants were identified, of which 82 associations were from 19 exome variants not identified at GWAS (listed in Supplementary Table 5). Overall, ExWAS identified variants in 20 of the 42 GWAS loci, and 3 additional variants outside the GWAS loci (Figure 6). An example of these additional variants was a rare missense variant in *CSRP3* (rs45550635, MAF 0.5%, missense W4R), which associated with increased strain^circ^ and left ventricular ejection fraction. *CSRP3* is a cardiomyopathy-associated gene implicated primarily with HCM, but has not been discovered in previous GWAS. These ExWAS summary statistics were combined with GWAS results to provide a comprehensive interpretation of associated variants.

We analysed ExWAS variants within the 42 loci identified in the spatial left ventricular trait GWAS. There were 6,631 exome variants tested in the 42 loci, of which 5,683 were not included in the imputation set GWAS was performed on. We found that 87.1% of these exome-only variants were rare (MAF < 0.1%), and 96.3% of those with high impact were also rare (truncating, splice donor or splice acceptor) (Supplementary Figure 11f). These mostly rare and high impact exome variants were observed in 14 GWAS loci, of which 10 were also HCM or DCM loci that include stop gained variants in genes (*CLCNKA, TTN, CEP85L, PRAG1, AGAP5, SYNPO2L, ADPRHL1, ALPK3, LRRC37A2, SMARCB1*), listed in full in Supplementary Table 6. The majority of these variants were very rare (at the limit of observed at least five times in the cohort, MAF < 0.01%), and had a larger effect size than GWAS significant lead variants. This catalogues functionally significant variants in reported cardiomyopathy-related genes observed in a population that excluded those with diagnosed cardiomyopathy.

A spatial approach to phenotyping identified loci containing Mendelian genes implicated in cardiac diseases that did not reach significance using global traits (Figure 6). In the *CEP85L*/*PLN* locus, the sentinel variant (rs3734381) has been identified from previous HCM and QRS duration GWAS^28^ but has never been identified for left ventricular wall thickness or mass. Here, we found this variant to be associated with spatially resolved wall thickness with an apical to basal gradient (Figure 6b). In addition, a missense variant in *MYH6* (rs365990) was not found associated with left ventricular mass, ejection fraction or global strain^rad^, however, it has a spatial strain^rad^ association in the basal lateral wall (*β* = -0.05, *P* < 3.125×10^−9^, Figure 6c).

### Exome-wide gene-based tests on rare deleterious variants

We further performed gene-level burden tests to assess the collective effect of rare loss-of-function or deleterious variants which were too rare to be tested at variant level. Using masks provided by UK Biobank^29^, up to 9,865 genes were tested for predicted loss-of-function (pLoF) variants, and 15,615 genes for deleterious variants using Regenie^30^ (see Methods for details). Variance component tests that takes into account the directionality of effects were also performed on up to 3,666 genes for pLoF variants, and up to 9,732 for deleterious variants. For spatial traits, *P* values of genes tested on all 16 segments by the same test were merged to assess false discovery, and a significance level threshold was set at 5% FDR (q value < 0.05). Supplementary Data File 5 shows full list of genes that passed this threshold.

For genes harbouring rare (MAF < 0.01) pLoF variants, *EXD2* and *MYOT* (*P* < 1.4 ×10^−5^) significantly associated with left ventricular mass, *EXD2* and *PCDHA12* (*P* < 7.8×10^−6^) with global mean wall thickness, and *TTN* with ejection fraction, radial and circumferential strains. Carriers of *EXD2* rare pLoF variants had decreased left ventricular mass and wall thickness (*β* =-1.1, -0.9 respectively). *TTN* is a definitive-evidence dilated cardiomyopathy gene where truncating variants are associated with cardiac phenotypes.^31^ We also identified *ALPK3* as having a spatial wall thickness association through variance component tests (SKAT, *P* = 9.28 x 10^-8^) that takes into account different directions of effect from pLoF variants (Supplementary Figure 12a). The LOVO tests showed the direction of effect for each pLoF variant with respect to changes in wall thickness (Supplementary Figure 13).

For genes harbouring rare predicted deleterious variants, we found an association between *INHBB* with spatial wall thickness, and *CSRP3* with spatial and global contractility (Supplementary Figure 12b). *CSRP3* and *TTN* displayed opposite directions of association with contractility: the deleterious variants of CSRP3 were associated with increased strain^circ^ while *TTN* pLoF variants were associated with decreased strain^circ^. *INHBB* deleterious variants (0.014% of cohort) showed a protective role against increased wall thickness (*β* =−1.1, *P* = 1.84 x 10^-6^). We also found an association of *AQP1* with spatial wall thickness by the variance component tests (SKAT, *P* = 3.90 x 10^-7^). *AQP1*, aquaporin 1, is a water channel protein differentially expressed in cardiovascular tissues where it regulates transmembrane homeostasis and is also associated with cardiovascular outcomes.^32,33^

### Causal association with haemodynamics

**Figure 7:**
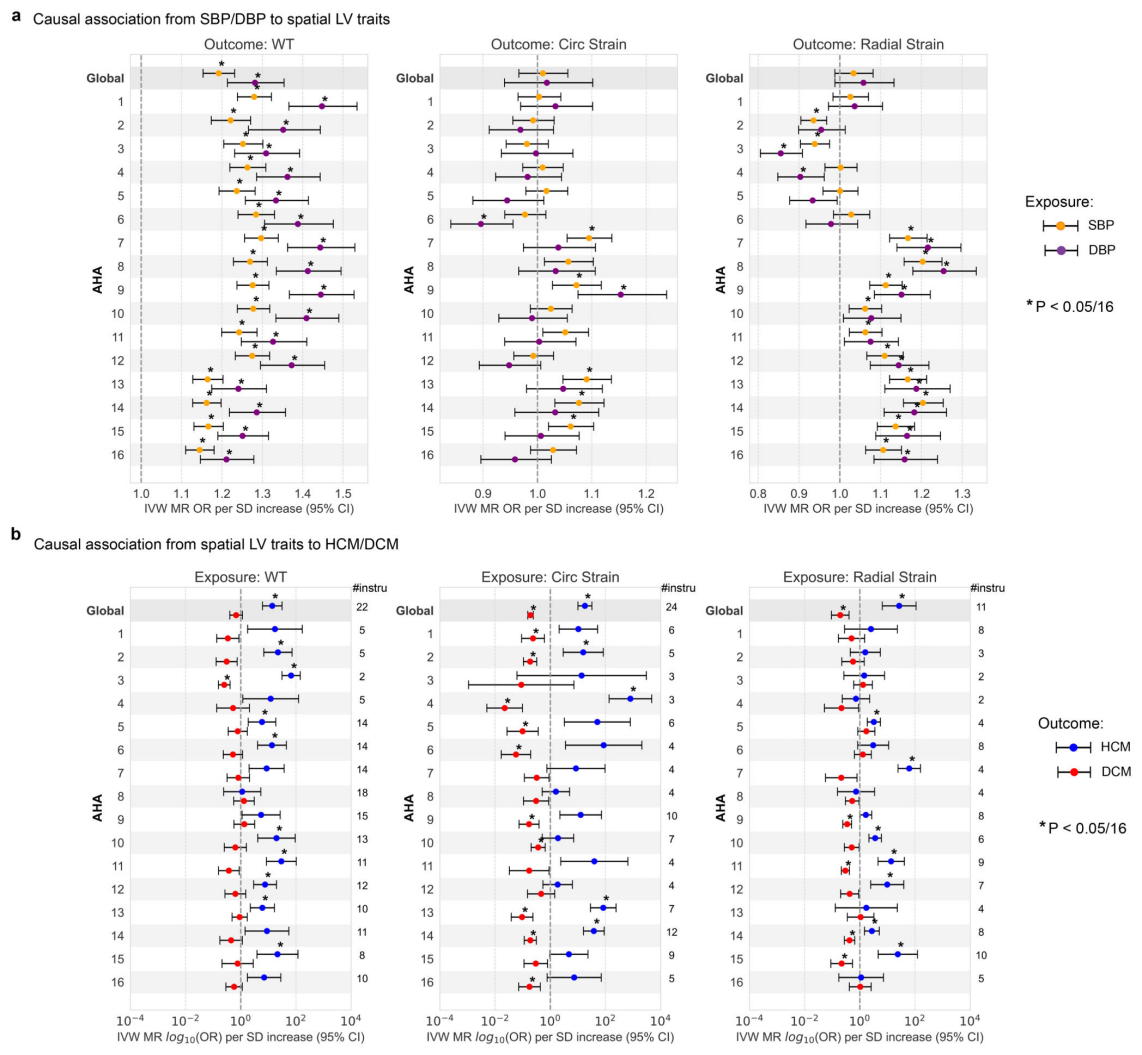
Mendelian randomization (MR) analysis of blood pressure on spatial traits, and spatial traits on the risk of HCM and DCM. Odds ratio (OR) represented are those inferred from the inverse variance weighted (IVW) two-sample MR per standard deviation increase (SD). The error bars represent the 95% confidence interval of the odds ratio (OR). OR for circumferential strain reflect those of increased contractility. Asterisk indicates the MR IVW test *P* value is below multiple hypothesis adjusted threshold (*P* < 0.05/16). (a) MR results on increased SBP/DBP on risk of increased spatial spatial traits, including the global mean (G) and regional mean on AHA segments (1-16) for wall thickness, strain^circ^ and strain^rad^. Spatial traits used GWAS on rank-based inverse transformed (rIVT) adjusted with Sex, age, BMI and BSA at MRI, in 40,058 participants of the UKB without cardiomyopathy and with available CMR. Genetic instruments for SBP, DBP were selected from a published GWAS including up to 801,644 individuals.^13^ (b) MR results on increased global and spatial left ventricular wall thickness, strain in circumferential and radial directions on risk of HCM and DCM. Genetic instruments for spatial traits were selected from the present GWAS and the number of SNPs involved in performing MR to HCM are listed on the right side of each panel. The outcome HCM GWAS included 5,927 cases vs. 68,359 controls^15^, DCM included 14,255 cases vs. 1,199,156 controls.^16^

Both SBP and DBP have an established causal influence on HCM,^6,15^ and also mediate elevated left ventricular mass in the general population,^34^ although the patterns of remodelling may be distinct in these groups.^5^ Mendelian randomisation (MR) uses genetic variants that are robustly associated with a complext trait, to generate unbiased detection of causal effects. The approach is analogous to the randomized controlled trial (RCT), but with genetic variants as proxies^35^. Here, we performed MR between haemodynamic factors and each regional trait of the myocardial wall. We used MR Egger to test for causality as this method accounts for pleiotropic effects. Both SBP (Egger, *P* = 2.82 x 10^-12^) and DBP (Egger, *P* = 1.65 x 10^-8^) have a causal relationship with mean wall thickness, where DBP has a higher effect ratio (Supplementary Figure 14). We further performed the MR analysis of BP on spatial traits and found significant associations at adjusted *P* values (Egger, *P* < 0.003125), and performed reverse MR using spatial traits as exposures and BP as the outcome where no significant reverse causality was found. We used the inverse variance weighted test (IVW) to estimate the magnitude of causal association, and observed a gradient of stronger odds ratio of wall thickness increase per standard deviation change in BP from base to apex, for both SBP and DBP (Figure 7a). This shows a causal relationship between BP and left ventricular geometry.

A causal effect of SBP and DBP on myocardial strain was observed regionally even though there was no causal effect on global mean strain^circ^ or strain^rad^ (Figure 7b,c). Higher SBP and DBP was causally linked to increased strain^rad^ in mid antero-septal segments (AHA 7,8, IVW, *P* = 5 x 10^-5^, 1 x 10^-6^ for SBP, *P* = 1 x 10^-4^, 8 x 10^-5^ for DBP), while the direction of effect was opposite for inferior basal segments. For spatial strain^circ^, increased SBP and DBP are related to decreased strain in basal-lateral segments and increased strain in mid-inferior septal segments, although the causal effect is insignificant.

### Causal association of spatial traits and cardiomyopathy

Prior data from GWAS and MR support a causal role of increased global left ventricular contractility in both obstructive and non-obstructive forms of HCM.^15^ We performed bidirectional Two-Sample MR tests between spatial LV traits and HCM, DCM risks (Supplementary Data File 6). Egger’s test showed that contractility was causally associated with DCM, but not with HCM. We found that the IVW test supported a causal role of global and regional mean wall thickness for HCM, as well as global and regional myocardial strain for HCM and DCM risk (Figure 15b). Egger’s test did not provide additional evidence for a causal role of mean wall thickness on HCM or DCM risk, and the high intercept suggested pleiotropic effects (Supplementary Figure 15).

### Pathways associated with remodelling

**Figure 8:**
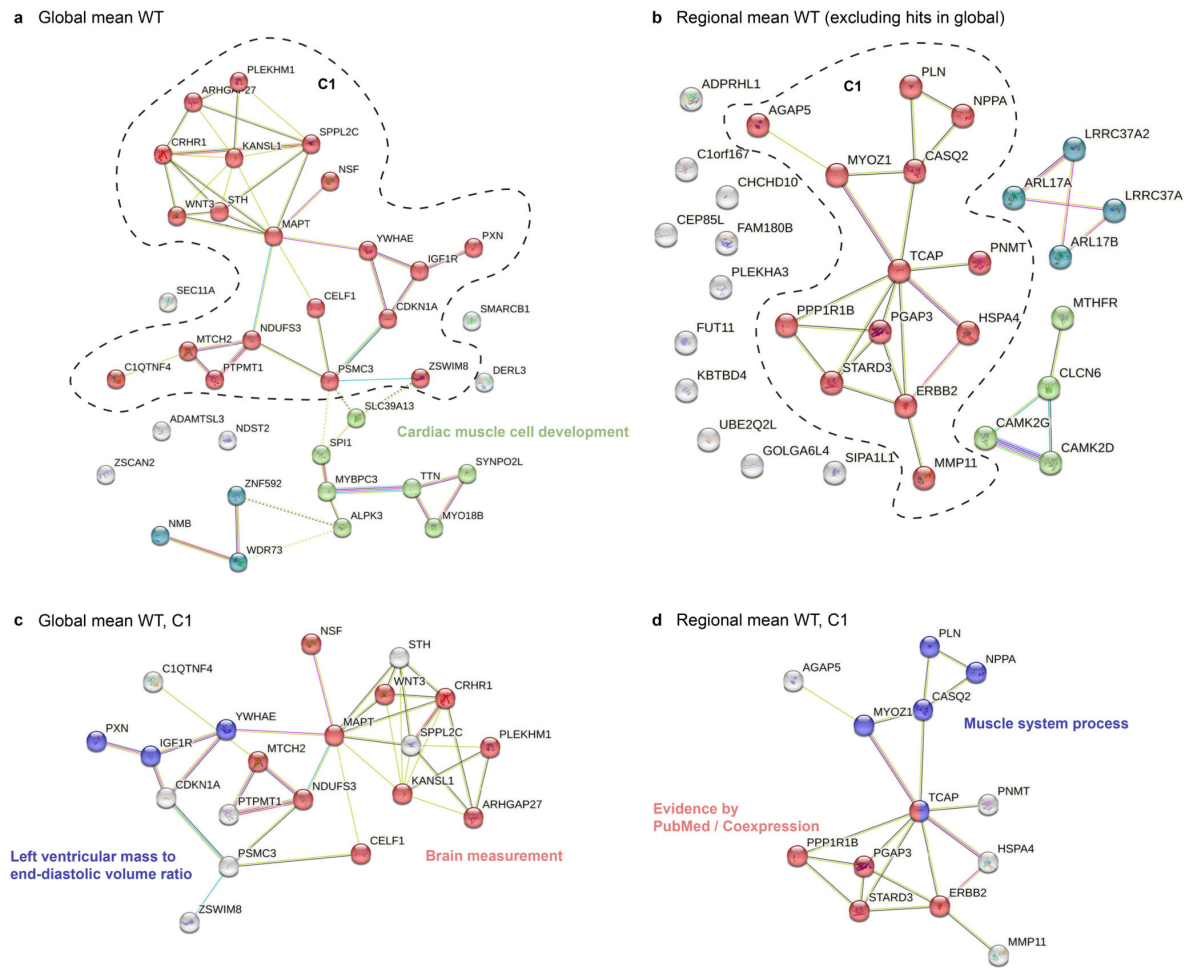
Cluster groups and gene interactions for left ventricular wall thickness. (**a**) Genes in the global mean wall thickness GWAS loci were clustered by the STRING-DB. Enrichment analysis on the largest cluster is shown in (**c**). Edges show protein–protein relationships where a protein works together to perform a common task. (**b**) Genes in the spatial-only loci of regional wall thickness were clustered by the STRING-DB. Enrichment analysis on the largest cluster was shown in (**d**). Light blue line: known interactions from curated datasets; purple line: experimentally determined known interactions; dark blue line: predicted interactions from gene neighborhood; red line: predicted interactions from gene fusions; dark blue line: predicted interactions from gene co-occurrences; light green line: predicted interaction from text mining; black line: co-expression; light purple: gene homology.

We next identified potential interactions among protein-coding genes associated with regional traits. We performed unsupervised gene clustering and pathway enrichment analysis on the interaction network using the STRING-DB server (Supplementary Data File 7).^36^ Protein-coding genes that were significant in global mean wall thickness were clustered into three groups (Figure 8a). The largest cluster contained 10 genes (*WNT3, NDUFS3, MTCH2, MAPT, NSF, CRHR1, KANSL1, PLEKHM1, ARHGAP27, CELF1*) that were significantly enriched for brain volume and cortical area, and 3 genes (*PXN, IGF1R, YWHAE*) with enrichment for left ventricular mass to end-diastolic volume ratio. These genes together also constitute a significant enrichment of anthropometric measurements (Figure 8c). The other two clusters have enrichment in heart and muscle development (*ALPK3, SYNPO2L, MYO18B, MYBPC3, TTN*), and reported GWAS on image-derived traits (*NMB, WDR73, ZNF592*).^7^ The enrichment analysis of mean wall thickness GWAS-identified genes showed processes that are known to affect global development of the left ventricular wall. The re-discovery of genes underlying brain and anthropometric measurement shows genetic components that underlie muscle and size development have shared influence across human organs.

Protein-coding genes that were significant in spatial-only loci for wall thickness were clustered into three main groups with 11 genes un-clustered, suggesting the pathway information behind the spatial-only hits is less understood (Figure 8b). The largest cluster identified was enriched for muscle system process genes and a cluster centered on *STARD3* (Figure 8d). The StAR Related Lipid Transfer Domain Containing 3 (*STARD3*) gene was reported to contain deleterious or damaging protein-coding variants for heart failure with reduced ejection fraction.^25^

We further included genes in spatial-only loci for strain^circ^ and strain^rad^ in the analysis. These include 84 protein-coding genes identified from the 18 spatial-only loci, and STRING analysis identified 7 clusters (Supplementary Figure 16). Twenty two genes constitute the largest connected cluster, involved in the sarcomere and heart development (*MYH6, MYH7, PLN, ERBB2, WNT2, TCAP*), and the ERBB2 pathway (*STAT5A, STAT5B, HSPA4, NPPA*). The other clusters identified genes involved in the regulation of excitation contraction coupling in heart and voltage-gated chloride channels (*CAMK2D, IFNGR2, UQCRQ*), genes associated with splicing regulation (*SREK1IP1, CWC27, PPWD1*), genetics associated with cardiac QRS duration and cardiac ventricular conduction (*HEATR5B, STRN*).^28^ This analysis provides an extended view of genes and pathways involved in the regulation of cardiac physiology.

## Discussion

The three dimensional structure of the heart in health and disease shows high endophenotypic diversity which is predictive of outcomes.^5,37,38^ Here, we aimed to discover the genetic determinants of patterns of structural and functional adaptation in the heart through spatially-resolved phenotyping and genetic association analyses. Using deep learning derived anatomic atlases we found spatially-distinct associations in loci related to cardiac chamber development and stress-response pathways. We also discover novel associated loci not detected using global traits, identify potential pathways that regulate phenotypic adaptation, and dissect the causal relationships between environmental stimuli and remodelling.

A spatially-resolved GWAS of left ventricular traits enables the discovery of genetic associations that have regional expression. Newly discovered loci include several that are associated with cardiomyopathies in case-control GWAS for HCM (*CEP85L/PLN, ADPRHL1, SIPA1L1*) and DCM (*HSPA4, CAMK2D*), as well as the sarcomeric gene *MYH6*, which is linked to congenital heart disease but has not previously been associated in left ventricular global trait GWAS. Spatial contractile function reveals 2 loci (*STRN, PRDM16*) that are also found in cardiomyopathy GWAS. *STARD3* is not related to cardiomyopathy but is associated with heart failure. *PLN* and *MYH7* (eQTL hit in the *MYH6* locus) were both reported as pathogenic genes in hypertrophic cardiomyopathy.^39^ These findings extend the known repertoire of shared genetic loci between cardiac development and adaptation in adults with GWAS and Mendelian cardiomyopathy-associated genes. Exome-wide analyses discovered a further 3 loci including a missense variant in *CSRP3* (amino acid mutation W4R) which was analysed in cardiomyopathy Mendelian studies but not yet reported in GWAS catalog, highlighting the additional power for variant-level discoveries against using impute-only data. Gene-level burden tests looked at the effect of ultra-rare pLoF variants. Rare loss-of-function variants in the cardiomyopathy gene *ALPK3*, which regulates cardiomyocyte and myofibroblast differentiation,^40,41^ were associated with increased regional wall thickness. *ALPK3* is one of several reported genes that have a recessive association with cardiomyopathy supporting a semi-dominant model of inheritance.^42^ We found that common missense variants in *ALPK3* are associated with decreased wall thickness, whereas the pLoF variants are together associated with hypertrophy suggesting a potentially protective role for GWAS variants against hypertrophy. Similarly, the sentinel missense variant in *MYH6* was found to be associated with decreased basal wall thickness, whereas predicted damaging variants are associated with increased wall thickness in cardiomyopathy.^43^

Blood pressure has a key causal influence on global left ventricular hypertrophy with early involvement of the basal septum.^9,44^ Non-sarcomeric HCM is also characterised as a polygenic hypertrophic sensitivity to DBP.^6^ We showed there was a spatially-varying gradient of causal association between blood pressure and wall thickness from base to apex with DBP having a stronger effect. While blood pressure is the causal exposure the hypertrophic response depends on wall stress which is highly heterogeneous in the left ventricle and greatest where the septum is flattest near the outflow tract.^45^ We also found a genetic correlation between physiological variation in hypertrophy and inherited cardiomyopathies - with opposite directions of effect in HCM and DCM. Causal analyses show how common genetic variation modifies Mendelian disease risk and morphofunctional expression contributing to the diversity of cardiomyopathy phenotypes.

Using unsupervised gene clustering and pathway enrichment analysis we found 10 genes that share an enrichment for brain volume and cortical area. There is a shared genetic influence between markers of heart and brain health,^46^ and our findings suggest some genes may contribute to multiorgan development and the emergence of complex traits. Genes discovered only through spatial phenotyping revealed three main groups with the largest regulating heart regeneration and trabecular development through promotion of cardiomyocyte de-differentiation and proliferation.^47,48^ The NRG-1/ErbB signaling pathway is involved in diverse aspects of cardiomyocyte biology has also been identified as a therapeutic target for cardiomyopathy and even heart regeneration.^49^ Pathway analysis also identified genes involved in cardiac conduction suggesting that ion channel genes are critical for regulating the contractility of the adult heart as well as formation of the conduction system.^50^

### Clinical implications

This work provides a comprehensive resource for understanding the genetic associations of left ventricular morphology and contractile function as well as the response to stress. This could inform understanding of new monogenic causes of hypertrophy as well as polygenic modifiers of disease phenotypes. The pathways discovered in this work could also be investigated as potentially druggable targets for modifying adverse changes in ventricular remodelling.

### Limitations

UKB is a prospective cohort study with deep genetic and phenotypic data that is unique in its size and scope.^51^ However, certain age groups and persons living in less socioeconomically deprived areas are under-represented.^52^ The population is predominantly European (95%) and further research is required in people of diverse ancestries and social groups. Cross-ancestry analyses may help in finding new associations as well as improve the precision of fine mapping. UKB may show latent population stratification, however risk factor associations appear broadly generalisable.^53^ We used validated computer vision approaches to assess 48 spatial traits in up to 40,058 healthy Caucasian individuals describing the landscape of genetic architecture influencing heart structure. Through exome-wide GWAS, we extended genetic discovery beyond common variants, identifying rare variants not included in the imputed genotyping data. The use of independent disease-specific cohorts for MR enhances generalizability, and reduces the risk of population stratification bias in causal analysis. However, the study is restricted to Caucasian individuals, which limits the translatability of findings to other ancestral groups. Horizontal pleiotropy may still cause bias in causal estimates using MR. The power of exome based burden tests is also dependent on the loss-of-function variants classification and aggregation strategy. Rare variant analysis has limited statistical power and will require replication with large independent datasets if they become available.

## Conclusion

Together, the data reported here, combining advanced three dimensional human imaging with genetic analyses across the allele frequency spectrum, highlight the role that cardiomyopathy-associated genes have on the regulation of spatial adaptations in those without known disease.

## Supplementary Material

005116 - Supplemental Material

Data Set 1

Data Set 2

Data Set 3

Data Set 4

Data Set 5

Data Set 6
