# New Genetic Loci Implicated in Cardiac Morphology and Function Using Three-Dimensional Population Phenotyping

**DOI:** 10.1161/CIRCGEN.124.005116

**Published:** 2025-10-07

**Authors:** Chang Lu, Kathryn A. McGurk, Sean L. Zheng, Antonio de Marvao, Paolo Inglese, Wenjia Bai, James S. Ware, Declan P. O’Regan

**Affiliations:** Medical Research Council Laboratory of Medical Sciences, Hammersmith Hospital Campus (C.L., K.A.M., S.L.Z., A.d.M., P.I., J.S.W., D.P.O.), Imperial College London, United Kingdom.; National Heart and Lung Institute (K.A.M., S.L.Z., J.S.W.), Imperial College London, United Kingdom.; Department of Computing (W.B.), Imperial College London, United Kingdom.; Department of Brain Sciences (W.B.), Imperial College London, United Kingdom.; Program in Medical and Population Genetics, The Broad Institute of Massachusetts Institute of Technology and Harvard, Cambridge, MA (K.A.M., J.S.W.).; Department of Cardiology, Imperial College Healthcare NHS Trust, London, United Kingdom (J.S.W.).; Royal Brompton and Harefield Hospitals, Guy’s and St. Thomas’ NHS Foundation Trust (S.L.Z., J.S.W.).; Department of Women and Children’s Health (A.d.M.), King’s College London, United Kingdom.; British Heart Foundation Centre of Research Excellence, School of Cardiovascular and Metabolic Medicine and Sciences (A.d.M.), King’s College London, United Kingdom.

**Keywords:** cardiomyopathies, genome-wide association study, heart ventricles, hypertrophy, ventricular dysfunction

## Abstract

**BACKGROUND:**

Cardiac remodeling occurs in the mature heart and is a cascade of adaptations in response to stress, which are primed in early life. A key question remains as to the processes that regulate the geometry and motion of the heart and how it adapts to stress.

**METHODS:**

We performed spatially resolved phenotyping using machine learning–based analysis of cardiac magnetic resonance imaging in 47 549 UK Biobank participants. We analyzed 16 left ventricular spatial phenotypes, including regional myocardial wall thickness and systolic strain in both circumferential and radial directions. In up to 40 058 participants, genetic associations across the allele frequency spectrum were assessed using genome-wide association studies with imputed genotype participants, and exome-wide association studies and gene-based burden tests using whole-exome sequencing data. We integrated transcriptomic data from the GTEx project and used pathway enrichment analyses to further interpret the biological relevance of identified loci. To investigate causal relationships, we conducted Mendelian randomization analyses to evaluate the effects of blood pressure on regional cardiac traits and the effects of these traits on cardiomyopathy risk.

**RESULTS:**

We found 42 loci associated with cardiac structure and contractility, many of which reveal patterns of spatial organization in the heart. Whole-exome sequencing revealed 3 additional variants not captured by the genome-wide association study, including a missense variant in *CSRP3* (minor allele frequency 0.5%). The majority of newly discovered loci are found in cardiomyopathy-associated genes, suggesting that they regulate spatially distinct patterns of remodeling in the left ventricle in an adult population. Our causal analysis also found regional modulation of blood pressure on cardiac wall thickness and strain.

**CONCLUSIONS:**

These findings provide a comprehensive description of the pathways that orchestrate heart development and cardiac remodeling. These data highlight the role that cardiomyopathy-associated genes have on the regulation of spatial adaptations in those without known disease.

Diverse cardiovascular cell types coordinate to form spatially organized structures of the human heart.^1^ Interactions between biophysical mechanics, morphogenesis, and cell fate during heart development shape its 3-dimensional structure.^2^ Cardiac remodeling occurs in the mature heart and is associated with the development and progression of ventricular dysfunction, which is manifest by changes in geometry and contractility that are regulated by mechanical, cellular, and genetic factors.^3^ Remodeling complicates many cardiovascular disorders and has an effect across diverse cell types and anatomic domains. During cardiac development, exposure to diverse stimuli primes gene expression profiles that drive phenotypic and functional adaptations in later life.^4^ Cardiomyopathies, in particular, are characterized by diverse morphofunctional phenotypes that relate to the additive effect of common and rare variants on the sensitivity to environmental stimuli.^5,6^ While several candidate genes have been implicated in global structural phenotypes through genome-wide association studies (GWASs),^7,8^ the genetic architecture of cardiac geometry and contractile function, as well as associations with cardiomyopathic states, remains poorly characterized.

The structure and function of the heart can be assessed through detailed mapping of wall thickness throughout the left ventricle (LV), as well as quantifying regional deformation to assess myocardial contractility. Here, we use data from over 40 000 participants in UK Biobank (UKB) with cardiac magnetic resonance imaging and apply deep learning computer vision techniques to enable spatially resolved phenotyping of LV geometry and motion (Figure 1).^9,10^ This provides a 3D time-resolved atlas of the heart to perform genetic association studies across the allele frequency spectrum. We report the effect of environmental and genetic factors on the molecular orchestration of cardiac shape, motion, and remodeling in adults. This approach shows newly associated loci only discovered through precision cardiac phenotyping, describes the regional pattern of genetic variants associated with cardiomyopathy, and reveals conserved pathways that regulate multiorgan development.

**Figure 1.**
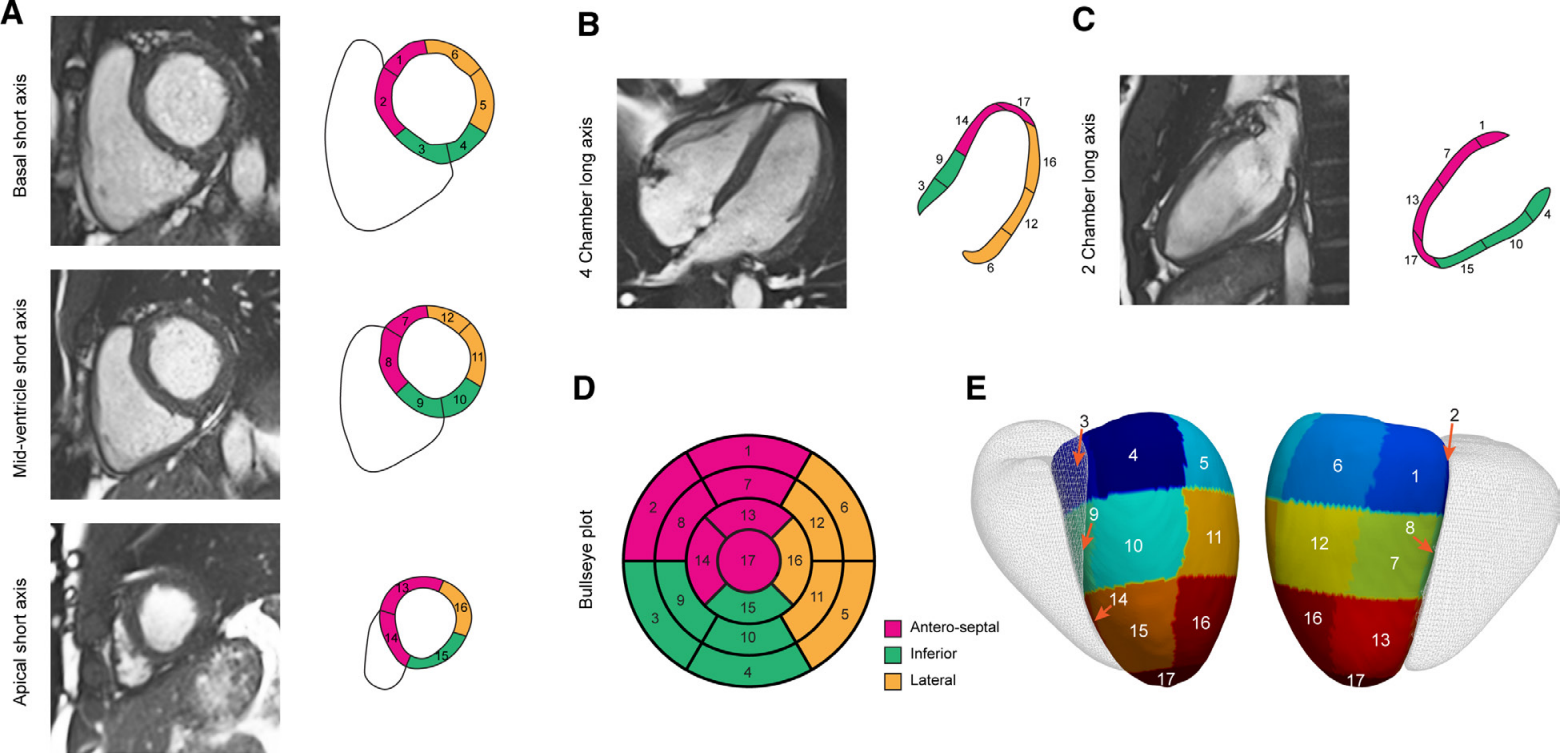
**Cardiac image analysis.** Cardiac magnetic resonance (CMR) cine imaging was performed in short and long axis planes. A fully convolutional neural network was used to segment the left ventricular myocardium and determine regional wall thickness. Motion tracking was performed using image registration to map myocardial deformation between frames and calculate regional Eulerian strain. The left ventricle was divided into 17 segments using the American Heart Association model. Short-axis (**A**), 4-chamber long-axis (**B**), and 2-chamber long-axis (**C**) CMR imaging with corresponding segments. Segments are grouped by anatomic location and numbered sequentially allowing characteristics to be represented on bullseye (**D**) and 3-dimensional (**E**) models of the left ventricle (right ventricle shown in outline).

## Methods

Full methods are available in the Supplemental Material. The code used in the study is available on GitHub (https://github.com/ImperialCollegeLondon/spatial_cardiac_GWAS). Genetic analysis was performed on the UKB Research Analysis Platform. All raw and derived data in this study are available to approved researchers from UKB (https://biobank.ndph.ox.ac.uk/), accessed under application number 40616. The National Research Ethics Service approved the UKB study (11/NW/0382). All participants gave written informed consent.

## Results

### Study Overview

We analyzed cardiac magnetic resonance imaging data from up to 47 549 participants of UKB. Deep learning segmentation and motion tracking were used to assess cardiac structure and motion throughout the LV. Data are summarized by anatomic region using the American Heart Association 17-segment model (excluding the apex). For each segment, the mean wall thickness at end diastole, the mean peak circumferential myocardial strain (strain^circ^), and the mean peak radial myocardial strain (strain^rad^) were estimated on short-axis imaging using validated pipelines.^9,11^ In addition, conventional global measures of LV mass and volume were derived.

We assessed patterns of spatial correlation of phenotypes across the LV using biophysical and hemodynamic covariates (Table S1). Phenome-wide association studies were performed on these traits after known confounders had been adjusted for. GWASs, variant-level exome-wide association studies (ExWASs), and gene-level exome-wide burden tests were performed on up to 40 058 healthy individuals of white British ancestry without cardiomyopathy for 54 spatial and global traits. We performed Mendelian randomization (MR) to analyze potential causal associations between hemodynamic factors and regional wall thickness. To understand the relationship between genetic factors regulating spatial physiology and the pathophysiology of cardiac remodeling, we performed genetic correlation and causal association analysis with hypertrophic cardiomyopathy (HCM) and dilated cardiomyopathy (DCM).

### Spatial Correlations in LV Traits

**Figure 2.**
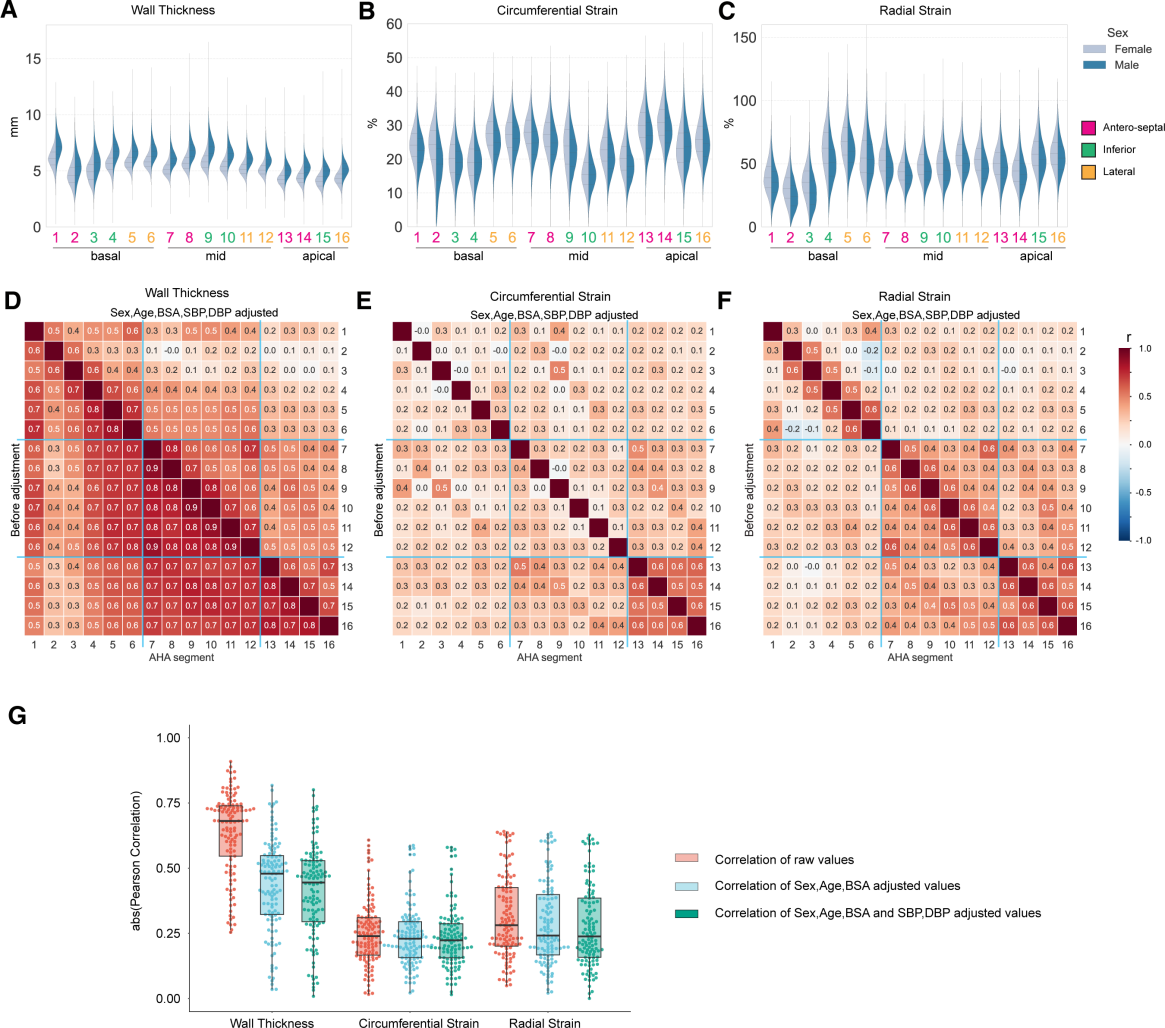
**Correlations between spatial left ventricle (LV) traits and association with known predictors. A** through **C**, Distribution of regional traits in up to 47 549 participants by sex is shown with violin plots. The Scott rule was used to determine the smoothing bandwidth for the kernel estimation. Inner dotted lines show the data quartiles. **D** through **F**, Pearson correlation among the 16 regional wall thickness (WT), strain^circ^, and strain^rad^ before (lower diagonal) and after (upper diagonal) adjustment by the indicated confounding factors. The correlation coefficients are shown as heatmaps. The color indicates a correlation efficiency from −1 to 1 (blue to red). The basal, mid-cavity, and apical segments are separated by light blue lines. **G**, Distribution of regional trait Pearson correlations before and after adjustment by the indicated confounding factors. The boxes show the quartiles of the data set, and all values are overlaid as dots.

To understand potential drivers of regional phenotypic variation in the heart, we assessed spatial correlations in wall thickness and strain across the LV. There was a strong correlation of unadjusted wall thickness between segments, apart from in the basal septum, which showed a moderate correlation (Figure 2A through 2D). Sex, body surface area, and blood pressure (BP) were significantly associated with wall thickness globally (Figure S1). After adjustment for these parameters, the spatial correlations in wall thickness were reduced to low or moderate (Figure 2G), suggesting that other unmeasured factors contribute to variation in hypertrophy.

**Figure 3.**
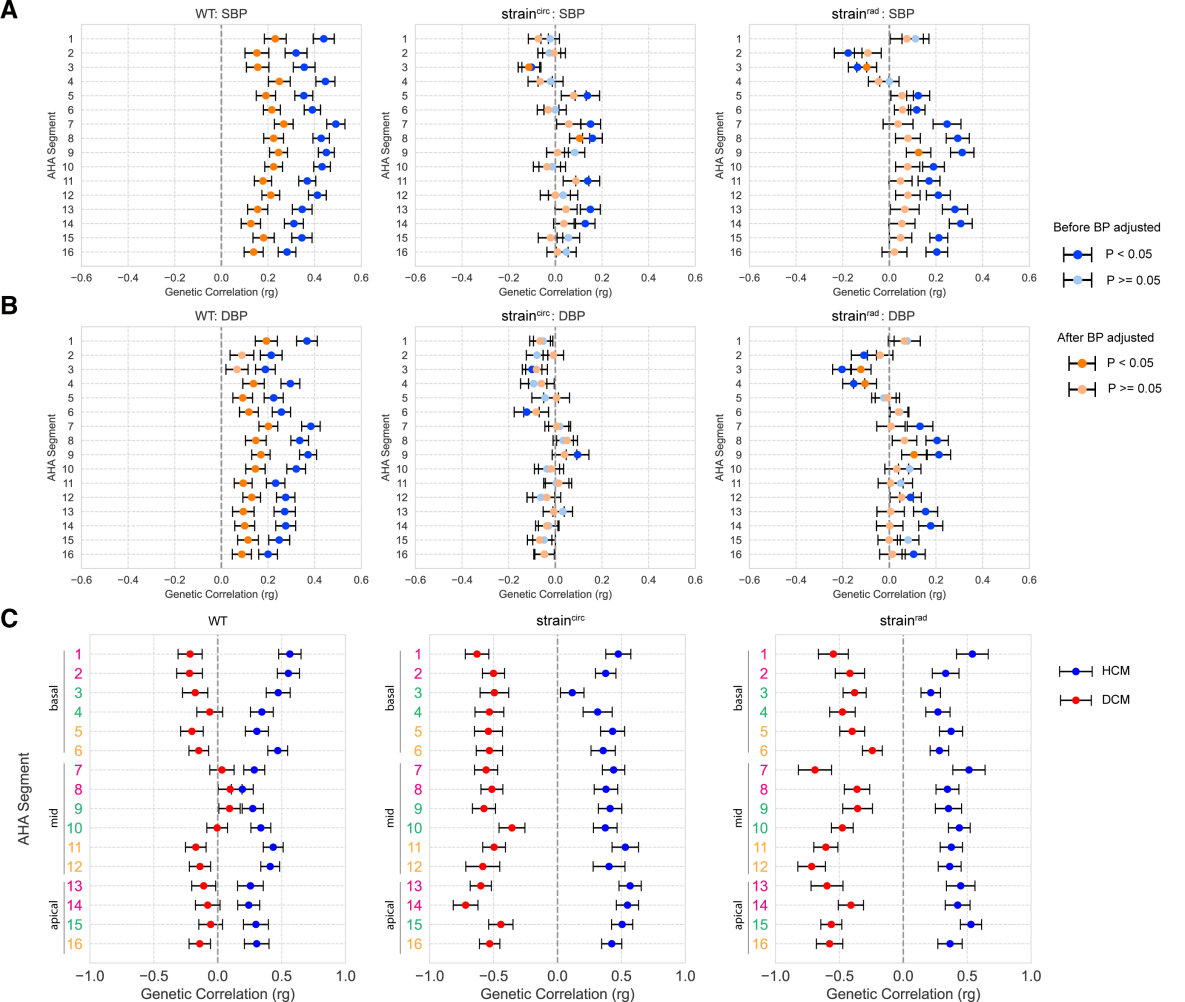
**Genetic correlation (r_g_) between spatial left ventricular traits, blood pressure (BP), and cardiomyopathies.** The r_g_ between spatial traits and both systolic BP (SBP; **A**) and diastolic BP (DBP; **B**) were calculated before (blue) and after (orange) adjustment for BP at the imaging visit. A genome-wide association study (GWAS) of spatial left ventricular traits was performed on up to 40 058 UK Biobank participants of white British ancestry. Summary statistics of SBP and DBP came from a GWAS on up to 801 644 individuals.^13^ Two-sided *P* values were estimated using linkage disequilibrium score regression. Significant correlations (*P*<0.05) are shown in darker colors. Center values are the estimated r_g_, and error bars indicate SE. **C**, r_g_ between BP-adjusted spatial traits and hypertrophic cardiomyopathy (HCM; blue) and dilated cardiomyopathy (DCM; red). Summary statistics of HCM came from GWAS of 1733 cases and 6628 controls,^15^ and DCM from GWAS of 5521 cases and 397 323 controls.^16^ AHA indicates American Heart Association; and WT, wall thickness.

Genetic correlation (r_g_) between complex traits estimates the proportion of variance that 2 traits share due to genetics, providing useful etiological insights.^12^ BP has a significant r_g_ with mid-ventricular wall thickness.^8^ Given the high phenotypic correlation between both systolic BP (SBP) and diastolic BP (DBP) with wall thickness, we compared r_g_ of BP with SBP- and DBP-adjusted wall thicknesses (Data Set S1).^13^ We found that r_g_ remained significantly associated with SBP (*P*<0.003 [r_g_, 0.13−0.27]) for wall thickness across the LV and with DBP (*P*<0.04 [r_g_, 0.09−0.20]; Figure 3A and 3B) for all regions except the basal septum, suggesting that there are shared genetic factors that regulate BP and regional wall thickness independent of a hypertrophic stimulus-response. There is also a significant degree of r_g_ between HCM and BP-adjusted spatial wall thickness (*P*<0.024 [r_g_, 0.19−0.56]; Figure 3C).

We observed weaker spatial correlations between segments for myocardial strain (Figure 2E and 2F), demonstrating more regional independence in systolic function, which would not be captured through global parameters. In contrast to wall thickness, known covariates (sex, age, body surface area, and BP) have different patterns of correlation with regional strain (Figure S1). For instance, BP is associated with decreased strain^rad^ in the basal septal wall but increased strain^rad^ in mid-ventricular and apical segments (Figure S2) and also has opposing directions of genetic correlation (Figure 3A and 3B). This heterogeneity may reflect anatomic gradients in biomechanical function related to myofibre architecture in the LV.^14^ The GREML analysis (Genome-based Restricted Maximum Likelihood) shows that myocardial strain has lower heritability (ℎ^2^, 0.06–0.24) than wall thickness (ℎ^2^, 0.14–0.30) and more unaccounted variance from known covariates and common SNPs combined (Table S2; Data Set S2). Myocardial strain also has a significant genetic correlation with both HCM and DCM across multiple LV segments (Figure 3C), highlighting shared mechanisms regulating myocyte performance in health and disease.^15,16^

Phenome-wide association studies of spatial traits show both global and anatomically localized patterns of association with 1840 phenotypes (Figures S3 through S5; Data Set S3). For instance, hypertension is associated with LV hypertrophy and decreased strain in the basal septum. Heart failure shows widespread impairment of strain, while coronary atherosclerosis was associated with regional changes in both strain^circ^ and strain^rad^. Spatial associations in structure and function were also related to conduction disorders and arrhythmias, as well as cardiometabolic diseases. Genetic variants are known to be associated with cancer therapy–induced cardiomyopathy, and we found that chemotherapy was associated with regionally reduced strain without hypertrophy.^17^

### GWAS of Spatial Cardiac Traits

**Figure 4.**
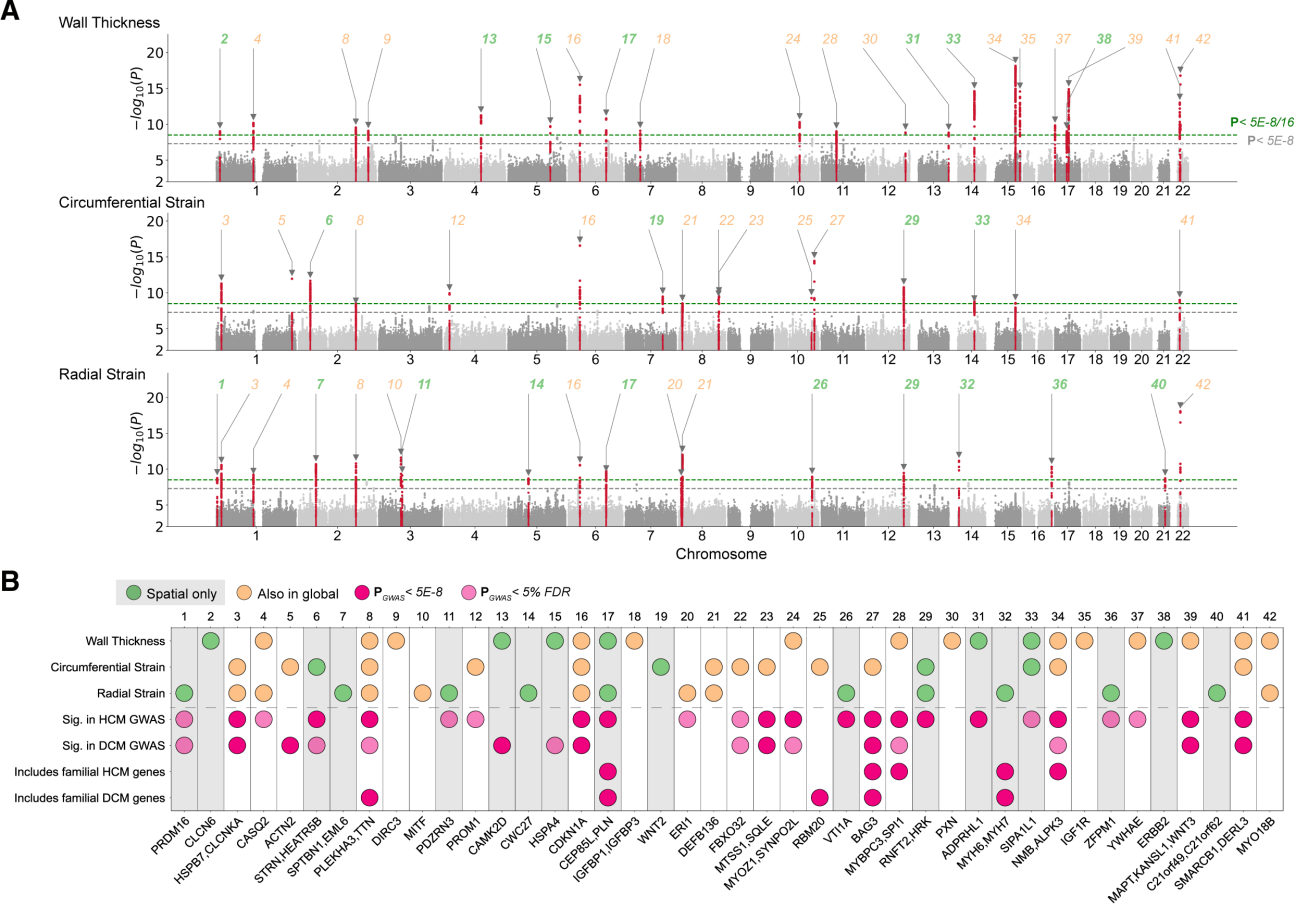
**Genome-wide association study (GWAS) of spatially resolved wall thickness and contractility. A**, Manhattan plots for spatially resolved analysis on 3 left ventricular traits, namely, wall thickness, strain^circ^, and strain^rad^. The plots show variant-based 2-sided minimum *P* values from 16 GWAS of left ventricular American Heart Association (AHA) segments for the indicated traits for up to 40 186 Europeans in the UK Biobank. In the merged spatial analysis, each locus was defined at above multiple hypothesis–adjusted genome-wide significance threshold of 3.125×10^−9^ (green dotted line) and labeled sequentially by chromosome location across all loci. The conventional genome-wide significance at 5×10^−8^ is shown as a gray dotted line. Spatial-only loci are labeled in bold green, and those that were found significant in global traits by conventional genome-wide significance were labeled orange. Loci 8, 28, 29, and 39 contain >1 independent lead SNP (LD r^2^ < 0.1). **B**, Locus look up in cardiomyopathy genes. The loci not significant in the corresponding globally averaged trait are highlighted with a gray background. Significance is defined as having at least 1 SNP in the corresponding locus window that reached conventional genome-wide significance (dark pink) or 5% FDR (light pink) for the indicated cardiac disease. Locus naming was performed primarily by gene prioritization considering FUMA and prior gene association with Mendelian hypertrophic cardiomyopathy (HCM) or dilated cardiomyopathy (DCM). See Table S3 for a list of locus genes. Figures S7 through S9 show β values of lead SNPs in the spatial GWAS loci by individual segments. FDR indicates false discovery rate; FUMA, functional mapping and annotation of genome-wide association studies; LD, linkage disequilibrium; and SNP, single nucleotide polymorphism.

We investigated which common genetic factors underlie regional structural and functional traits in the LV. We used a conservative significance level (*P*<3.125×10^−9^) based on the conventional genome-wide significance threshold (5×10^−8^) to adjust for the multiple tests that were performed for each trait spatially. At this threshold, 42 genomic loci were discovered for the 3 image-derived spatial traits comprising 21 loci for wall thickness, 16 for strain^circ^, and 18 for strain^rad^ (Figure 4A; Table S3). Gene prioritization (Data Set S4) was performed by considering the nearest gene and tissue-specific annotations by expression quantitative trait loci (eQTL) and chromatin interactions in the LV, aorta, and other artery-related tissues using FUMA (Functional Mapping and Annotation of Genome Wide Association Studies).^18^ The majority (>90%) of genes or gene products were annotated by location and the LV-specific eQTL (Figure S6). Mendelian cardiac genes in the GWAS loci were mapped using the Cardiac Genotype-to-Phenotype database (Table S4).^19^ Eighteen of the 42 loci identified with spatial phenotyping would not have been discovered through GWAS of conventional global traits of mass and volume (including global mean/maximum wall thickness, global mean strain^circ^ and strain^rad^, and LV mass and ejection fraction; Figure 4A). Among the 18 loci, we found 11 that overlapped with either an HCM or DCM locus (Figure 4B).^15,16^ Specifically, these include 6 loci (*STRN*, *CAMK2D*, *PLN*, *VTI1A*, *TBX3*, and *ADPRHL1*) that reached a threshold (*P*<5×10^−8^) in HCM or DCM GWAS and an additional 5 loci (*PRDM16*, *PDZRN3*, *HSPA4*, *SIPA1L1*, and *ZFPM1*) that reached a 5% FDR cutoff. One additional locus was mapped to the sarcomeric gene *MYH6*, which encodes the alpha heavy chain subunit of cardiac myosin and is linked to an ECG GWAS^20^ and congenital heart disease (ClinGen; CCID:008375). Two genes in these loci were linked to Mendelian cardiomyopathies, including *PLN* and *MYH7*, where *MYH7* was mapped through the aorta eQTL (Table S4). The remaining 6 spatial-only loci, which have not yet been found to be directly associated with HCM or DCM, contained genes or gene products related to myocardial wall stress and vascular smooth muscle contractility (*CLCN6*^21,22^), cardiomyocyte differentiation and cardiac development (*SPTBN1*^23^ and *WNT2*^24^), and heart failure (*STARD3*^25^). The spatial loci lead SNPs show nonuniform patterns of spatial association to LV wall thickness and contractility (Figures S7 through S9). Together, these show that a spatially resolved GWAS provides additional power to identify genomic variants that are disease-relevant.

### Transcriptome-Wide Association Study for Gene Prioritization

**Figure 5.**
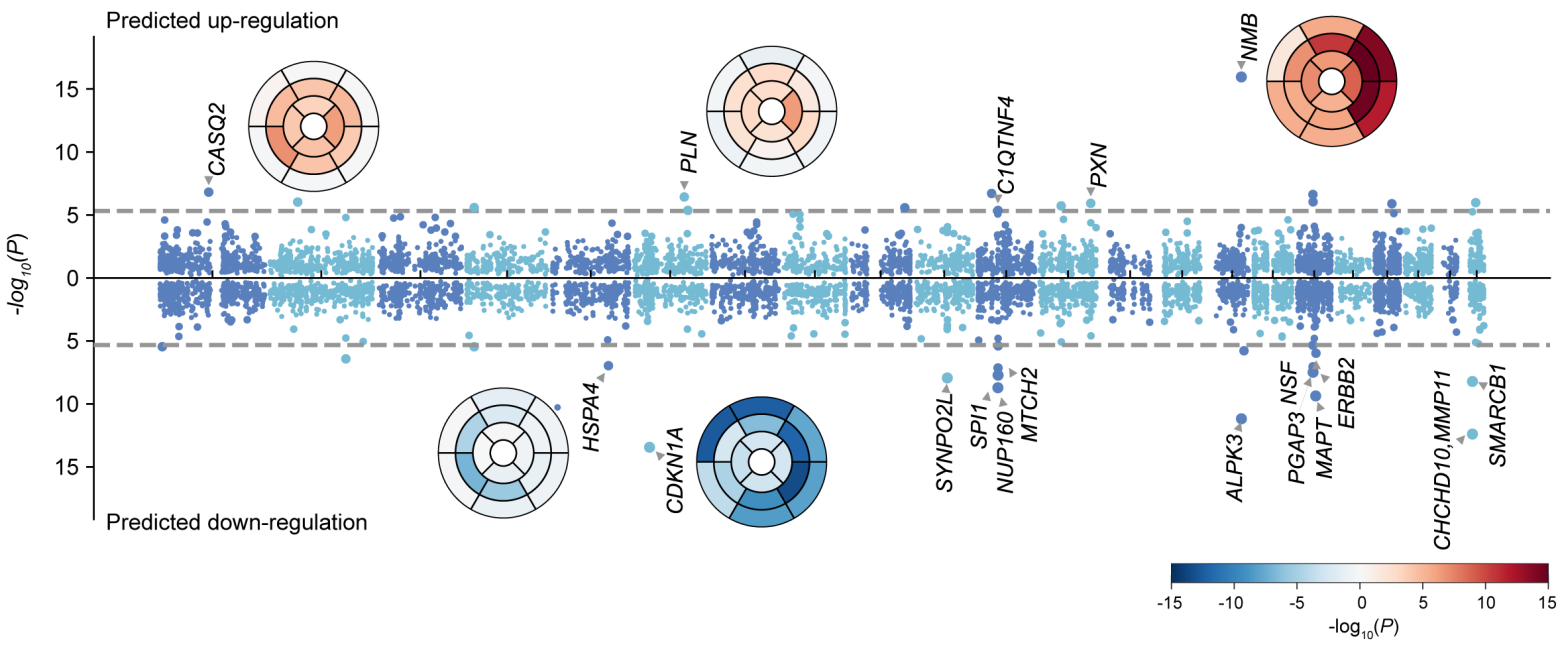
**Predicted regulatory effects of genome-wide association study (GWAS) variants on expression.** Regulatory effects were calculated using GWAS summary statistics and the GTEx, version 8, expression quantitative trait loci (eQTL) MASH-R model for the heart left ventricle; 10 498 genes were tested. The Manhattan plot shows the minimum *P* value for segmental wall thickness, with chromosomes colored by alternate dark and light blue. Bullseye plots were colored with red (positive effect of predicted gene expression on wall thickness) and blue (negative effect of predicted gene expression on wall thickness). Similarly, we performed eQTL based transcriptiome wide association studies for strain^circ^ and strain^rad^ and the results are shown in Figure S10.

Cis-eQTL variants significantly associated with protein expression in LV tissue (GTEx, version 8)^26^ were mapped in 17 of the 42 GWAS loci. We, therefore, performed an eQTL transcriptome-wide association study to assess the potential downstream regulatory effects of GWAS variants on expression. The transcriptome-wide association study was performed on regional GWAS summary statistics using the MetaXcan framework^27^ and the GTEx v.8 eQTL MASHR-M models (http://predictdb.org/).^26^ We used the Bonferroni correction for the number of genes tested to identify significant gene associations. Transcriptome-wide association study results are reported for regional wall thickness (Figure 5), and strain^circ^ and strain^rad^ (Figure S10).

We found common variants associated with increased wall thickness and strain rates correspond to significant downregulation of expression for *CDKN1A*, *SYNPO2L*, *ALPK3*, *MAPT*, *MMP11*, and *SMARCB1* and to significant upregulation for *CLCNKA*, *CASQ2*, *HSPB7*, and *NMB*. We also observed regional patterns of significance for known global loci with examples (*CASQ2*, *CDKN1A*, *NMB*, and *ALPK3*) shown as bullseye plots (Figure 5).

### Variant-Level ExWAS

**Figure 6.**
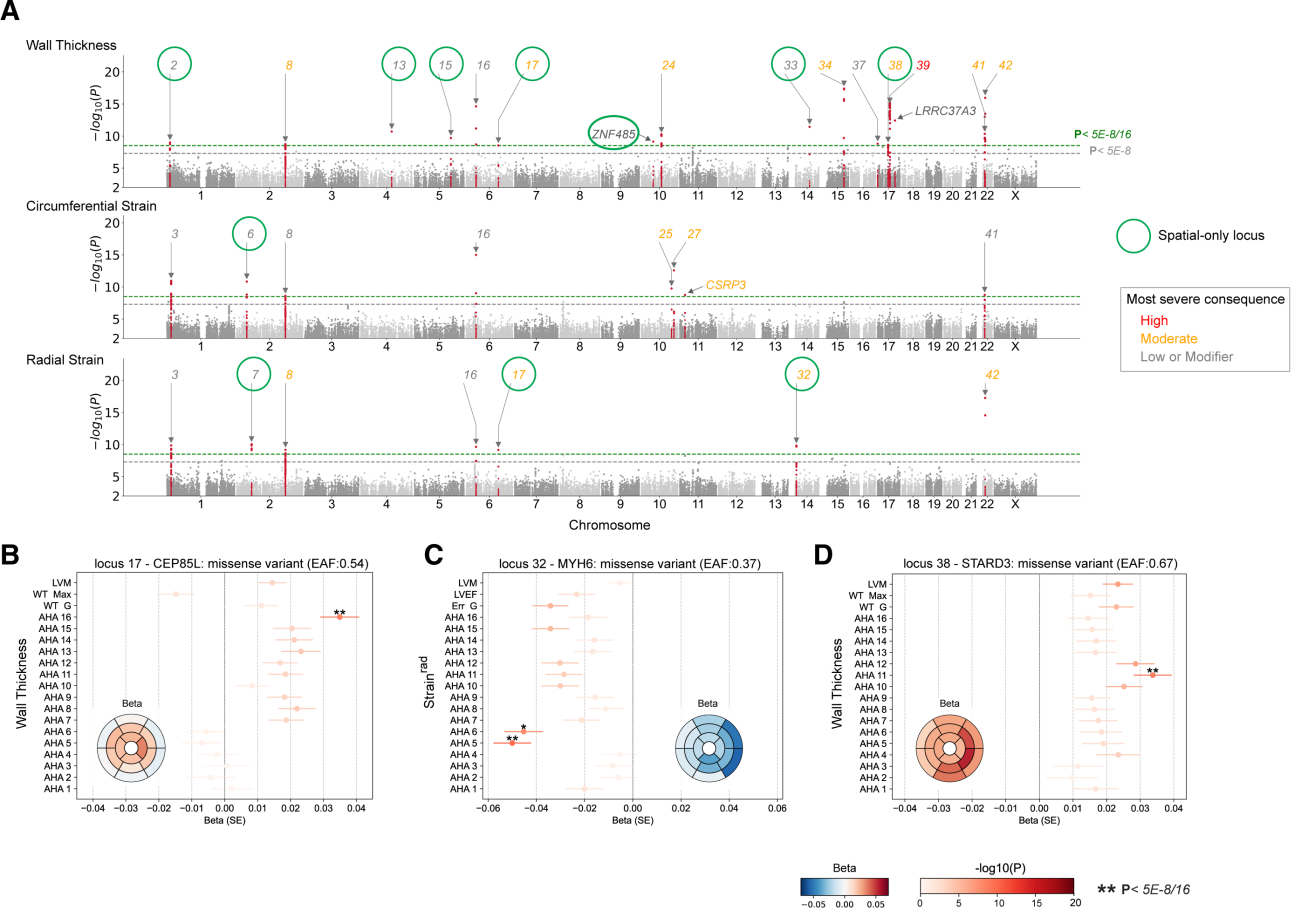
**Exome-wide variant-level association study (ExWAS) on spatially resolved wall thickness and contractility. A**, Manhattan plot of ExWAS for spatial traits. The smallest *P* values across 16 segments were plotted for wall thickness, strain^circ^, and strain^rad^. Locus definitions were taken from the genome-wide association study (GWAS) analysis. Three exome variants that reached significance but were not mapped to the 42 GWAS loci are labeled with the gene names. The loci are colored by the most severe consequence of the variants. Spatial-only loci from the GWAS analysis are circled in green. **B-D**, Forest plots to show the association β and *P* values for selected variants on spatial wall thickness, global mean wall thickness (WT G), maximum wall thickness (WT Max), and left ventricular mass (LVM). Forest plots are colored by log-scaled *P* values, and significant ones are labeled with asterisks. The insets are bullseye plots of β values. The direction of β values is specific to the effect alleles. The variants plotted are (in human genome guild 38 [GRCh38]): chr6:118566140:T:C (*CEP85L*), chr14:23392602:A:G (*MYH6*), and chr17:39657827:G:A (*STARD3*), from left to right.

Using the whole-exome sequencing data, we tested variant-level association for those not included in the imputed genomes and for rare variants with ≥7.8×10^−5^ minor allele frequency (MAF) in the cardiac magnetic resonance imaging cohort. After quality control, 1 006 431 exome variants were included, 73.8% had an MAF <0.001, and about 25% had an Ensembl impact level of high or moderate (Figure S11). At the conservative threshold of *P*<3.125×10^−9^, 655 significant genotype-to-spatial trait associations from 154 exome variants were identified, of which 82 associations were from 19 exome variants not identified at GWAS (listed in Table S5). Overall, ExWAS identified variants in 20 of the 42 GWAS loci and 3 additional variants outside the GWAS loci (Figure 6). An example of these additional variants was a rare missense variant in *CSRP3* (rs45550635; MAF 0.5%; missense mutation Trp to Arg [W4R]), which associated with increased strain^circ^ and LV ejection fraction. *CSRP3* is a cardiomyopathy-associated gene implicated primarily with HCM but has not been discovered in previous GWAS. These ExWAS summary statistics were combined with GWAS results to provide a comprehensive interpretation of associated variants.

We analyzed ExWAS variants within the 42 loci identified in the spatial LV trait GWAS. There were 6631 exome variants tested in the 42 loci, of which 5683 were not included in the imputation set where GWAS was performed. We found that 87.1% of these exome-only variants were rare (MAF <0.1%), and 96.3% of those with high impact were also rare (truncating, splice donor, or splice acceptor; Figure S11F). These mostly rare and high-impact exome variants were observed in 14 GWAS loci, of which 10 were also HCM or DCM loci that include stop-gained variants in genes (*CLCNKA*, *TTN*, *CEP85L*, *PRAG1*, *AGAP5*, *SYNPO2L*, *ADPRHL1*, *ALPK3*, *LRRC37A2*, and *SMARCB1*), listed in full in Table S6. The majority of these variants were rare (at the limit of observed at least 5 times in the cohort, MAF <0.01%) and had a larger effect size than GWAS significant lead variants. This catalogs functionally significant variants in reported cardiomyopathy-related genes observed in a population that excluded those with diagnosed cardiomyopathy.

A spatial approach to phenotyping identified loci containing Mendelian genes implicated in cardiac diseases that did not reach significance using global traits (Figure 6). In the *CEP85L*/*PLN* locus, the sentinel variant (rs3734381) has been identified from previous HCM and QRS duration GWAS^28^ but has never been identified for LV wall thickness or mass. Here, we found this variant to be associated with spatially resolved wall thickness with an apical to basal gradient (Figure 6B). In addition, a missense variant in *MYH6* (rs365990) was not found associated with LV mass, ejection fraction, or global strain^rad^; however, it has a spatial strain^rad^ association in the basal-lateral wall (β=−0.05; *P<3*.125×10^−9^; Figure 6C).

### Exome-Wide Gene-Based Tests on Rare Deleterious Variants

We further performed gene-level burden tests to assess the collective effect of rare loss-of-function or deleterious variants, which were too rare to be tested at the variant level. Using masks provided by UKB,^29^ up to 9865 genes were tested for predicted loss-of-function (pLoF) variants and 15 615 genes for deleterious variants using Regenie^30^ (see Methods section for details). Variance component tests that take into account the directionality of effects were also performed on up to 3666 genes for pLoF variants and up to 9732 for deleterious variants. For spatial traits, *P* values of genes tested on all 16 segments by the same test were merged to assess false discovery, and a significance level threshold was set at 5% FDR (*q* value<0.05). Data Set S5 shows the full list of genes that passed this threshold.

For genes harboring rare (MAF <0.01) pLoF variants, *EXD2* and *MYOT* (*P<1*.4 ×10^−5^) significantly associated with LV mass, *EXD2* and *PCDHA12* (*P<7*.8×10^−6^) with global mean wall thickness, and *TTN* with ejection fraction, radial, and circumferential strains. Carriers of *EXD2* rare pLoF variants had decreased LV mass and wall thickness (β=−1.1, −0.9, respectively). *TTN* is a definitive-evidence DCM gene where truncating variants are associated with cardiac phenotypes.^31^ We also identified *ALPK3* as having a spatial wall thickness association through variance component tests (SNP-Set Kernel Association Test [SKAT]: *P=9*.28×10^−8^) that take into account different directions of effect from pLoF variants (Figure S12A). The leave-one-variant-out tests showed the direction of effect for each pLoF variant with respect to changes in wall thickness (Figure S13).

For genes harboring rare predicted deleterious variants, we found an association between *INHBB* with spatial wall thickness and *CSRP3* with spatial and global contractility (Figure S12B). *CSRP3* and *TTN* displayed opposite directions of association with contractility: the deleterious variants of CSRP3 were associated with increased strain^circ^, while *TTN* pLoF variants were associated with decreased strain^circ^. *INHBB* deleterious variants (0.014% of cohort) showed a protective role against increased wall thickness (β=−1.1; *P=1*.84×10^−6^). We also found an association of *AQP1* (aquaporin-1) with spatial wall thickness by the variance component tests (SKAT: *P=3*.90×10^−7^). AQP1 is a water channel protein differentially expressed in cardiovascular tissues where it regulates transmembrane homeostasis and is also associated with cardiovascular outcomes.^32,33^

### Causal Association With Hemodynamics

Both SBP and DBP have an established causal influence on HCM^6,15^ and also mediate elevated LV mass in the general population^34^ although the patterns of remodeling may be distinct in these groups.^5^ MR uses genetic variants that are robustly associated with a complex trait to generate unbiased detection of causal effects. The approach is analogous to the randomized controlled trial but with genetic variants as proxies.^35^ Here, we performed MR between hemodynamic factors and each regional trait of the myocardial wall.

**Figure 7.**
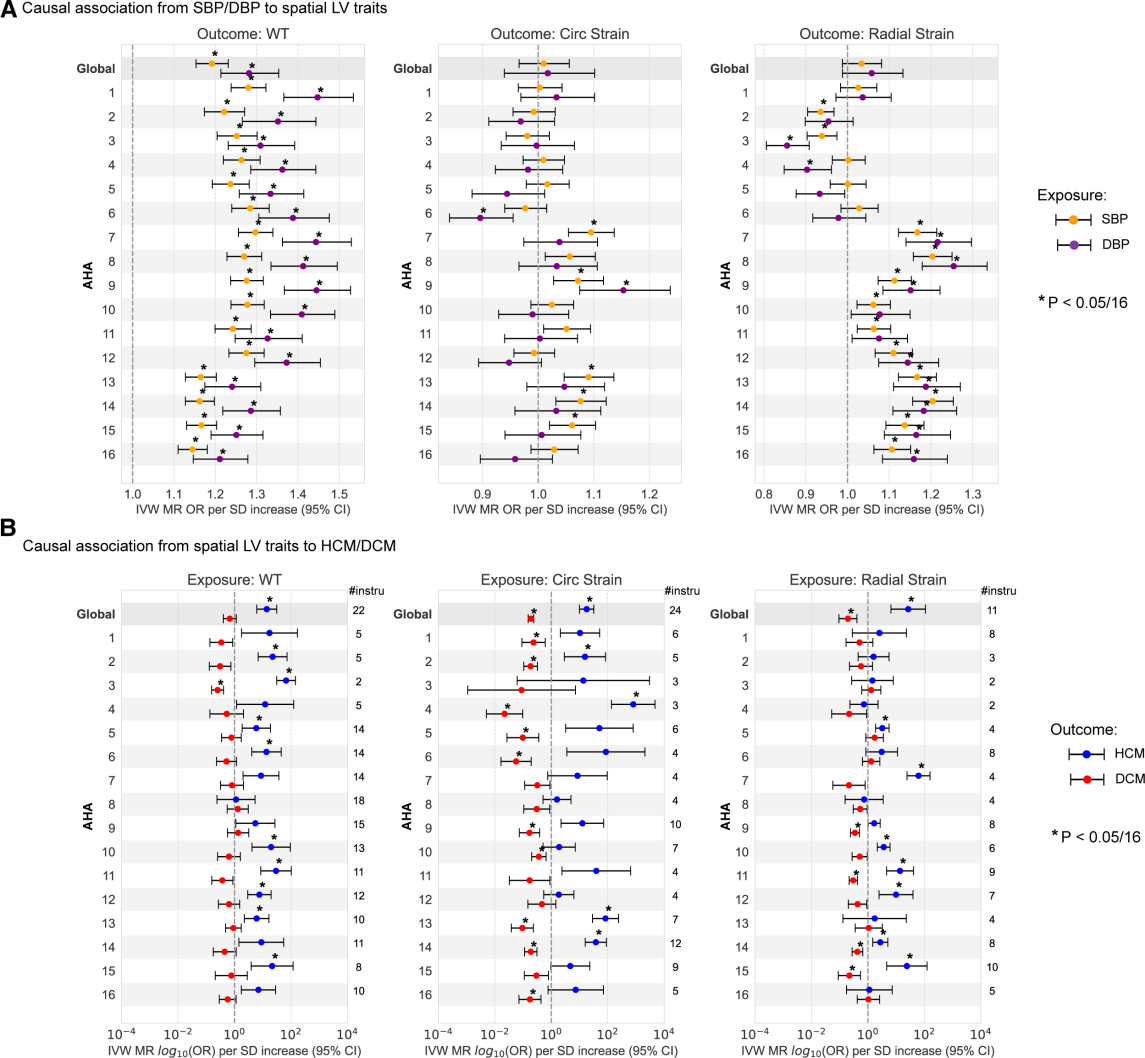
**Mendelian randomization (MR) analysis of blood pressure on spatial traits and spatial traits on the risk of hypertrophic cardiomyopathy (HCM) and dilated cardiomyopathy (DCM).** Odds ratios (ORs) represented are those inferred from the inverse variance–weighted (IVW) 2-sample MR per SD increase. The error bars represent the 95% CI of the OR. OR for circumferential strain reflects those of increased contractility. Asterisk indicates that the MR IVW test *P* value is below multiple hypothesis–adjusted threshold (*P*<0.05/16). **A**, MR results on increased systolic blood pressure (SBP)/diastolic blood pressure (DBP) on risk of increased spatial traits, including the global mean (G) and regional mean on American Heart Association (AHA) segments (1–16) for wall thickness, strain^circ^, and strain^rad^. Spatial traits used a genome-wide association study (GWAS) on rank-based inverse transformed (rIVT) adjusted with sex, age, body mass index (BMI), and body surface area (BSA) at magnetic resonance imaging (MRI), in 40 058 participants of the UK Biobank without cardiomyopathy and with available cardiac magnetic resonance (CMR) imaging. Genetic instruments for SBP and DBP were selected from a published GWAS including up to 801 644 individuals.^13^ **B**, MR results on increased global and spatial left ventricular (LV) wall thickness, and strain in circumferential and radial directions on risk of HCM and DCM. Genetic instruments for spatial traits were selected from the present GWAS, and the number of single nucleotide polymorphisms involved in performing MR to HCM is listed on the right side of each figure. The outcome HCM GWAS included 5927 cases vs 68 359 controls^15^; DCM included 14 255 cases vs 1 199 156 controls.^16^

We used MR Egger to test for causality as this method accounts for pleiotropic effects. Both SBP (Egger: *P*=2.82×10^−12^) and DBP (Egger: *P*=1.65×10^−8^) have a causal relationship with mean wall thickness, where DBP has a higher effect ratio (Figure S14). We further performed the MR analysis of BP on spatial traits, found significant associations at adjusted *P* values (Egger: *P*<0.003125), and performed reverse MR using spatial traits as exposures and BP as the outcome where no significant reverse causality was found. We used the inverse variance–weighted test to estimate the magnitude of causal association and observed a gradient of stronger odds ratios of wall thickness increase per SD change in BP from base to apex for both SBP and DBP (Figure 7A). This shows a causal relationship between BP and LV geometry.

A causal effect of SBP and DBP on myocardial strain was observed regionally although there was no causal effect on global mean strain^circ^ or strain^rad^ (Figure 7B and 2C). Higher SBP and DBP were causally linked to increased strain^rad^ in mid anteroseptal segments (American Heart Association 7, 8; inverse variance–weighted test: *P*=5×10^−5^ and 1×10^−6^ for SBP; *P*=1×10^−4^ and 8×10^−5^ for DBP), while the direction of effect was opposite for inferior basal segments. For spatial strain^circ^, ncreased SBP and DBP are related to decreased strain in basal-lateral segments and increased strain in mid-inferior septal segments although the causal effect is insignificant.

### Causal Association of Spatial Traits and Cardiomyopathy

Prior data from GWAS and MR support a causal role of increased global LV contractility in both obstructive and nonobstructive forms of HCM.^15^ We performed bidirectional 2-sample MR tests between spatial LV traits, and HCM and DCM risks (Data Set S6). The Egger test showed that contractility was causally associated with DCM but not with HCM. We found that the inverse variance–weighted test supported a causal role of global and regional mean wall thickness for HCM, as well as global and regional myocardial strain for HCM and DCM risk (Figure S15B). The Egger test did not provide additional evidence for a causal role of mean wall thickness on HCM or DCM risk, and the high intercept suggested pleiotropic effects (Figure S15).

### Pathways Associated With Remodeling

**Figure 8.**
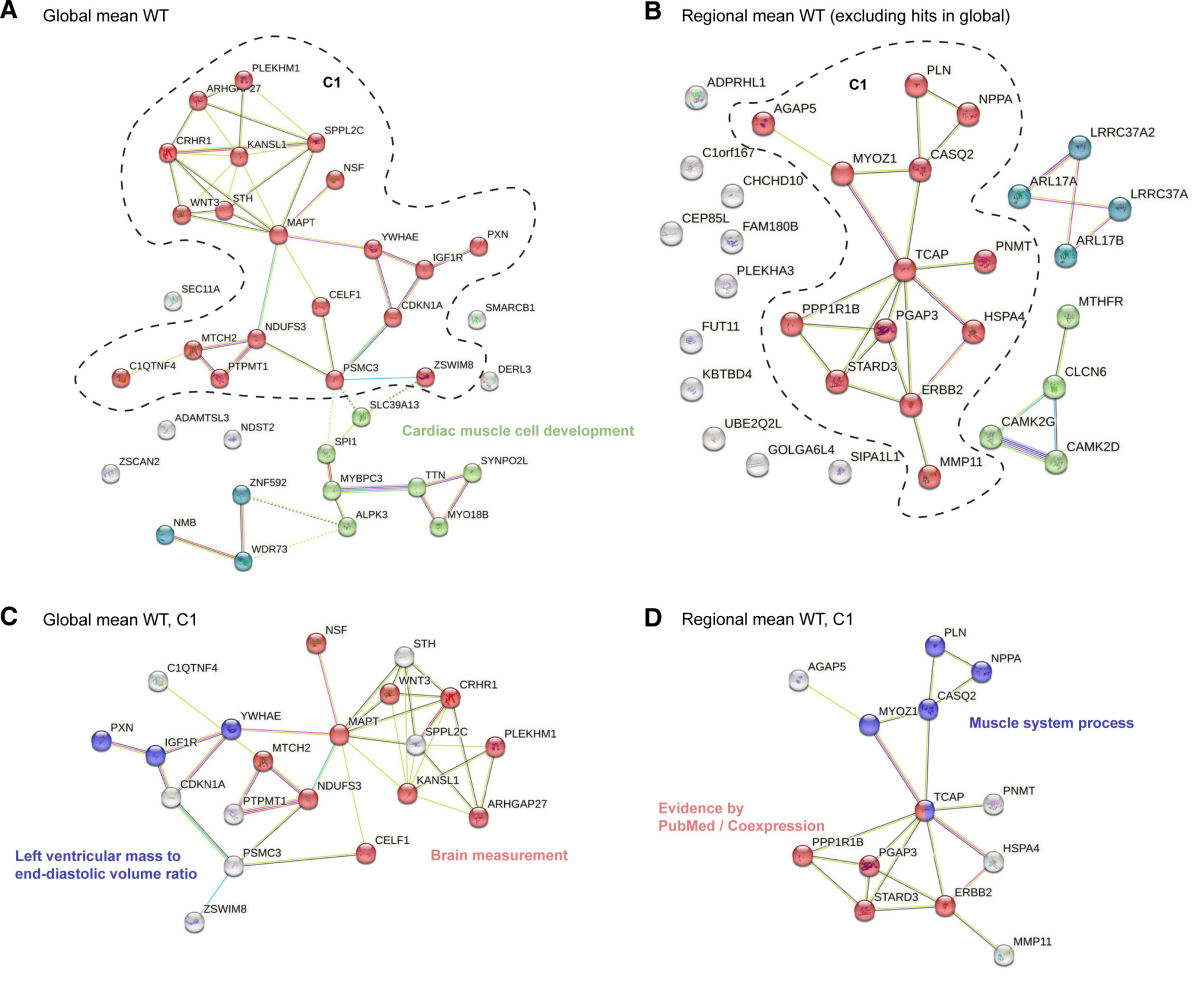
**Cluster groups and gene interactions for left ventricular wall thickness (WT). A**, Genes in the global mean wall thickness genome-wide association study (GWAS) loci were clustered by the STRING-DB. Enrichment analysis on the largest cluster is shown in **C**. Edges show protein-protein relationships where a protein works together to perform a common task. **B**, Genes in the spatial-only loci of regional wall thickness were clustered by the STRING-DB (Search Tool for the Retrieval of Interacting Genes/Proteins). Enrichment analysis on the largest cluster was shown in **D**. Light blue line: known interactions from curated data sets; purple line: experimentally determined known interactions; dark blue line: predicted interactions from gene neighborhood; red line: predicted interactions from gene fusions; dark blue line: predicted interactions from gene co-occurrences; light green line: predicted interaction from text mining; black line: co-expression; and light purple: gene homology.

We next identified potential interactions among protein-coding genes associated with regional traits. We performed unsupervised gene clustering and pathway enrichment analysis on the interaction network using the STRING-DB server (Data Set S7).^36^ Protein-coding genes that were significant in global mean wall thickness were clustered into 3 groups (Figure 8A). The largest cluster contained 10 genes (*WNT3*, *NDUFS3*, *MTCH2*, *MAPT*, *NSF*, *CRHR1*, *KANSL1*, *PLEKHM1*, *ARHGAP27*, and *CELF1*) that were significantly enriched for brain volume and cortical area and 3 genes (*PXN*, *IGF1R*, and *YWHAE*) with enrichment for LV mass to end-diastolic volume ratio. These genes together also constitute a significant enrichment of anthropometric measurements (Figure 8C). The other 2 clusters have enrichment in heart and muscle development (*ALPK3*, *SYNPO2L*, *MYO18B*, *MYBPC3*, and *TTN*) and reported GWAS on image-derived traits (*NMB*, *WDR73*, and *ZNF592*).^7^ The enrichment analysis of mean wall thickness GWAS-identified genes showed processes that are known to affect global development of the LV wall. The rediscovery of genes underlying brain and anthropometric measurement shows that genetic components that underlie muscle and size development have a shared influence across human organs.

Protein-coding genes that were significant in spatial-only loci for wall thickness were clustered into 3 main groups with 11 genes unclustered, suggesting that the pathway information behind the spatial-only hits is less understood (Figure 8B). The largest cluster identified was enriched for muscle system process genes and a cluster centered on *STARD3* (Figure 8D). The *STARD3* gene was reported to contain deleterious or damaging protein-coding variants for heart failure with reduced ejection fraction.^25^

We further included genes in spatial-only loci for strain^circ^ and strain^rad^ in the analysis. These include 84 protein-coding genes identified from the 18 spatial-only loci, and STRING analysis identified 7 clusters (Figure S16). Twenty-two genes constitute the largest connected cluster and are involved in the sarcomere and heart development (*MYH6*, *MYH7*, *PLN*, *ERBB2*, *WNT2*, and *TCAP*), and the ERBB2 pathway (*STAT5A*, *STAT5B*, *HSPA4*, and *NPPA*). The other clusters identified genes involved in the regulation of excitation-contraction coupling in the heart and voltage-gated chloride channels (*CAMK2D*, *IFNGR2*, and *UQCRQ*), genes associated with splicing regulation (*SREK1IP1*, *CWC27*, and *PPWD1*), and genetics associated with cardiac QRS duration and cardiac ventricular conduction (*HEATR5B* and *STRN*).^28^ This analysis provides an extended view of genes and pathways involved in the regulation of cardiac physiology.

## Discussion

The 3D structure of the heart in health and disease shows high endophenotypic diversity, which is predictive of outcomes.^5,37,38^ Here, we aimed to discover the genetic determinants of patterns of structural and functional adaptation in the heart through spatially resolved phenotyping and genetic association analyses. Using deep learning derived anatomic atlases, we found spatially distinct associations in loci related to cardiac chamber development and stress-response pathways. We also discover novel associated loci not detected using global traits, identify potential pathways that regulate phenotypic adaptation, and dissect the causal relationships between environmental stimuli and remodeling.

A spatially resolved GWAS of LV traits enables the discovery of genetic associations that have regional expression. Newly discovered loci include several that are associated with cardiomyopathies in case-control GWAS for HCM (*CEP85L*/*PLN*, *ADPRHL1*, and *SIPA1L1*) and DCM (*HSPA4* and *CAMK2D*), as well as the sarcomeric gene *MYH6*, which is linked to congenital heart disease but has not previously been associated in LV global trait GWAS. Spatial contractile function reveals 2 loci (*STRN* and *PRDM16*) that are also found in cardiomyopathy GWAS. *STARD3* is not related to cardiomyopathy but is associated with heart failure. *PLN* and *MYH7* (eQTL hit in the *MYH6* locus) were both reported as pathogenic genes in HCM.^19^ These findings extend the known repertoire of shared genetic loci between cardiac development and adaptation in adults with GWAS and Mendelian cardiomyopathy–associated genes. Exome-wide analyses discovered a further 3 loci including a missense variant in *CSRP3* (amino acid mutation W4R), which was analyzed in cardiomyopathy Mendelian studies but not yet reported in the GWAS catalog, highlighting the additional power for variant-level discoveries against using impute-only data. Gene-level burden tests looked at the effect of ultrarare pLoF variants. Rare loss-of-function variants in the cardiomyopathy gene *ALPK3*, which regulates cardiomyocyte and myofibroblast differentiation,^39,40^ were associated with increased regional wall thickness. *ALPK3* is one of several reported genes that have a recessive association with cardiomyopathy, supporting a semidominant model of inheritance.^41^ We found that common missense variants in *ALPK3* are associated with decreased wall thickness, whereas the pLoF variants are together associated with hypertrophy, suggesting a potentially protective role for GWAS variants against hypertrophy. Similarly, the sentinel missense variant in *MYH6* was found to be associated with decreased basal wall thickness, whereas predicted damaging variants are associated with increased wall thickness in cardiomyopathy.^42^

BP has a key causal influence on global LV hypertrophy with early involvement of the basal septum.^9,43^ Nonsarcomeric HCM is also characterized as a polygenic hypertrophic sensitivity to DBP.^6^ We showed that there was a spatially varying gradient of causal association between BP and wall thickness from base to apex with DBP having a stronger effect. While BP is the causal exposure, the hypertrophic response depends on wall stress, which is highly heterogeneous in the LV and greatest where the septum is flattest near the outflow tract.^44^ We also found a genetic correlation between physiological variation in hypertrophy and inherited cardiomyopathies with opposite directions of effect in HCM and DCM. Causal analyses show how common genetic variation modifies Mendelian disease risk and morphofunctional expression, contributing to the diversity of cardiomyopathy phenotypes.

Using unsupervised gene clustering and pathway enrichment analysis, we found 10 genes that share an enrichment for brain volume and cortical area. There is a shared genetic influence between markers of heart and brain health,^45^ and our findings suggest that some genes may contribute to multiorgan development and the emergence of complex traits. Genes discovered only through spatial phenotyping revealed 3 main groups with the largest regulating heart regeneration and trabecular development through promotion of cardiomyocyte dedifferentiation and proliferation.^46,47^ The NRG-1/ErbB signaling pathway is involved in diverse aspects of cardiomyocyte biology, which has also been identified as a therapeutic target for cardiomyopathy and even heart regeneration.^48^ Pathway analysis also identified genes involved in cardiac conduction, suggesting that ion channel genes are critical for regulating the contractility of the adult heart and formation of the conduction system.^49^

### Clinical Implications

This work provides a comprehensive resource for understanding the genetic associations of LV morphology and contractile function, as well as the response to stress. This could inform understanding of new monogenic causes of hypertrophy and polygenic modifiers of disease phenotypes. The pathways discovered in this work could also be investigated as potentially druggable targets for modifying adverse changes in ventricular remodeling.

### Limitations

UKB is a prospective cohort study with deep genetic and phenotypic data that are unique in their size and scope.^50^ However, certain age groups and people living in less socioeconomically deprived areas are underrepresented.^51^ The population is predominantly European (95%), and further research is required on people of diverse ancestries and social groups. Cross-ancestry analyses may help in finding new associations and improving the precision of fine mapping. UKB may show latent population stratification; however, risk factor associations seem broadly generalizable.^52^ We used validated computer vision approaches to assess 48 spatial traits in up to 40 058 healthy White individuals describing the landscape of genetic architecture influencing heart structure. Through exome-wide GWAS, we extended genetic discovery beyond common variants, identifying rare variants not included in the imputed genotyping data. The use of independent disease-specific cohorts for MR enhances generalizability and reduces the risk of population stratification bias in causal analysis. However, the study is restricted to White individuals, which limits the translatability of findings to other ancestral groups. Horizontal pleiotropy may still cause bias in causal estimates using MR. The power of exome-based burden tests is also dependent on the loss-of-function variants classification and aggregation strategy. Rare variant analysis has limited statistical power and will require replication with large independent data sets if they become available.

### Conclusions

Together, the data reported here, combining advanced 3D human imaging with genetic analyses across the allele frequency spectrum, highlight the role that cardiomyopathy-associated genes have on the regulation of spatial adaptations in those without known disease.

## Article Information

### Sources of Funding

This work was supported by the British Heart Foundation, United Kingdom (grants FS/IPBSRF/22/27059, RG/19/6/34387, RE/18/4/34215, CH/F/24/90015, RG/F/22/110078, FS/CRTF/21/24183, RG/F/22/110078, RG/F/24/110138, RE/24/130023, SP/17/11/32885, and BBC/F/21/220106); the Medical Research Council, United Kingdom (grants MC-A658-5TY00 and MC_UP_1605/13); the Engineering and Physical Sciences Research Council, United Kingdom (grant EP/W01842X/1); the Sir Jules Thorn Charitable Trust, United Kingdom (grant 21JTA); and the National Institute of Health Research (NIHR) Imperial College Biomedical Research Centre, United Kingdom, and the NIHR University College London Biomedical Research Centre, United Kingdom. The views expressed in this work are those of the authors and not necessarily those of the funders. For open access, the authors have applied a CC BY public copyright license to any Author Accepted Manuscript.

### Disclosures

Dr O’Regan has consulted for Bayer AG and Bristol Myers Squibb. Dr Ware has consulted for MyoKardia, Inc, Pfizer, Foresite Labs, Health Lumen, and Tenaya Therapeutics, and received research support from Bristol Myers Squibb. Dr McGurk has consulted for Checkpoint Capital LP. Dr Zheng has consulted for Health Lumen. None of these activities is directly related to the work presented here. The other authors report no conflicts.

### Supplemental Material

Supplemental Methods

Tables S1–S7

Figures S1–S16

Data Sets S1–S7

References 53–62

## Supplementary Material


