# Supplementary material for: New Genetic Loci Implicated in Cardiac Morphology and Function Using Three-Dimensional Population Phenotyping
Source: Circ Genom Precis Med. 2025 Oct 7;18(5):e005116. doi: 10.1161/CIRCGEN.124.005116 (PMC7618224; doi:10.1161/CIRCGEN.124.005116)
Supplement: Supplementary file 1 [file hcg-18-e005116-s001.pdf]

Supplemental Material

Supplemental methods

Dataset

The UK Biobank[51] (http://www.ukbiobank.ac.uk) is a large study of about half a million individuals who were recruited across the UK from 2006 to 2010 and for whom genetic and extensive phenotype information has been recorded. All participants provided written informed consent for participation in the study, which was approved by the National Research Ethics Service (11/NW/0382). Our study was conducted under terms of access approval numbers 28807 and 40616. A range of available data were included in this study, comprising genotyping arrays and whole exome sequencing (WES), cardiac imaging, and non-imaging phenotypes. These included sex (p31), age (p21003), height (p50), weight (12143), systolic blood pressure (SBP, p4080) and diastolic blood pressures (DBP, p4079). Specifically, the age, weight, and blood pressure measurements were selected by the instance ID of CMR visit. At the time of analysis, there were 502,396 genotyped participants and up to 47,825 participants who have had cardiovascular magnetic resonance (CMR) imaging. 3168 out of the 440,005 UKB participants who have ICD10 records have been diagnosed with cardiomyopathy, which includes DCM (ICD10 codes: I420, I426, I427), HCM (I421, I422) and anyone else with I42 annotation.

The dataset for genetics association analysis on the Caucasians were selected with the following minimum requirements. Caucasians were selected by UKB genetic ethnic grouping (p22006), and those with sex chromosome aneuploidy (p22019), have outliers for heterozygosity or missing rate (p22027), without imputation data (p22828), or have inconsistent sex (p22001 not consistent with p31) were excluded, reducing the genotyped participants for analysis to 408,098. Overlap of these individuals and those with CMR imaging was a cohort of 40,186, of which 128 have been diagnosed with cardiomyopathy by ICD10 records (demographics in Supplementary Table 1). GWAS was performed on individuals without a cardiomyopathy diagnosis to reduce bias towards discovering variants enriched in participants with sporadic penetrant disease in the community. Genetic analysis was performed on up to 40,058 participants with each sample size depending on any missing values of specific covariates or traits. For blood pressure adjusted models we excluded records with missing values which was mainly due to 6777 missing systolic or diastolic readings. There were also up to 139 missing values for image-derived traits.

PheWAS analysis

Phenome-wide association studies (PheWAS) were undertaken using the PheWAS R package with ICD coded clinical outcomes and phenotypes converted to 1,840 categorical PheCodes. We used the standard protocol in the PheWAS package (https://github.com/PheWAS/PheWAS/), which performs logistic regression for logical phenotypes and linear regression for continuous variables. *P* values were deemed significant with Bonferroni adjustment for the number of PheCodes.

CMR imaging protocol and data analysis

A standardised CMR protocol was followed to acquire two-dimensional, retrospectively-gated cine imaging on a 1.5T magnet (Siemens Healthineers, Erlangen, Germany),[54] and the images all underwent standard quality control prior to use in analysis.[9] As previously described,[10,55] automated segmentation of the short-axis and long-axis cine images was performed using fully convolutional networks.[9] Briefly, left ventricular end-diastolic, end-systolic and stroke volume (LVEDV, LVESV, LVSV) (units in mL) and ejection fraction, (LVEF, %), were determined. Myocardial volumes were used to compute left ventricular myocardial mass (LVM, g) assuming a density of $1.05 g.ml^{-1}$. Myocardial wall thickness (WT, mm) measurements were derived from automated segmentation. Motion tracking was performed on the cine images using nonrigid image registration between successive frames.[11] To reduce accumulation of registration errors, motion tracking was performed in both forward and backward directions from the end-diastolic frame and an average displacement field calculated.[34] Circumferential and radial strains were calculated using short axis cines as $E_{dir} = \frac{\Delta L_{dir}}{L_{dir}}$, where dir represents circumferential or radial direction, L_{dir} the absolute length of a line segment along this direction and ΔL_{dir} its change in length over time. The heart was divided into 16 standardised anatomical segments following the AHA protocol, excluding the true apex.[56] Regional longitudinal strain was not calculated due to incomplete spatial coverage of the left ventricle on the long axis cines. Outlier wall thickness values (>20mm) were manually evaluated by checking the underlying CMR image, and erroneous values were removed.

Genetic association analysis

Genome-wide association studies, exome-wide association studies, and exome-wide rare variants analyses were all performed using Regenie(v3.1.1),[30,57] which fits a whole-genome regression model for quantitative and binary phenotypes on a set of high-quality common SNPs (HQ SNPs set), to account for sample relatedness and population structure. All quantitative traits were inverse-rank normalised.

HQ SNPs set

We selected up to 40,058 healthy individuals for whom CMR data were available and for whom genotyping data passed standard quality checks (UK Biobank Resource 531), namely, excluding those with sex mismatch, close relatives (22021), non-Caucasian (22006), sex chromosome aneuploidy (22019) and outliers for heterozygosity or missing rate (22027) and who have withdrawn participation. We further applied quality-control filters on the genotype data following Regenie methods.[30] Specifically, the filters were applied using PLINK2[58,59] (version v2.00aLM, https://www.cog-genomics.org/plink2) and included: a minor allele frequency of $> 1\%$, a Hardy-Weinberg equilibrium (HWE) test not exceeding 1×10^{-15}, a genotyping rate above 99%, not present in high inter-chromosomal LD, in the major histocompatibility (MHC) region, or in regions of low complexity, not involved in inter-chromosomal LD (LD pruning of 1,000 variant windows, 100 variant sliding windows and $r^2 < 0.9$) and in chromosomes 1-22. This resulted in up to 519,697 high-quality genotyped SNPs.

Genome-wide association study (GWAS)

GWAS was performed on 8 million imputed genotypes that passed quality controls, including minor allele frequency (MAF) $> 0.5\%$, imputation score > 0.4, minor allele count > 10, HWE P value $> 1 \times 10^{-15}$, and genotyping missing in $< 1\%$ individuals (7,955,591 SNPs). Quantitative imaging traits were converted to z-scores using rank-inverse-based normal transformation. Body surface area (BSA) was calculated with the Mosteller formula ($\sqrt{\text{Height} \times \text{Weight at MRI}/60}$). Mean arterial pressure (MAP) was calculated as $(2 \times \text{SBP} + \text{DBP})/3$.

In the genome-wide association tests of individual variants, we included covariates: age (age when CMR image was obtained), age squared, sex, age x sex, the top 10 principal components provided by the UK Biobank to appropriately correct for population stratification, BMI and BSA. We performed GWAS with and without adjustment by SBP and DBP. All GWAS were performed using the Regenie (v3.1.1) software. For each trait, Step 1 was run on the HQ SNPs set to obtain the leave-one-chromosome-out (LOVO) predictors. These were then included as covariates in step 2 GWAS on the quality-controlled imputed data. All association analyses used a $\chi^2_{df=1}$ statistic to test a variant for association with a trait (that is $H_0 : \beta_{SNP} = 0$). All programs were called within the UK Biobank research analysis platform (RAP). The results of the regional GWAS analysis on wall thickness, circumferential and radial strain, before and after adjustment by systolic and diastolic blood pressure at MRI are presented in Supplementary Figures 7 to 9.

GREML analysis

SNP-based heritability were calculated with genome-based restricted maximum likelihood (GREML) implemented in the GCTA software (version 1.93.2beta from biocontainers). GREML were produced using GWAS summary statistics where all traits have variance of 1 after the rank-based inverse variance transform (rIVT). The genetic relationship matrix (GRM) was produced from HQ SNPs set using the GCTA software with grm-cutoff set as 0.039. Fixed effects included the sex, age, BMI, BSA, SBP and DBP measured at MRI. As we performed GWAS using rank-based inverse variance transform (rIVT) of theleft ventricular traits, the total variance is 1 for all traits. From GREML outputs, Vp is the phenotypic variance after fixed effects were accounted for. Thus we defined the percentage of variance accountable by fixed effects as 1-Vp.

Genetic correlation

We performed pairwise genetic correlation between the 48 spatial LV traits and SBP, DBP, HCM, DCM using LD score correlation (LDSC, v.1.0.1). GWAS summary statistics on the 48 spatial LV traits came from this paper, and published independent GWAS results were used for SBP, DBP[13], HCM[15], and DCM[16]. For each GWAS, summary statistics was prepared using the 'munge_sumstats.py' command, filtering for the 1000G European SNPs with corresponding alleles using the –merge-alleles 1000G_eur.snplist flag. We then assessed genetic correlations for each pairs using 'ldsc.py –rg', and used the 1000G Phase3 ldscores as reference panel LD Scores. The reference files were downloaded from https://data.broadinstitute.org/alkesgroup/LDSCORE/. We did not constrain the single-trait and cross-trait LD score regression intercepts. Full results in Supplementary Data File 1.

Genome-wide significant loci

To correct for multiple comparisons introduced by regional imaging phenotypes, we applied additional multiple hypothesis correction to the conventional GWAS threshold, further adjusting it with the number of segments tested. The genome-wide

significant threshold for regional traits (which divided the left ventricle into 16 segments) were selected at $P < 5 \times 10^{-8}/16$. We defined genome-wide significant loci by iteratively spanning the +/- 500 kb region round the most significant variant and merging overlapping regions until no genome-wide significant variants were detected within +/- 1Mb, standard specified in[60] and produced with FUMA.[18] By this definition, 42 loci were found altogether for the three imaging traits, including 21 for WT, 16 for strain^{circ} and 18 for the strain^{rad}, as shown in Figure 4a, and listed in Supplementary Data File 4. GWASlab[61] (v3.4.24) was used to make the Manhattan plots. Software for computing q-values from a collection of *P* values is available at: https://github.com/StoreyLab/qvalue.

Gene prioritisation

Variant annotation was performed using FUMA.[18] For the 42 spatial GWAS loci, distribution of gene types encoded by Ensembl (v110) by positional variants, those mapped with eQTL variants in "Heart Left Ventricle" or one of the three relevant tissues (Artery Aorta, Artery Tibial, Artery Coronary, and Heart Atrial Appendage) (GTEx v.8[26]), and those mapped by Chromatin Interaction (CI) in "Left Ventricle" or"Aort" tissues (pre-processed significant loops computed by Fit-Hi-C, filtered at FDR 0.05, GEO accession code GSE87112[62]). For eQTL mapping, only significant cis-eQTL pairs (those within 1MB of the gene) were mapped, where significance was defined by the GTEx pre-calculated FDR (gene q-value ≤ 0.05). Distribution of gene types were shown in Supplementary Figure 6. Nominal genes for each locus were limited to protein coding genes (shown in Figure 4), and prioritised by positional overlap, those regulated by eQTL variants in left ventricle, those mapped by CI in the left ventricle and the known Mendelian genes in cardiac conditions from the CardiacG2P database.[19]

Exome-wide association study

Exome-wide association studies were performed on the final release of whole exome sequencing (WES) data of UKB for the spatial and global left ventricular traits using Regenie (v3.1.1) two-step whole genome regression. The regression model trained on HQ SNPs and the WES data was used to run individual variant associations (minor allele count > 5). After overlap of availability for CMR phenotypes and previous quality checks, the exome-wide associations was run on up to 32,067 individuals (32,067 in spatial WT, 32,053 in spatial strain, 32,065 in LVM, LVEF) and 1,334,918 variants. We further compared the associations results before and after filtering the variants that failed the '90 percent have depth above 10' test (from the '500k_OQFE.90pct10dp_qc_variants.txt' in the UK Biobank helper files), and proceeded only with significant association that passed the test. Finally, the exome variants tested is 1,006,431. Most severe consquences of these variants are annotated using Ensembl Variant Effect Predictor (VEP) functionality with HAIL (HAIL-0.2.78-VEP-1.0.3).

eQTL and transcriptome-wide association study analysis

We performed transcriptome-wide association using the MetaXcan v0.6.12 tools[27] and the GTEx v.8[26] eQTL MASHR-M models (http://predictdb.org/). Each regional GWAS results were harmonized, lifted over to hg38 and linked to the 1000 Genomes Project reference panel using GWAS tools (https://github.com/hakyimlab/summary-gwas-imputation/wiki/GWAS-Harmonization-And-Imputation). Imputed and harmonized GWAS summary statistics were used to perform TWAS for the heart left ventricle in GTEx v.8 with the S-PrediXcan function. Resulting *P* values were corrected using the Bonferroni correction to identify significant gene associations.

Exome-wide gene-based collapsing analysis

The collapsing analyses on gene-based burden testing was performed on the UKB 470K WES release using Regenie (v3.1.1).[30] The analyses were conducted on the Research Analysis Platform (https://ukbiobank.dnanexus.com). The same set of regional CMR phenotypes, whole-genome regression model predictors from Regenie step1, and covariates (sex, age at MRI, BSA, SBP/DBP at MRI, and the 10 ethnicity PCs) were used for collapsing analysis. The intersection of the participants in the GWAS analysis, which was performed on the genotyping array, with those having Exome sequencing data is 38,716 (38,583 have regional mean WT observations, 38,563 have the regional mean circumferential and radial strain observations).

Variant annotations (including missense and loss-of-function predictions) were defined by the UKB final release helper file (ukb23158_500k_OQFE.annotations.txt.gz) on the basis of SnpEff annotations from Ensembl gene definitions. We used two masks for variants: (1) predicted loss-of-functions (pLoF), (2) pLoF or missense (5/5), requiring consensus on deleteriousness from 5 scoring algorithms curated in dbNSFP v3.2. Gene based sets were defined by combining the UKB final release helper file (ukb23158_500k_OQFE_c1-22_n0.sets.tsv) and filtered by the 90pct10dp variants list (ukb23158_500k_OQFE.90pct10dp_qc_variants.txt), which for a given variant, requires at least 90% of genotypes , independent of variant allele zygosity, have a read depth of at least 10. In the final masks for pLoF and pLoF+missense, it is required that the minimum minor allele count (MAC) is above 5.

We performed six types of gene-based tests, namely: the additive burden test, the variance component test (SKAT), the test using Cauchy combination method to combine single-variant-*P*-values (ACATV), and the omnibus test combining SKAT

and burden (SKATO) and its similar alternative using Cauchy combination to maximimze power (SKATO-ACAT) and the omnibus test combing SKAT, burden and ACATV (ACATO). The number tested for regional WT in the burden mask (ADD) is 10768 for pLoF, 15924 for pLoF+missense, and in the variance component tests is 3666 for pLoF, 9732 for pLoF+missense; similar numbers were tested for the regional strains (only 1-2 less tests performed for the burden masks). Since multiple variance component tests were performed, we reported genes that were significant across all six types of tests in the results.

Supplemental Tables

Supplementary Table 1. Mean and standard deviation of selected traits. 47,549 individuals with CMR imaging, and 40,186 Caucasian individuals remained after genotyping quality check. 40,058 individuals with no reported cardiomyopathy were selected for genetics analysis. Although individuals with ICD10 cardiomyopathy were excluded, there remains people in the genetics analysis cohort with abnormal high wall thickness or reduced LVEF: the number with maximum wall thickness over 15 mm is 191, 0.48% of the 40,058 total, and the number with reduced LVEF (below 40%) is 288, 0.72% of the total.

	CMR imaging cohort	Genetic study cohort
Quantitative trait	Mean (SD)	
Age at MRI (year)	64.3 (7.8)	64.5 (7.7)
Weight (kg)	75.3 (15.0)	75.4 (15.0)
Height (m)	169.7 (9.2)	169.9 (9.1)
BMI (kg m-2)	26.0 (4.3)	26.0 (4.3)
Body surface area (m2)	18.8 (2.2)	18.8 (2.2)
Systolic blood pressure (mmHg)	139.5 (18.9)	139.8 (18.9)
Diastolic blood pressure (mmHg)	78.8 (10.1)	78.8 (10.1)
Mean arterial pressure (mmHg)	99.0 (11.7)	99.2 (11.7)
LVEDV (mL)	147.2 (33.9)	147.7 (33.9)
LVESV (mL)	60.1 (19.5)	60.4 (19.5)
LVSV (mL)	87.0 (19.3)	87.3 (19.2)
LVEF (%)	59.6 (6.2)	59.5 (6.2)
LVCO (L/min)	5.4 (1.3)	5.4 (1.3)
LVM (g)	85.9 (22.3)	86.2 (22.3)
RVEDV (mL)	155.9 (37.1)	156.5 (37.1)
RVESV (mL)	67.3 (21.2)	67.5 (21.3)
RVSV (mL)	88.6 (20.4)	89.0 (20.4)
RVEF (%)	57.3 (6.3)	57.3 (6.3)
Mean WT (mm)	5.7 (0.8)	5.7 (0.8)
Maximum WT (mm)	9.5 (1.7)	9.4 (1.6)
Mean radial strain (%)	45.2 (8.5)	45.2 (8.5)
Mean circumferential strain (%)	-22.3 (3.5)	-22.3 (3.5)
Binary trait	n (%)	
Male	22912 (48.2)	19509 (48.5)
Has ICD10 record	40146 (84.4)	33967 (84.5)
DCM	55 (0.1)	42 (0.1)
HCM	42 (0.1)	37 (0.1)
CM	156 (0.3)	128 (0.3)

Supplementary Table 2. Genome-based restricted maximum likelihood (GREML) analysis of LV spatial traits. Table shows in the first three columns the GREML common SNPs heritability for each spatial LV trait. The next three columns shows for each spatial trait, the proportion of variance accounted by both the known confounders, including sex, age, body surface area, SBP and DBP, and the common genetic factors (through the genome relationship matrix, GRM).

	Heritability (h2)			Variance explained		
AHA	WT	Circ Strain	Radial Strain	WT	Circ Strain	Radial Strain
1	19%	17%	14%	46%	21%	18%
2	14%	16%	7%	27%	32%	16%
3	16%	16%	15%	32%	18%	27%
4	22%	7%	16%	51%	8%	28%
5	29%	11%	15%	58%	18%	21%
6	27%	14%	18%	58%	16%	20%
7	25%	14%	16%	64%	24%	24%
8	27%	11%	13%	62%	18%	22%
9	27%	24%	17%	67%	28%	26%
10	27%	6%	16%	68%	7%	22%
11	30%	11%	16%	67%	16%	23%
12	28%	8%	18%	66%	14%	25%
13	16%	17%	14%	53%	24%	21%
14	20%	19%	12%	58%	24%	21%
15	15%	21%	18%	53%	23%	24%
16	15%	19%	18%	53%	21%	22%

Supplementary Table 3. Spatial LV GWAS loci and prioritised genes. The 42 spatial LV loci were shown by if the locus overlap with global LV loci under conventional GWS threshold, and shows which ones were reached indicated thresholds in HCM and DCM GWAS. In HCM GWAS and DCM GWAS columns, ** indicated Pval < 5e-8, * indicated FDR < 0.05. In WT, strain^{circ}, and strain^{rad} columns, * indicates Pval < 3.125e-9 in the one of the spatial LV GWAS. Prioritised genes were mapped by positional SNPs except for the ones indicated with brackets, including those mapped by eQTL, and by chromatin interaction (CI). Full gene prioritisation table is provided in Supplementary Data Tables.

Locus ID	CHR	Start pos (GRCh37)	Locus size (kb)	min Pval	HCM GWAS	DCM GWAS	Prioritised Gene	WT	strain^{circ}	strain^{rad}
Spatial only										
1	1	3197080	65	1.79E-09	*	*	PRDM16			*
11	3	73544836	35	7.59E-10	*		PDZRN3			*
33	14	71697556	498	2.49E-15	*		SIPA1L1	*	*	
36	16	88507538	45	4.81E-11	*		ZFPM1			*
6	2	37059462	218	2.10E-12	**	*	STRN;HEATR5B		*	
17	6	118614518	413	1.55E-11	**		CEP85L;PLN	*		*
26	10	114449904	67	1.16E-09	**		VTI1A			*
29	12	115344085	38	1.71E-11	**		RNFT2,HRK(CI), TBX3		*	*
31	13	114073950	5	1.48E-09	**		ADPRHL1	*		
15	5	132349654	118	1.90E-10		*	HSPA4	*		
13	4	114380213	144	5.69E-12		**	CAMK2D	*		
2	1	11827796	78	9.42E-10			CLCN6	*		
7	2	54725824	240	2.07E-11			SPTBN1;EML6			*
14	5	64273448	57	2.06E-09			CWC27			*
19	7	116879607	34	3.27E-10			WNT2		*	
32	14	23861811	12	7.01E-12			MYH6;MYH7			*
38	17	37741879	142	9.39E-10			ERBB2	*		
40	21	34144545	39	1.94E-09			C21orf49;C21orf62			*
Also in glob										
22	8	124545147	7	7.98E-10	*	*	FBXO32		*	
4	1	116272483	60	6.55E-11	*		CASQ2	*		*
12	4	16028096	9	1.13E-10	*		PROM1		*	
20	8	8088230	296	1.91E-09	*		MFHAS1;ERI1 (CI)			*
37	17	1231593	78	1.46E-10	*		YWHAE	*		
8	2	179381323	473	1.63E-11	**	*	PLEKHA3;TTN	*	*	*
34	15	84488529	944	7.47E-19	**	*	NMB;ALPK3	*	*	
3	1	16131112	235	5.14E-12	**	**	HSPB7;CLCNKA		*	*
16	6	36618821	41	2.60E-17	**	**	CDKN1A	*	*	*
23	8	125849614	52	2.90E-10	**	**	MTSS1;SQLE (eQTL)		*	
27	10	121414236	34	3.64E-15	**	**	BAG3		*	
39	17	43463493	1402	1.32E-15	**	**	MAPT;KANSL1;WNT3	*		
41	22	24111044	71	9.78E-14	**	**	SMARCB1;DERL3	*	*	
24	10	75404300	181	5.05E-11	**		MYOZ1;SYNPO2L	*		
28	11	47365014	632	9.73E-10	**		MYBPC3;SPI1	*		
5	1	236841577	13	1.11E-12		**	ACTN2		*	
9	2	218251702	62	8.29E-10			DIRC3	*		
10	3	69796492	113	2.54E-12			MITF			*
18	7	46609344	59	7.95E-10			IGFBP1;IGFBP3(CI)	*		
21	8	11776904	60	9.92E-13			DEFB136;DEFB135		*	*
25	10	112544125	0	5.27E-10			RBM20		*	
30	12	120646830	52	1.41E-09			PXN	*		
35	15	99249029	47	2.72E-15			IGF1R	*		
42	22	26155484	9	8.34E-19			MYO18B	*		*

Supplementary Table 4. Cross check Spatial GWAS genes with Cardiac G2P database. Cardiac G2P mapped genes were listed by disease grouping[19].

Cardiac Disease grouping	Cardiac G2P mapped genes	Found in Spatial LV GWAS
Classic CPVT phenotype	RYR2,CASQ2	CASQ2
Familial dilated cardiomyopathy	BAG3,DES,DSP,FLNC,LMNA, MYH7,PLN,RBM20,SCN5A, TNNC1,TNNT2, TTN	BAG3,MYH7, PLN,RBM20, TTN
Familial hypertrophic cardiomyopathy	ACTC1,MYBPC3,MYH7,MYL2, MYL3,PLN,TNNI3,TNNT2, TPM1	MYBPC3,MYH7, PLN
Rare syndromic disorder with hypertrophic cardiomyopathy - isolated LVH	ALPK3,CACNA1C,DES,FHL1, FLNC,GLA,LAMP2, PRKAG2,PTPN11, PTPN11,RAF1,RIT1,TTR	ALPK3
Syndrome with hypertrophic cardiomyopathy - no isolated LVH	ABCC9,BAG3,CRYAB, FXN,GAA,MYO6,SLC25A4	BAG3

Supplementary Table 5. The Exome variants significantly associated (min *P* < 3.125e-9) with spatial LV traits, but were not included in the impute data on which GWAS were performed. The list shows 19 Exome variants, and for each variant its location in GRCh38, the effect allele (EA), the effect allele frequency (EAF), the most severe Ensembl consequence (VEP), and the gene symbols.

GWAS locus ID	CHR	POS	EA	NEA	EAF	VEP	Gene(s)
6	2	36883902	A	T	0.560971	intron	STRN
6	2	37007376	C	T	0.581296	intron	HEATR5B
8	2	178612605	A	AT	0.212555	intron	AC010680.4, TTN, TTN-AS1
16	6	36678991	CTA	C	0.195241	intron	CDKN1A, DINOL, LAP3P2, PANDAR
16	6	36679011	G	A	0.326233	intron	CDKN1A, DINOL, LAP3P2, PANDAR
-	10	43616994	G	A	7.80E-05	synonymous	ZNF485
24	10	73650119	T	C	0.149312	intron	AC073389.2, SYNPO2L
24	10	73682785	T	C	0.136494	missense	AGAP5, BMS1P4-AGAP5
-	11	19192439	G	A	0.00497613	missense	CSRP3, CSRP3-AS1
39	17	45816403	G	GC	0.222562	intron	CRHR1, LINC02210-CRHR1, MAPT-AS1
39	17	45816406	T	TGCCTG	0.222557	intron	CRHR1, LINC02210-CRHR1, MAPT-AS1
39	17	46171448	A	G	0.223671	synonymous	KANSL1
39	17	46171471	C	T	0.186527	missense	KANSL1
39	17	46171730	C	A	0.1757	synonymous	KANSL1
39	17	46171833	G	T	0.172555	missense	KANSL1
39	17	46548981	C	T	0.198277	missense	ARL17A, LRRC37A2
39	17	46704913	G	C	0.179936	intron	NSF
-	17	64897294	G	A	0.195414	5 prime UTR	AC103810.2, AC103810.5, AC103810.7, LRRC37A3
42	22	25768112	T	C	0.433029	splice region	MYO18B

Supplementary Table 6. List of Exome loss-of-function or splice variants observed in the GWAS loci. Genes and consequence came from running VEP, 'genes' column included all gene symbols that the variant might influence, and 'consequence' column shows the most severe consequence. Beta (max) and MLOG1OP (max) were respectively the largest absolute beta values and the largest -log10 of P values from testing the 16 segments of LV on WT, radial and circumferential strain, the LVEF, LVM and global max and mean WT, global mean radial and circumferential strain.

GenomicLocus	Gene(s)	Variant (GRCh38:NEA:EA)	MAF	Consequence	BETA (max)	MLOG10P (max)
2	C1orf167	chr1:11768081:C:T	0.00017152	stop gained	0.773755	2.03526
2	C1orf167	chr1:11768102:C:T	7.80E-05	stop gained	-0.487175	0.858324
2	C1orf167	chr1:11775536:G:A	7.80E-05	stop gained	0.993422	1.70652
2	C1orf167	chr1:11784298:C:T	0.00062369	stop gained	0.432227	2.33912
2	C1orf167	chr1:11784463:C:T	0.0288459	stop gained	-0.0583737	2.00594
2	C1orf167	chr1:11785177:TGC:T	7.80E-05	frameshift variant	0.639418	0.87988
2	C1orf167	chr1:11789438:C:T	0.00012474	stop gained	0.731767	1.95578
3	CLCNKA	chr1:16032247:C:T	0.00046777	stop gained	-0.401888	1.71203
3	CLCNKA	chr1:16026765:TCCCTTCAGCGGTGAGACCCCCCTCATGCCCCGCC:T	0.00065691	splice donor variant	-0.409451	2.36107
3	CLCNKA	chr1:16028061:C:T	0.00038981	stop gained	0.388967	2.44515
3	ZBTB17	chr1:15946317:C:G	7.80E-05	splice acceptor variant	-1.01736	1.80327
8	TTN	chr2:178579702:G:A	7.80E-05	stop gained	-1.43759	3.1917
8	TTN-AS1	chr2:178615321:A:G	0.00029626	splice acceptor variant	0.414412	2.12027
8	CCDC141	chr2:178837693:G:A	0.0004054	stop gained	-0.318306	1.17162
8	CCDC141	chr2:178978618:TC:T	0.00054573	frameshift variant	-0.371958	1.86696
8	CCDC141	chr2:178834173:T:A	0.00035862	stop lost	-0.341691	1.3302
8	TTN	chr2:178528273:C:T	7.80E-05	splice donor variant	-0.842612	1.46746
8	TTN	chr2:178653473:CT:C	0.0002027	frameshift variant	-0.446397	1.06941
8	TTN	chr2:178662420:T:A	9.36E-05	splice acceptor variant	0.627603	1.38918
8	TTN	chr2:178663903:C:T	7.80E-05	splice acceptor variant	-1.0964	2.76886
8	TTN	chr2:178664443:GACAGTTAAGAATGTACCTTTGACAGGTACA:G	0.00032907	splice donor variant	0.407317	1.36414
8	TTN	chr2:178665777:G:A	0.00014034	stop gained	-0.746017	1.75502
8	TTN	chr2:178677634:TG:T	0.00015592	frameshift variant	-0.732263	2.67767
8	TTN	chr2:178689289:C:A	0.00010915	splice donor variant	-0.770963	1.45558
8	TTN	chr2:178689897:C:T	0.00059257	splice acceptor variant	-0.238994	0.938127
8	TTN	chr2:178745884:C:A	0.00014033	stop gained	0.913398	2.35187
8	TTN	chr2:178746047:G:C	9.36E-05	stop gained	0.730598	1.23884
8	TTN	chr2:178749346:CCCTG:C	0.00012474	frameshift variant	0.808616	1.71903
8	TTN	chr2:178749351:ATG:A	0.00012474	frameshift variant	0.808616	1.71903
8	TTN	chr2:178749358:TGC:T	0.00012474	frameshift variant	0.808616	1.71903
8	TTN	chr2:178751617:AT:A	0.00010915	frameshift variant	0.814172	1.60621
8	TTN	chr2:178756292:T:TC	0.00049897	frameshift variant	0.35086	2.07556
8	TTN	chr2:178758982:A:G	7.80E-05	splice donor variant	-1.1297	2.27518
8	TTN-AS1	chr2:178778004:G:A	7.80E-05	splice donor variant	-1.46599	5.08606
17	CEP85L	chr6:118465397:ACACTTGAT:A	9.36E-05	stop lost	1.04589	2.19619
17	CEP85L	chr6:118652721:CTGAT:C	0.00037445	frameshift variant	-0.406472	1.46053
20	PRAG1	chr8:8378000:G:A	0.00023389	stop gained	0.655165	2.19556
20	PRAG1	chr8:8378066:G:A	0.00010915	stop gained	-0.813455	2.41299
24	NDST2	chr10:73808145:G:A	7.80E-05	stop gained	1.05322	2.18563
24	FUT11	chr10:73772572:G:A	9.36E-05	stop gained	-1.14761	2.39067
24	FUT11	chr10:73773484:G:T	0.00018711	stop gained	-0.882416	2.75779
24	AGAP5	chr10:73674703:G:A	0.00018711	stop gained	0.639122	1.63075
24	AGAP5	chr10:73675604:A:T	9.36E-05	stop gained	-0.773698	1.8186
24	SYNPO2L	chr10:73653643:C:A	0.00014033	stop gained	0.84727	2.02775
24	AGAP5	chr10:73682707:T:TTG	9.36E-05	frameshift variant	-0.745795	1.86109
24	SYNPO2L	chr10:73647531:A:AG	0.00086754	frameshift variant	0.378353	2.39022
28	AGBL2	chr11:47667666:TC:T	0.00014034	frameshift variant	0.792264	2.15626
28	C1QTNF4	chr11:47590761:AGGGCCCAGCAGGCCGCT:A	0.00042143	frameshift variant	-0.434965	1.75612
28	C1QTNF4	chr11:47590762:G:GGGCCCAGCAGGCCGCTGGGCCCAGCA	0.00015608	frameshift variant	-0.727352	1.91261
28	C1QTNF4	chr11:47590762:GGGCCCAGCAGGCCGCTGGGCCCAGCA:G	0.00012487	frameshift variant	0.698676	1.36325
28	FAM180B	chr11:47588126:C:CT	0.00042099	frameshift variant	-0.356831	1.35924
28	FAM180B	chr11:47588313:G:A	0.0002027	stop gained	0.577382	1.5162
28	KBTBD4	chr11:47573330:T:TC	7.80E-05	frameshift variant	0.739412	1.14225
28	KBTBD4	chr11:47578911:C:G	9.36E-05	splice donor variant	-1.0408	2.11123
28	NDUFS3	chr11:47579334:CGTGA:C	0.00023389	splice donor variant	-0.476536	1.35272
28	NDUFS3	chr11:47580713:TA:T	7.80E-05	frameshift variant	-0.810483	1.83795
28	PTPMT1	chr11:47571628:G:C	0.00394487	stop lost	-0.0934814	0.929256
28	SLC39A13	chr11:47415038:G:A	9.36E-05	splice acceptor variant	0.694338	1.10392
28	RAPSN	chr11:47438712:AC:A	9.39E-05	frameshift variant	1.25403	2.85113
30	PXN	chr12:120216728:AC:A	0.00032744	frameshift variant	0.339037	0.95313
31	ADPRHL1	chr13:113424310:G:A	7.80E-05	stop gained	1.05364	1.80184
34	ALPK3	chr15:84856391:G:A	9.36E-05	splice acceptor variant	1.2655	3.51923
34	ADAMTSL3	chr15:83838216:G:A	0.00014034	splice donor variant	-0.55498	1.31167
34	ALPK3	chr15:84817508:CG:C	0.00021758	frameshift variant	-0.709746	2.06313
34	ALPK3	chr15:84863641:G:GT	0.00010915	splice donor variant	1.04933	2.77413
34	ALPK3	chr15:84868257:C:CT	0.00014033	frameshift variant	-0.757259	1.78261
34	WDR73	chr15:84643474:CG:C	0.00098235	frameshift variant	0.237993	1.29681
34	WDR73	chr15:84643679:C:A	9.36E-05	stop gained	-0.75819	1.67919
34	WDR73	chr15:84654233:C:G	0.00012474	splice donor variant	0.765469	1.64125
34	SLC28A1	chr15:84887782:C:T	0.00028066	stop gained	0.414566	1.3446
38	PGAP3	chr17:39672852:T:C	0.00017152	stop lost	0.591775	1.42737
38	PNMT	chr17:39670186:GC:G	0.00020271	frameshift variant	0.585403	1.61095
38	STARD3	chr17:39660527:G:A	9.36E-05	splice donor variant	0.890304	1.56795
39	LRRC37A2	chr17:46548749:A:T	0.00012474	stop gained	0.844679	1.88409
39	LRRC37A2	chr17:46548914:TC:T	7.80E-05	frameshift variant	-0.907647	1.49185
39	KANSL1	chr17:46094507:C:T	0.0003119	splice donor variant	-0.524346	1.82778
39	MAPT	chr17:45983226:G:GC	0.00014033	frameshift variant	-0.599284	1.32848
39	MAPT	chr17:45985745:G:A	0.00010915	splice donor variant	-0.758089	1.45605
40	C21orf62	chr21:32794101:G:T	0.00023389	stop gained	-0.421833	1.06357
41	DERL3	chr22:23838364:GC:G	0.00163725	frameshift variant	-0.241464	1.99186
41	MMP11	chr22:23772980:T:C	7.80E-05	splice donor variant	0.906691	1.50627

Supplemental Figures

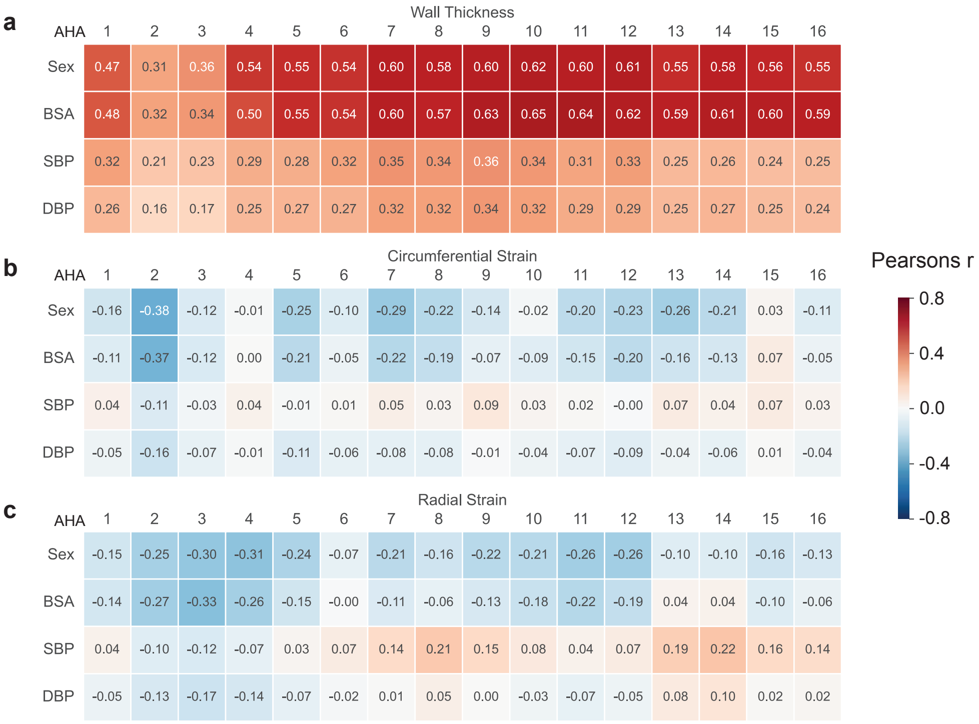

Supplementary Figure 1. LV spatial traits correlations to known confounding factors. Association of the spatial wall thickness (a), strain^{circ} (b) and strain^{rad} (c) with Sex, body surface area (BSA), systolic (SBP) and diastolic (DBP) blood pressure. Pearson correlation values are shown for each indicated regional trait and the indicated known predictor on the left. Values were calculated with DataFrame.corr function in pandas v2.1.3.

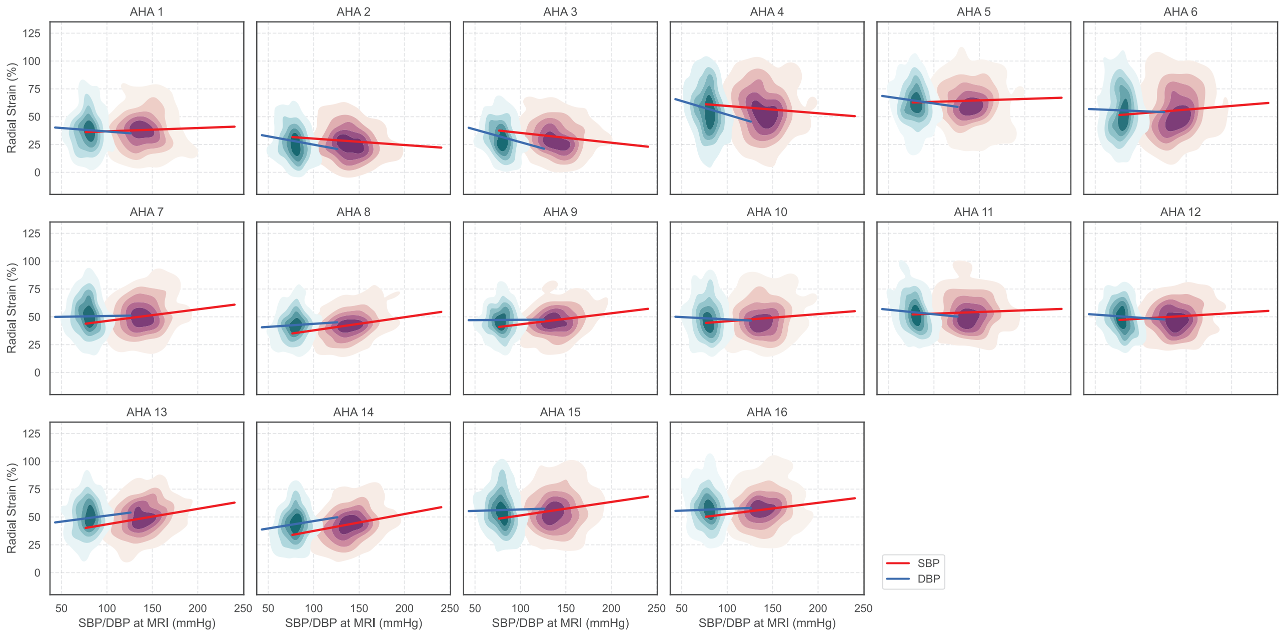

Supplementary Figure 2. Association of LV spatial strain^{rad} with systolic (SBP) and diastolic (DBP) blood pressure. Each graph displays a kernel density plot of mean strain^{rad} on an AHA segment against automatic SBP (red cloud) and DBP (blue cloud) readings taken at MRI. $n = 40,058$ participants were included in the analysis. Plot produced with kdeplot and regplot functions in seaborn v0.12.2.

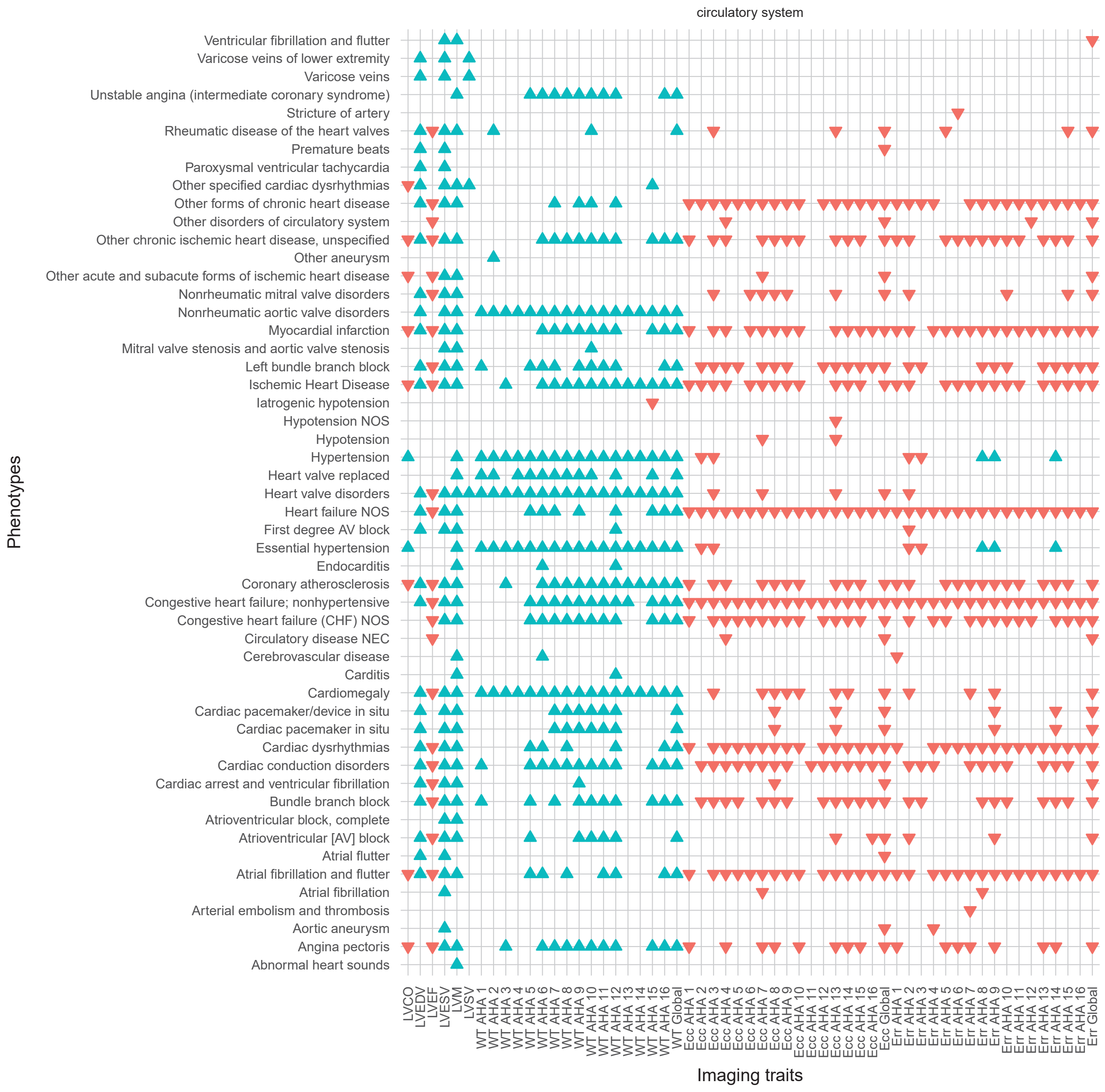

Supplementary Figure 3. Phenome-wide association study of spatial left ventricular traits on cardiovascular phenotypes. After adjustment for known confounders (sex, age, body surface area, SBP, DBP), each spatial trait was assessed for association with cardiovascular phenotypes. Phenotypes as phecodes are described on the y-axis with the phecode category separating the groups and the imaging traits are on the x-axis. Each point denotes a significant PheWAS association with a Bonferroni correction for 1,840 analyzed phecodes. The shape and colour denote the direction of effect. See the Supplementary Data for the full PheWAS results.

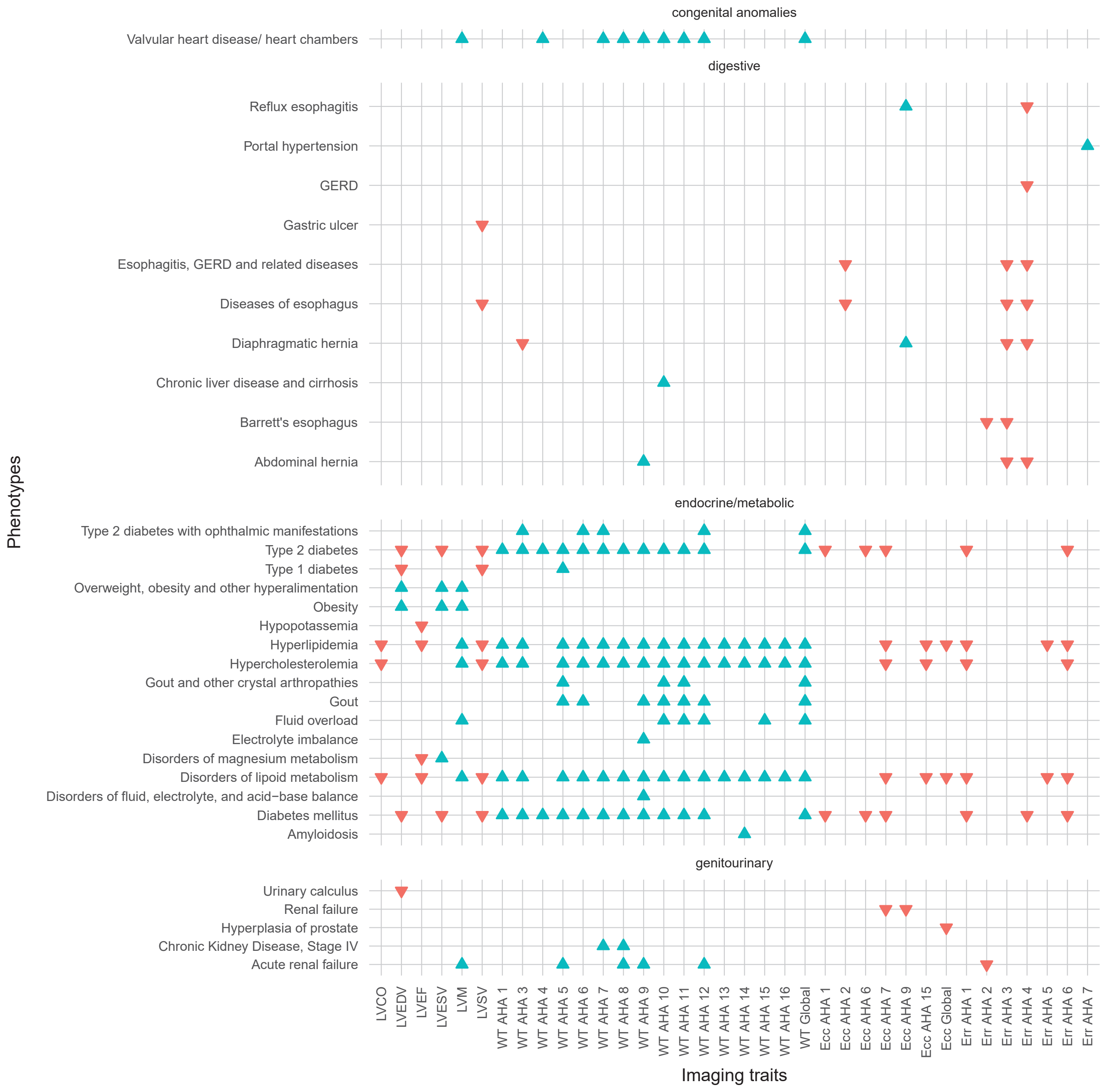

Supplementary Figure 4. Phenome-wide association study of spatial left ventricular traits on non-cardiovascular phenotypes. After adjustment of known confounders (sex, age, body surface area, SBP, DBP), each spatial trait was assessed for association with non-cardiovascular phenotypes. Phenotypes as phecodes are described on the y-axis with the phecode category separating the groups and the imaging traits are on the x-axis. Each point denotes a significant PheWAS association with a Bonferroni correction for 1,840 analyzed phecodes. The shape and colour denote the direction of effect. See the Supplementary Data for the full PheWAS results.

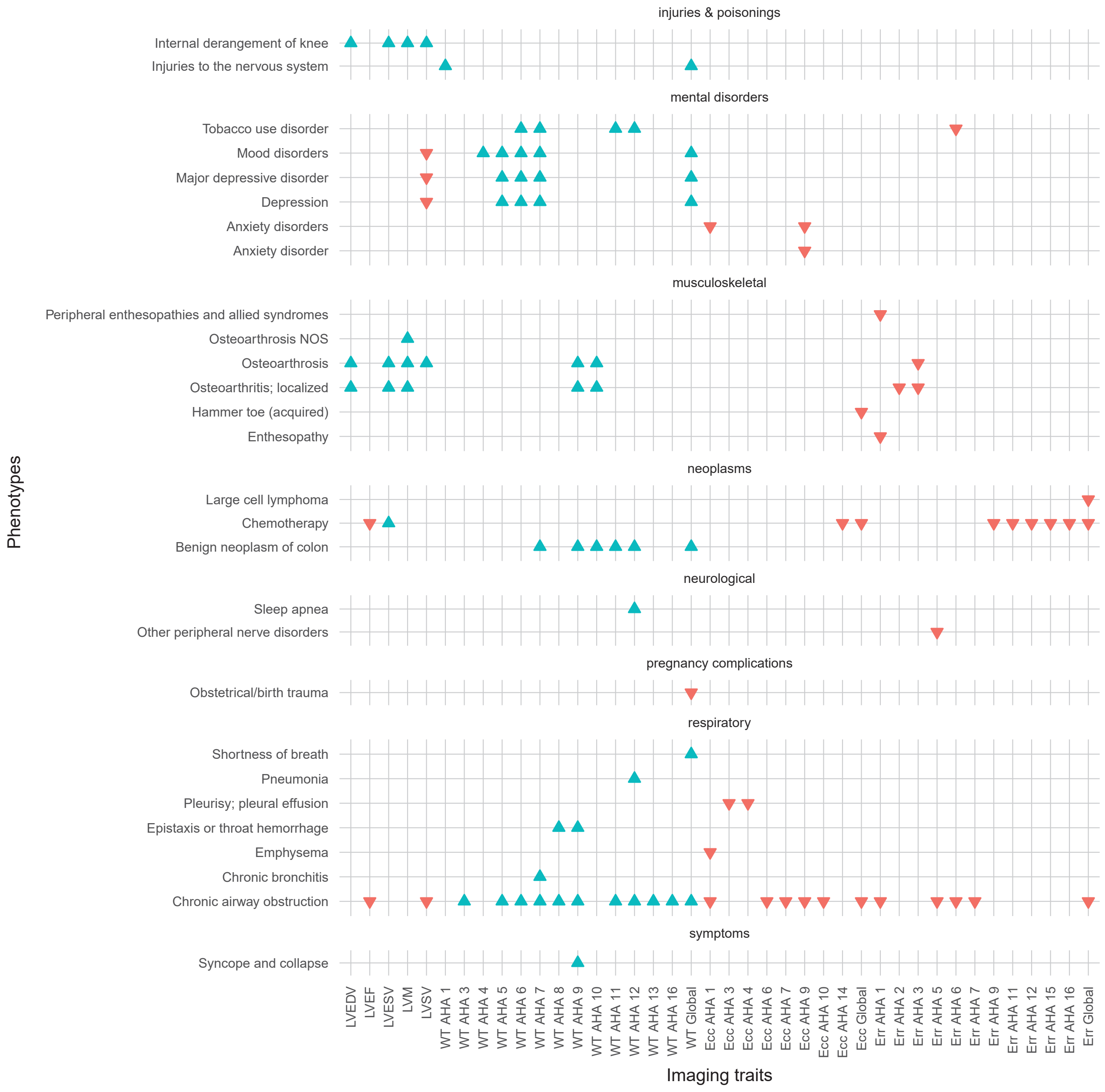

Supplementary Figure 5. Phenome-wide association study of spatial left ventricular traits on additional non-cardiovascular phenotypes. After adjustment of known confounders (sex, age, body surface area, SBP, DBP), each spatial trait was assessed for association with non-cardiovascular phenotypes. Phenotypes as phecodes are described on the y-axis with the phecode category separating the groups and the imaging traits are on the x-axis. Each point denotes a significant PheWAS association with a Bonferroni correction for 1,840 analyzed phecodes. The shape and colour denote the direction of effect. See the Supplementary Data for the full PheWAS results.

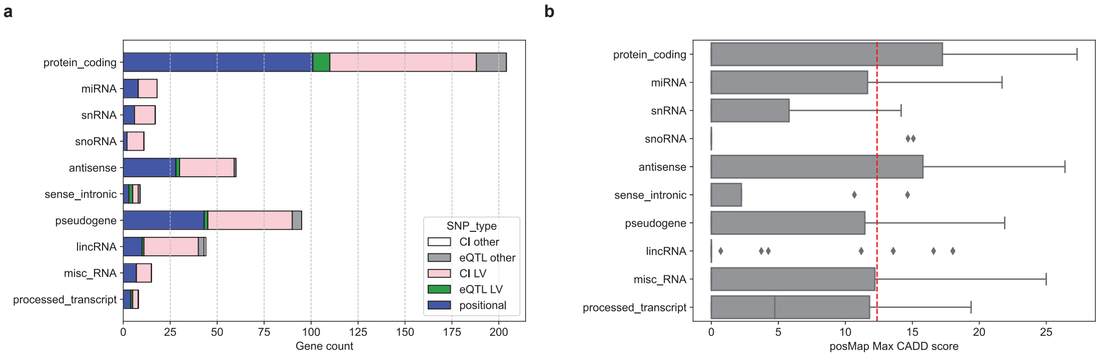

Supplementary Figure 6. Spatial GWAS gene types by FUMA. (a) Distribution of gene products identified in the 42 loci by gene types, prioritised by: (1) if significant SNPs were found in the gene product (blue), (2) if the gene product contains no such positional SNPs, gene products that were mapped by cis-eQTL variants in the heart left ventricle (GTEx8) (green), (3) if none above were found, if they were mapped by chromatin interaction (CI) overlap in left ventricle (pink), and lastly, if they are mapped by eQTL and CI in other 4 tissues (Heart Aorta Appendage, Arteria, Artery A, and Atery Tibial). Total number of gene products was 482, and 455 (94%) were mapped by positional genes, or eQTL or CI in heart left ventricle. (b) Distribution of maximum CADD scores for gene products where positional SNPs were found. The gene products and CADD scores were annotated using FUMA. Legends. miRNA: small RNA (22bp) that silences the expression of target mRNA; snRNA: Small RNA in the cell nucleus involved in the processing of pre messenger RNAs; snoRNA: Small RNA in the cell nucleolus involved in the post-transcriptional modification of other RNAs, antisense: transcripts on the opposite strand, sense intronic: long non-coding transcript in introns of a coding gene, pseudogene: a gene that has homology to known protein-coding genes but contain a frameshift and/or stop codon(s) which disrupts the open reading frame (ORF). Thought to have arisen through duplication followed by loss of function, lincRNA: long intergenic ncRNA, misc RNA: miscellaneous RNA that cannot be classified, processed transcript: transcript that doesn’t contain an ORF.

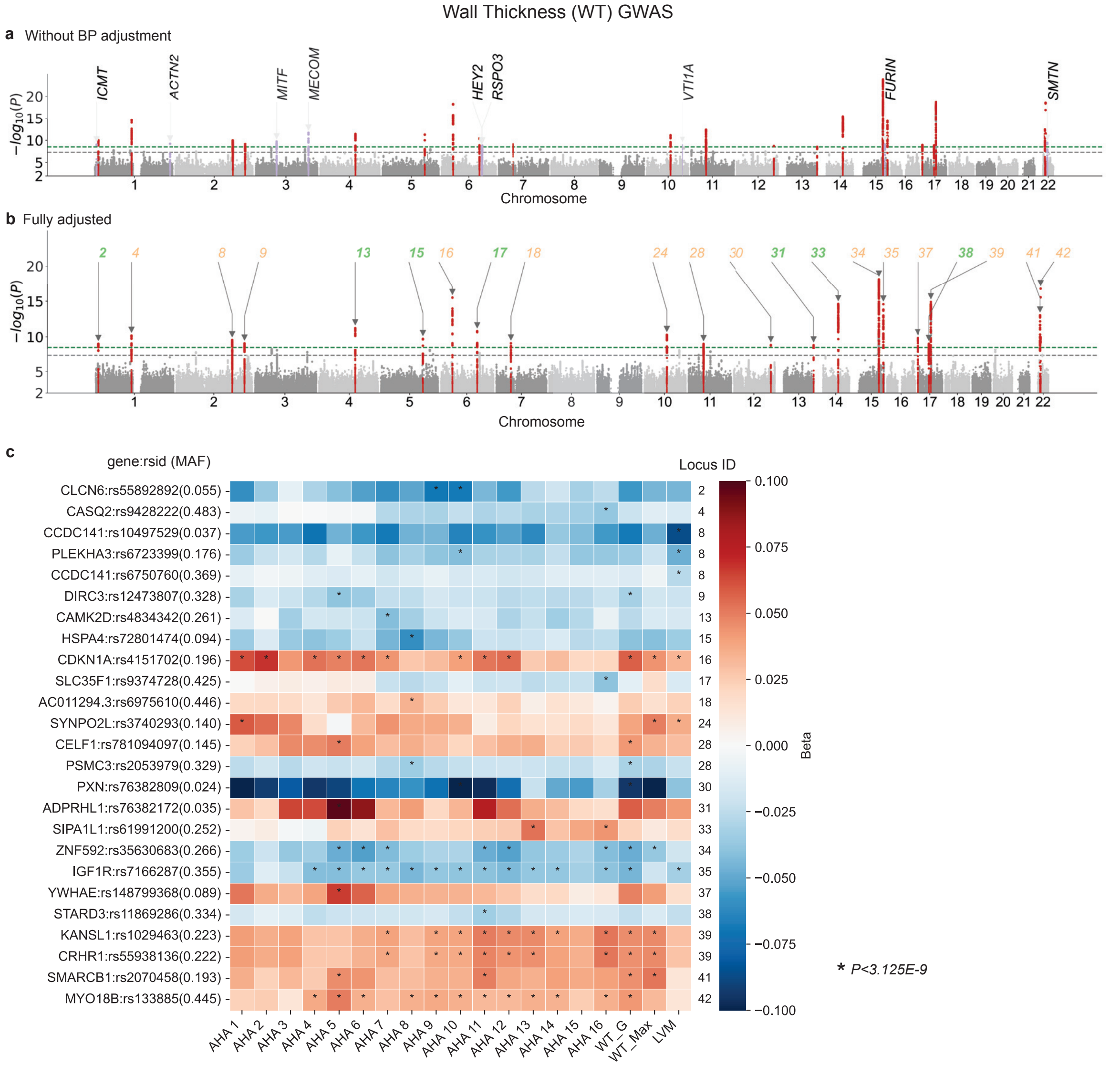

Supplementary Figure 7. Manhattan plots for the wall thickness spatially resolved by the AHA-16 model. (a) The wall thicknesses is adjusted by Sex, age at MRI, age squared, BMI, BSA and the top 10 PCs. Minimum P value found by GWAS on 16 segments were shown in the manhattan plot. Highlights in red show the loci identified in fully adjusted GWAS (panel b), highlights in purple show additional loci that were no longer significant after adjustment with BP. These loci (purple) were annotated by the nearest gene. (b) The wall thicknesses are additionally adjusted by SBP and DBP measured at MRI. (c) Heatmap showing the beta values of lead SNPs in each locus (by fully adjusted GWAS) with the AHA segments on LV.

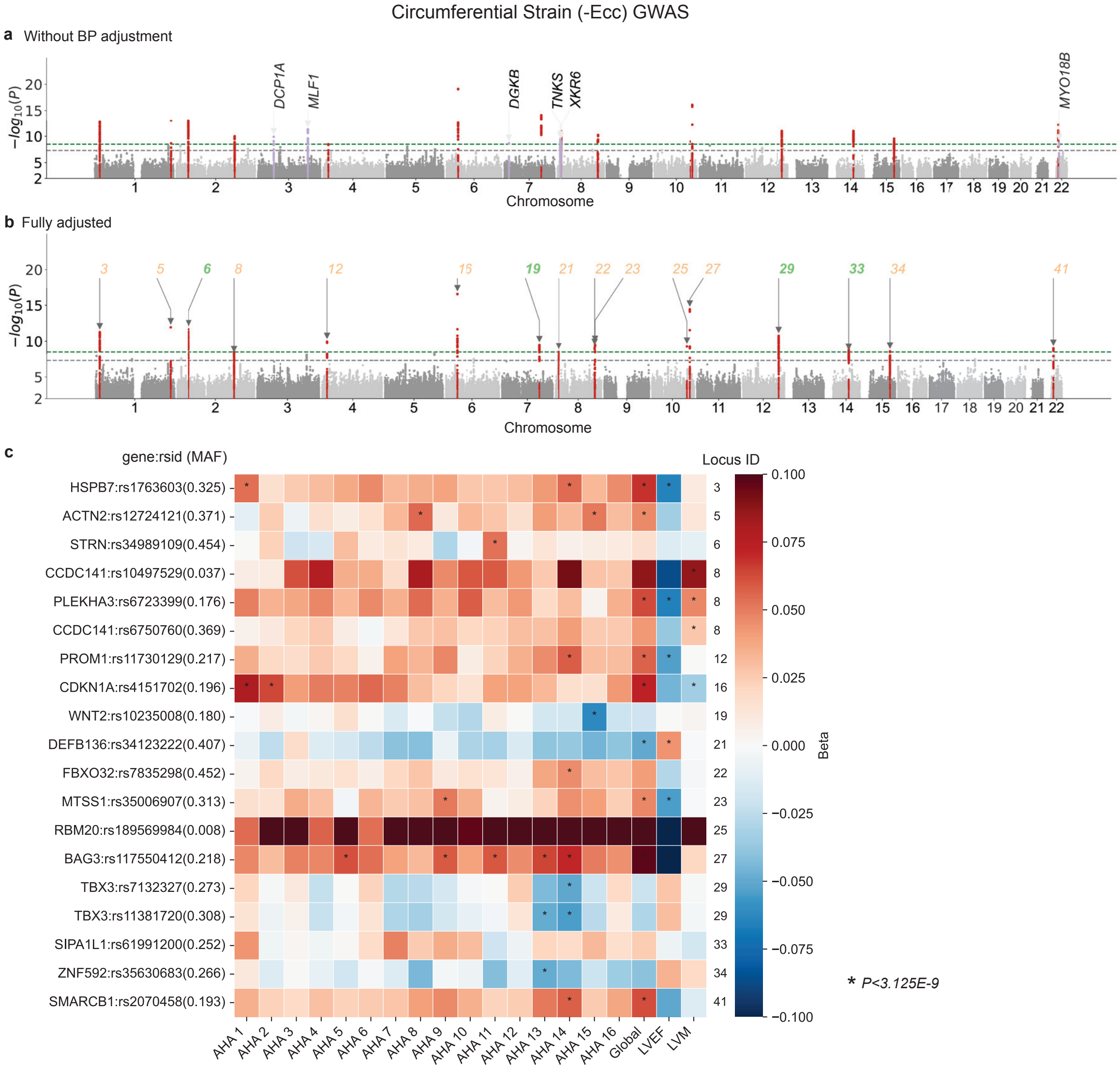

Supplementary Figure 8. Manhattan plots for the circumferential strain spatially resolved by the AHA-16 model. (a) The circumferential strain is adjusted by Sex, age at MRI, age squared, BMI, BSA and the top 10 PCs. Minimum P value found by GWAS on 16 segments were shown in the manhattan plot. Highlights in red show the loci identified in fully adjusted GWAS (panel b), highlights in purple show additional loci that were no longer significant after adjustment with BP. These loci (purple) were annotated by the nearest gene. (b) The circumferential strain are additionally adjusted by SBP and DBP measured at MRI. (c) Heatmap showing the beta values of lead SNPs in each locus (by fully adjusted GWAS) with the AHA segments on LV.

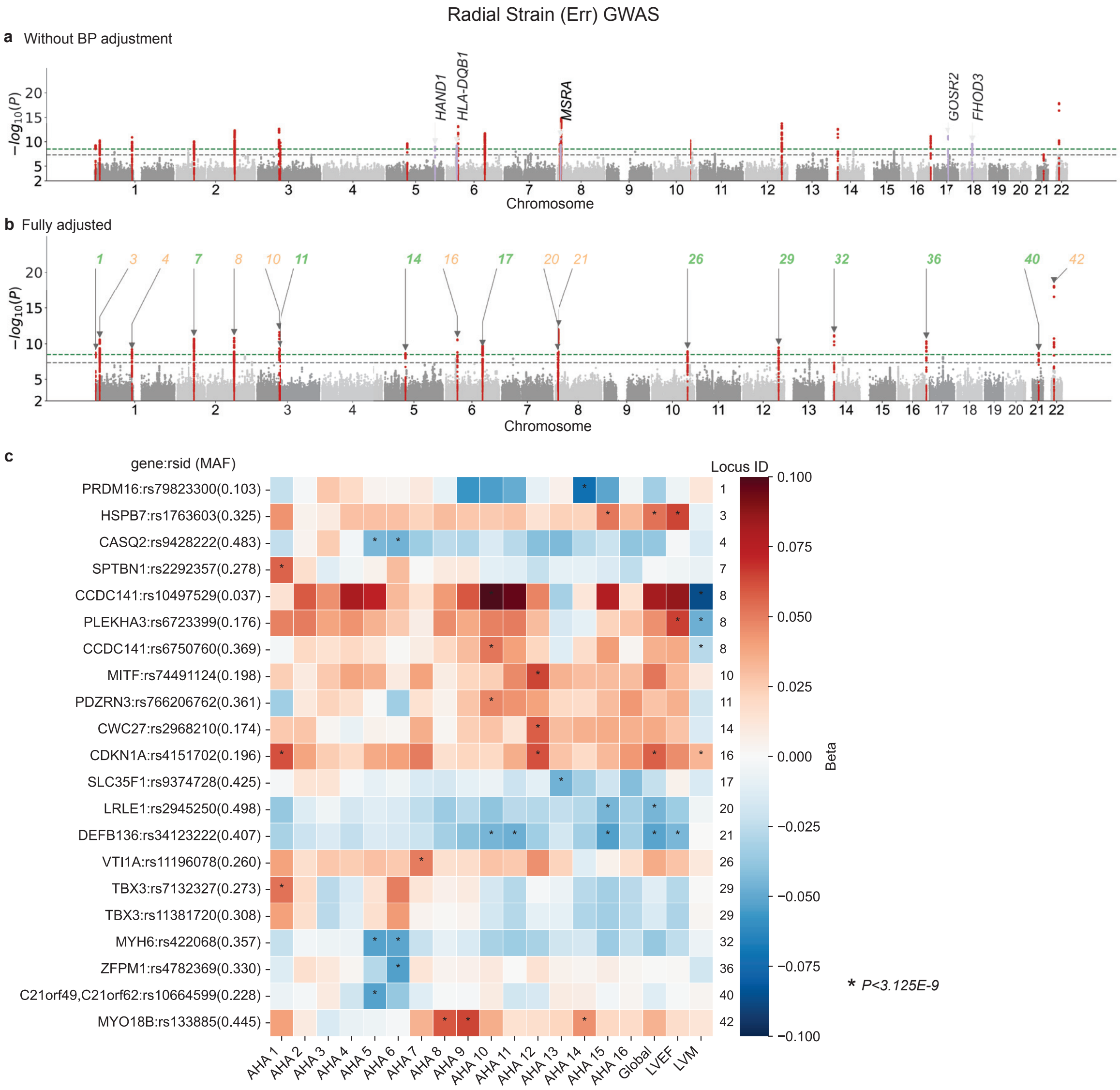

Supplementary Figure 9. Manhattan plots for the radial strain spatially resolved by the AHA-16 model.. (a) The radial strain is adjusted by Sex, age at MRI, age squared, BMI, BSA and the top 10 PCs. Minimum P value found by GWAS on 16 segments were shown in the manhattan plot. Highlights in red show the loci identified in fully adjusted GWAS (panel b), highlights in purple show additional loci that were no longer significant after adjustment with BP. These loci (purple) were annotated by the nearest gene. (b) The radial strain are additionally adjusted by SBP and DBP measured at MRI. (c) Heatmap showing the beta values of lead SNPs in each locus (by fully adjusted GWAS) with the AHA segments on LV.

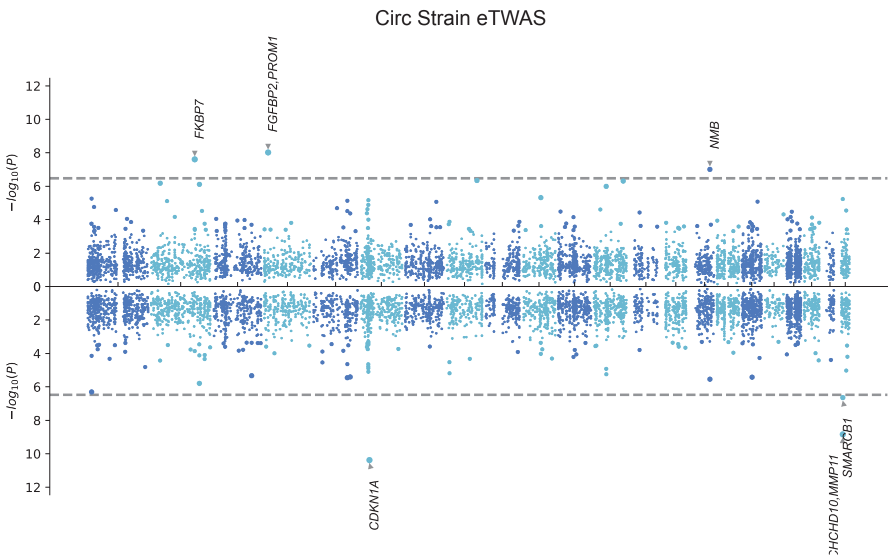

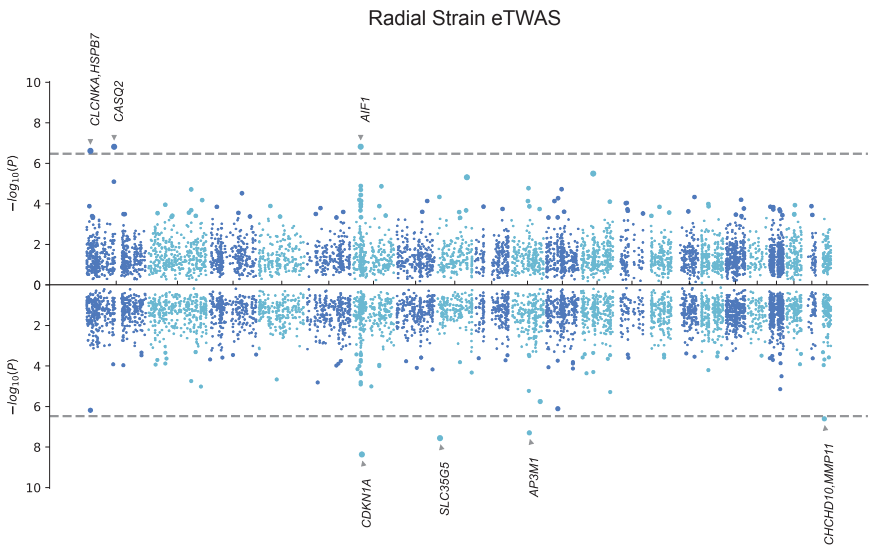

Supplementary Figure 10. Predicted regulatory effects of GWAS variants on expression. Regulatory effects were calculated using GWAS summary statistics and GTEx v8 eQTL MASH-R model for the heart left ventricle. 10,498 genes were tested. Manhattan plot shows the minimum P-value on the 16 regional traits of strain^{circ} and strain^{rad}.

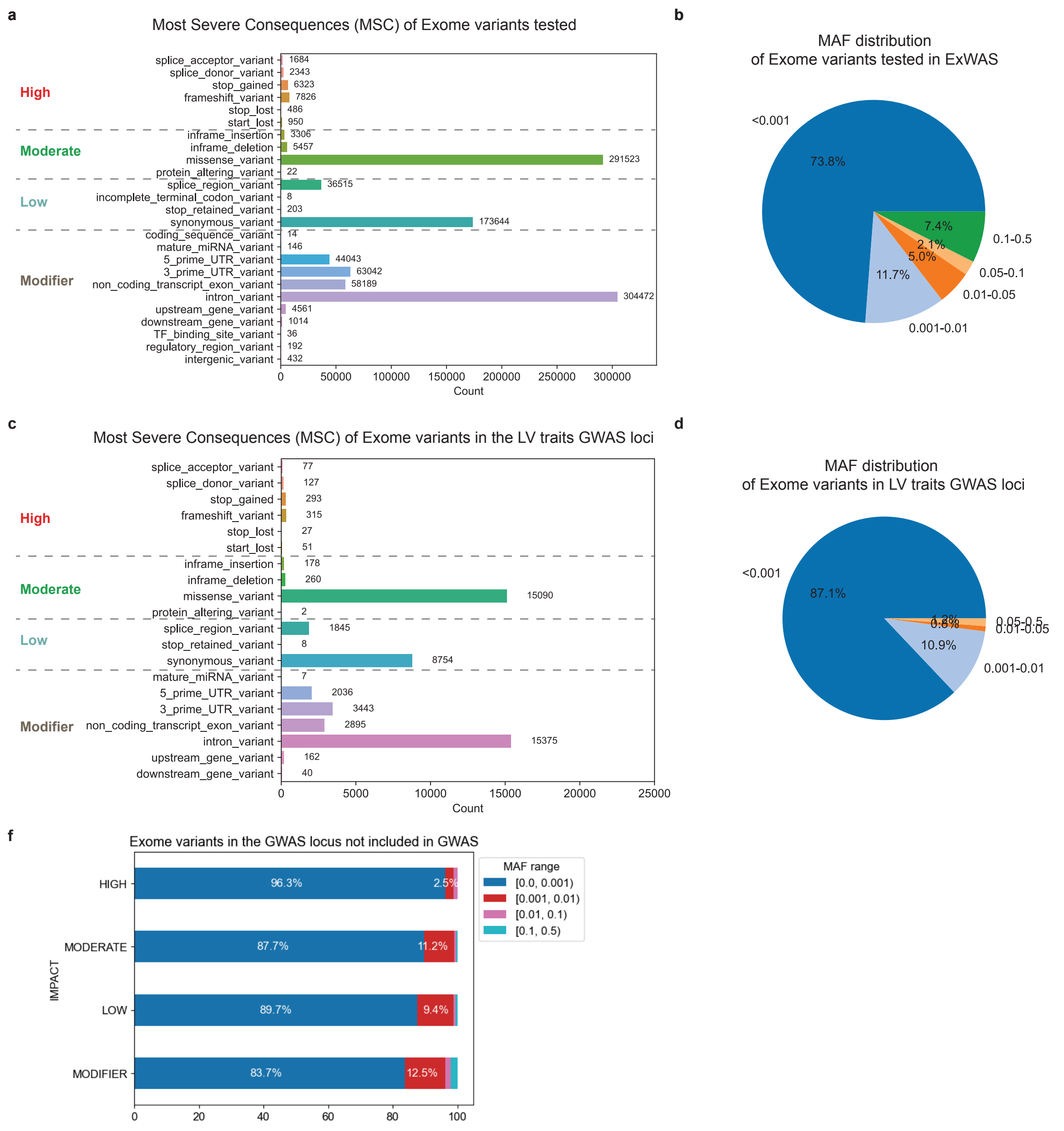

Supplementary Figure 11. Variant effect annotations of whole Exome data in the UKBB CMR imaging cohort. (a) Number of variants tested in ExWAS by the most severe consequence (MSC) annotated with Ensembl Variant Effect Predictor (VEP). 1,006,431 Exome variants that appeared at least five times in the UKBB CMR cohort were tested. (b) Distribution of the variants tested by minor allele frequency (MAF).

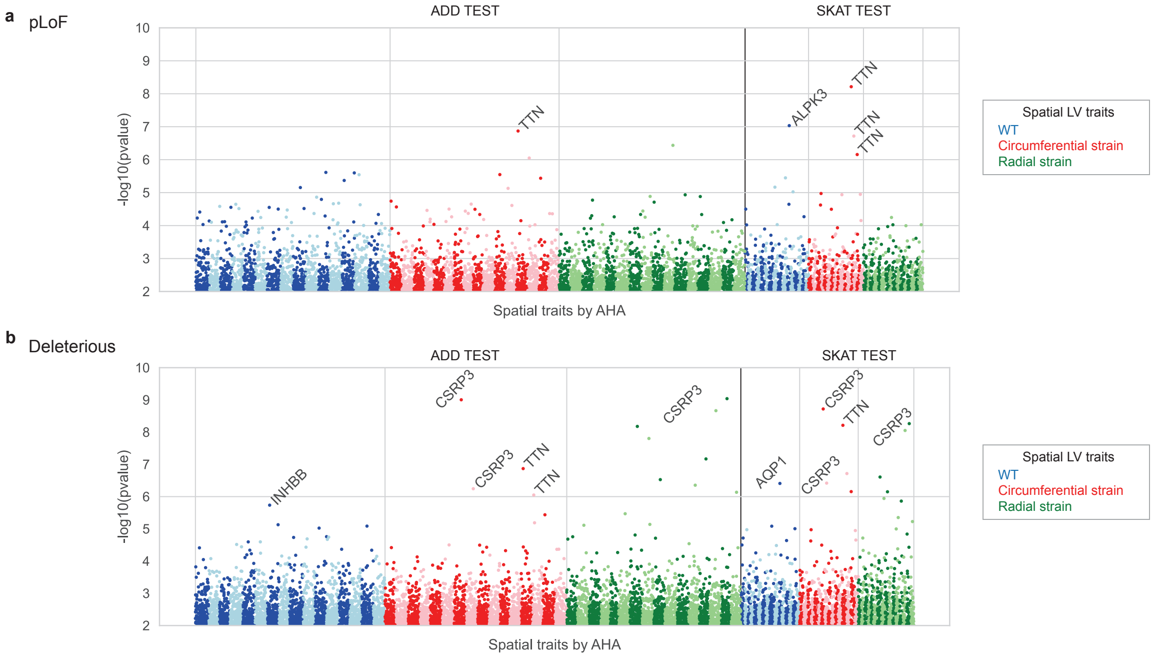

Supplementary Figure 12. Exome-wide gene-based tests on spatial LV traits. (a) Genes habouring more than five predicted loss-of-function (pLoF) variants in the cohort were tested for association with 48 spatial LV traits including wall thickness (blue), circumferential (red) and radial strain (green). Tests were performed using regular burden tests (ADD) and the variance component test (SKAT). ADD tests on pLoF variants were conducted for 10,768 genes, and SKAT for 3,666 genes. Alternating colors represent different AHA segments. Each dots represent the log-scaled P value of the gene association significance, and less genes were tested in SKAT. (b) Genes harbouring more than five predicted deleterious variants were tested. Similarly, 48 spatial LV traits were tested and alternating colors represent different AHA segments. ADD tests on pLoF variants were conducted for 15,924 genes, and SKAT for 9,732 genes. AHA: American Heart Association segment model. WT: wall thickness. SKAT: sequencing kernel association test.

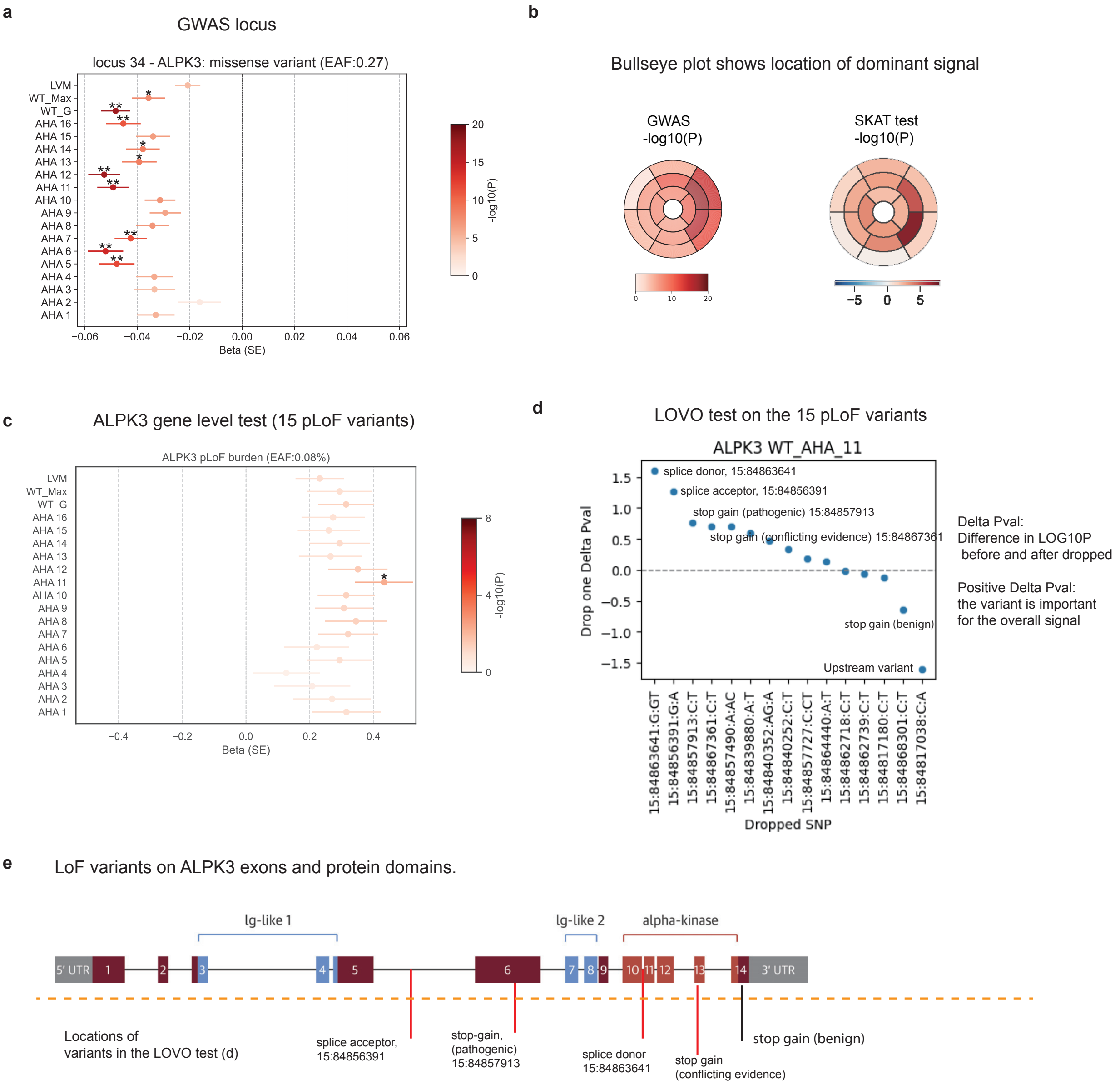

Supplementary Figure 13. Combined GWAS lead and rare leave-one-variant-out analysis of ALPK3. (a) Sentinel SNP in the GWAS locus and association values (beta) to global and spatial LV wall thickness, maximum wall thickness and LV mass. The minor allele is associated with decreased WT. (b) Bullseye plot that shows the P values of GWAS variant and SKAT burden tests. (c) The beta values of the ADD burdent tests, the pLoFs together were associated with increased WT. (d) Leave one variant out test on ALPK3, demonstraing different direction of effect from these variants. (e) the location of the variants on the ALPK3 domain structure. The gene scheme was duplicated from Almomani et.al., Figure 3[63]. The top four variants annotated in (d,e) from top to bottom are: splice donor rs753084997 (15:84863641), splice acceptor rs761330284 (15:84856391), stop gain (ClinVar pathogenic) rs749465164 (15:84857913), and stop gain rs541612157 (15:84867361).

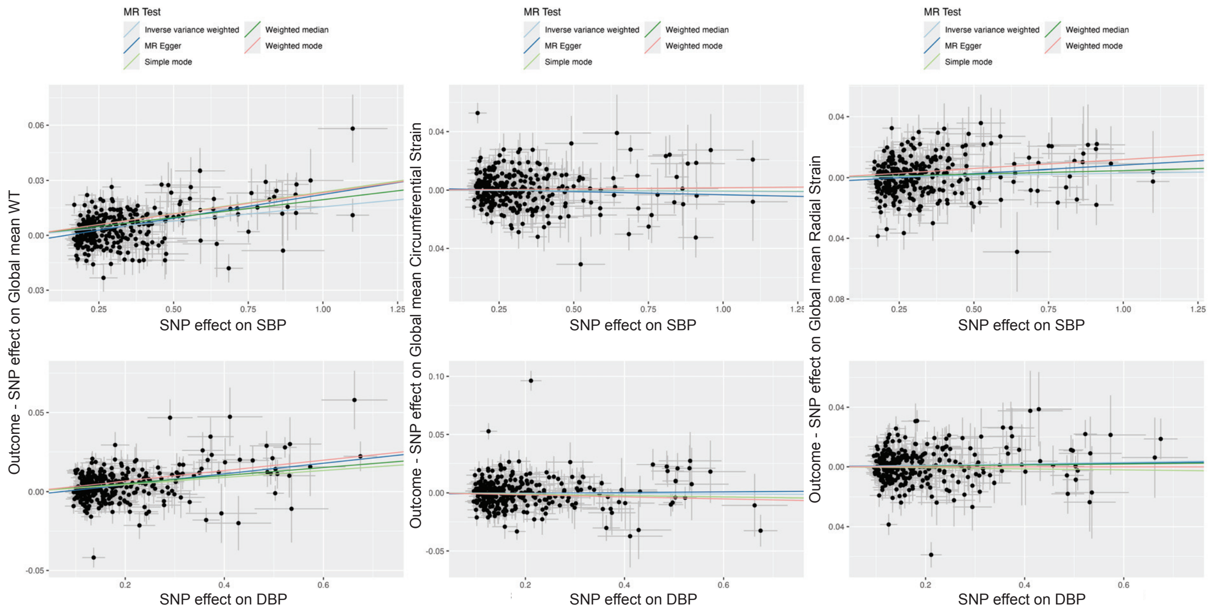

Supplementary Figure 14. Single nucleotide polymorphism (SNP) effects of blood pressure on global LV traits. Mendelian randomisation (MR) analysis of systolic (SBP) and diastolic (DBP) blood pressure as exposure (by row), global LV traits including the mean wall thickness, mean strain^{circ} and mean strain^{rad} as outcome (by column). Genetic instruments for SBP and DBP were selected from published GWAS[13]. The effects (β) of the exposure variable-increasing allele at independent SNPs ($r^2 < 0.001$) reaching $P < 5e\text{-}8$ are plotted as data points and associated standard errors are represented as lines extending from data points. The plots were produced using the R package `TwoSampleMR`. See Supplementary Data File 'Mendelian Randomisation.xlsx' for full MR results.

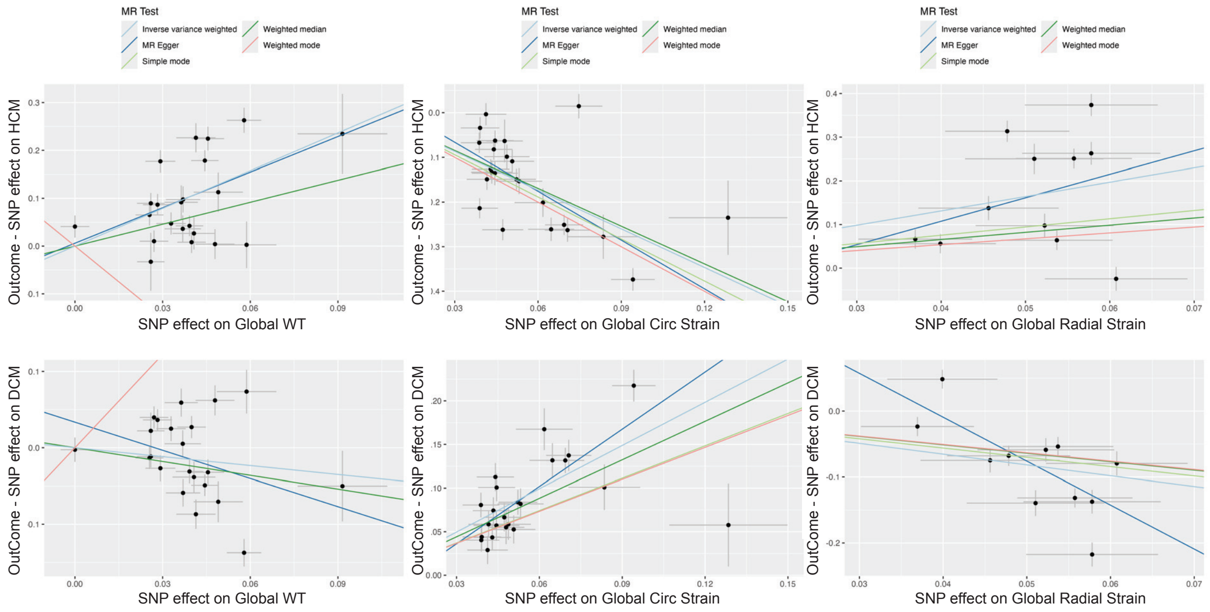

Supplementary Figure 15. Single nucleotide polymorphism (SNP) effects of global LV traits on HCM and DCM. Mendelian randomisation (MR) analysis of global LV mean wall thickness, mean strain^{circ} and mean strain^{rad} as exposure (by column), HCM and DCM as outcome (by row). The effects (β) of the exposure variable-increasing allele at independent SNPs ($r^2 < 0.001$) reaching $P < 5e\text{-}8$ are plotted as data points and associated standard errors are represented as lines extending from data points. Outcome were assessed with HCM[15] and DCM[16] GWAS. The plots were produced using the R package `TwoSampleMR`. See Supplementary Data File 'Mendelian Randomisation.xlsx' for full MR results.

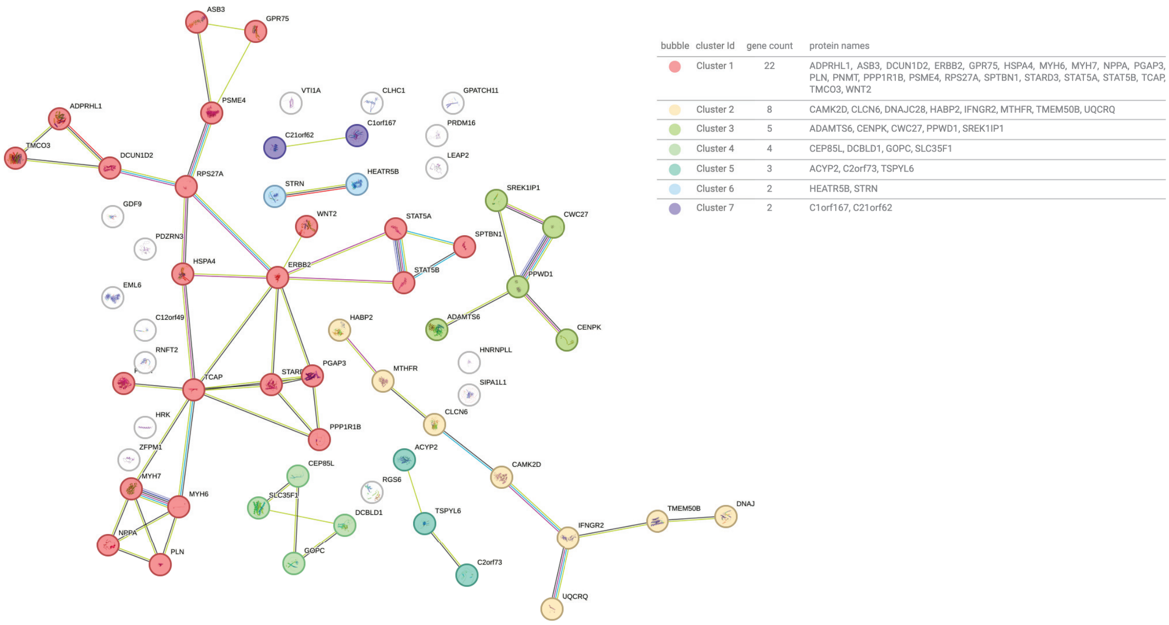

bubble	cluster Id	gene count	protein names
	Cluster 1	22	ADPRHL1, ASB3, DCUN1D2, ERBB2, GPR75, HSPA4, MYH6, MYH7, NPPA, PGAP3, PLN, PNMT, PPP1R1B, PSME4, RPS27A, SPTBN1, STARD3, STAT5A, STAT5B, TCAP, TMCO3, WNT2
	Cluster 2	8	CAMK2D, CLCN6, DNAJC28, HABP2, IFNGR2, MTHFR, TMEM50B, UQCRQ
	Cluster 3	5	ADAMTS6, CENPK, CWC27, PPWD1, SREK1IP1
	Cluster 4	4	CEP85L, DCBLD1, GOPC, SLC35F1
	Cluster 5	3	ACYP2, C2orf73, TSPYL6
	Cluster 6	2	HEATR5B, STRN
	Cluster 7	2	C1orf167, C21orf62

Supplementary Figure 16. STRING analysis for protein coding genes in the spatial-only loci. Protein coding genes from 18 spatial only GWAS loci were analysed using STRING database, k-means clustering identified seven clusters of interaction networks.

Supplemental Datasets

List of the Supplementary Data Files provided in the zip file.

- Supplementary Data File 1. LDSC Genetic Correlation full results.
- Supplementary Data File 2. GREML heritability and fixed effect variance full results.
- Supplementary Data File 3. PheWAS full results.
- Supplementary Data File 4. Spatial LV GWAS loci and gene prioritisation table.
- Supplementary Data File 5. Exome wide gene based burden tests significant associations.
- Supplementary Data File 6. Bidirectional Two-Sample Mendelian Randomisation full results.
- Supplementary Data File 7. Gene lists used in STRING-DB analysis and the list of enriched pathways.
